# Reversal gene expression assessment for drug repurposing, a case study of glioblastoma

**DOI:** 10.1186/s12967-024-06046-1

**Published:** 2025-01-07

**Authors:** Shixue Sun, Zeenat Shyr, Kathleen McDaniel, Yuhong Fang, Dingyin Tao, Catherine Z. Chen, Wei Zheng, Qian Zhu

**Affiliations:** 1 Informatics Core, Division of Pre-Clinical Innovation, National Center for Advancing Translational Sciences (NCATS), National Institutes of Health (NIH), Rockville, MD USA; 2 Early Translation Branch, Division of Pre-Clinical Innovation, National Center for Advancing Translational Sciences (NCATS), National Institutes of Health (NIH), Rockville, MD USA; 3 Analytical Chemistry Core, Division of Pre-Clinical Innovation, National Center for Advancing Translational Sciences (NCATS), National Institutes of Health (NIH), Rockville, MD USA

**Keywords:** Rare diseases, Drug repurposing, Glioblastoma, Multi-omics analysis, Reversal gene expression

## Abstract

**Background:**

Glioblastoma (GBM) is a rare brain cancer with an exceptionally high mortality rate, which illustrates the pressing demand for more effective therapeutic options. Despite considerable research efforts on GBM, its underlying biological mechanisms remain unclear. Furthermore, none of the United States Food and Drug Administration (FDA) approved drugs used for GBM deliver satisfactory survival improvement.

**Methods:**

This study presents a novel computational pipeline by utilizing gene expression data analysis for GBM for drug repurposing to address the challenges in rare disease drug development, particularly focusing on GBM. The GBM Gene Expression Profile (GGEP) was constructed with multi-omics data to identify drugs with reversal gene expression to GGEP from the Integrated Network-Based Cellular Signatures (iLINCS) database.

**Results:**

We prioritized the candidates via hierarchical clustering of their expression signatures and quantification of their reversal strength by calculating two self-defined indices based on the GGEP genes’ log2 foldchange (LFC) that the drug candidates could induce. Among five prioritized candidates, in-vitro experiments validated Clofarabine and Ciclopirox as highly efficacious in selectively targeting GBM cancer cells.

**Conclusions:**

The success of this study illustrated a promising avenue for accelerating drug development by uncovering underlying gene expression effect between drugs and diseases, which can be extended to other rare diseases and non-rare diseases.

**Supplementary Information:**

The online version contains supplementary material available at 10.1186/s12967-024-06046-1.

## Introduction

Low prevalence and the increasing number of rare diseases brings a substantial challenge for the study of disease etiology and the development of pharmaceutical interventions. Of the over 10,000 rare diseases affecting 30 million individuals in the US, only about 500 rare diseases have FDA-approved treatments [1]. Glioblastoma (GBM), a rare type of highly aggressive brain cancer, is characterized by its devastatingly short survival time due to the absence of effective treatments. GBM is associated with an exceptionally high mortality rate, with roughly 30% of patients surviving only one year and less than 5% surviving five years [2]. This stark reality underscores the pressing demand for more effectively therapeutic options. Despite considerable research efforts on GBM, its underlying biological mechanisms remain unclear. Presently, the United States Food and Drug Administration (FDA) has approved four drugs for GBM, none of which deliver satisfactory survival improvement, underscoring the imperative for innovative therapies [3].

Drug repurposing (DR), the discovery of existing drugs for new therapeutic use, emerges as a promising strategy for drug development [4, 5]. DR leverages the existing data on safety profiles, pharmacokinetics, and mechanisms of action of approved drugs, and thus can be a time and cost-effective alternative to traditional de novo drug development [6]. By circumventing early-phase clinical trials and drug safety assessment, DR can significantly shorten the average development timeline from approximately 12 years to about 7 years [7]. For instance, Hutchinson-Gilford progeria syndrome (HGPS) and Muckle-Wells syndrome (MWS) are two rare diseases with successful DR candidates, identified based on the pairing of cellular pathophysiology mechanisms and the drug’s mechanism of action. Farnesyltransferase inhibitors (FTI), originally used for cancer treatment, showed therapeutic effect on HGPS, a rare premature aging disease, in which protein farnesylation plays a critical role, leading to the recent application for FDA approval as the first ever treatment for HGPS [8]. Canakinumab, a human IgG1 anti-IL-1β monoclonal antibody initially approved for rheumatoid arthritis, has been successfully repurposed for MWS, an autoinflammatory rare disorder caused by increased IL-1 [9].

With the current explosion of omics data reservoirs, which include genetics, transcriptomics, proteomics, and metabolomics datasets, computational method to uncover underlying biological mechanisms plays an important role in DR. Concurrently, substantial datasets concerning drugs’ perturbation on gene expression of disease cell line models are increasingly applied in DR [10], exemplified by resources like the Connectivity Map (CMap) [11], LINCS [12], and iLINCS[13]. Thus, linking drug responses and disease gene expression emerges as a promising strategy for DR. For example, via CMap-based transcriptome analysis, ivermectin has been identified as a new oncotherapy candidate for gastric cancer and its effect has been validated in wet-lab experiments [14]. Furthermore, targeting these databases, gene expression signature-based screening approaches, such as reversal gene expression identification [15], have been proposed to identify DR candidates [16, 17]. For those feature genes that exhibit misregulation in a disease, a reversal gene expression is defined when they were regulated in the opposite direction (upregulation vs. downregulation) in cell lines treated with a drug.

Although systematic approaches based on reversal gene expression have yielded promising DR candidates for cancers and several other common diseases [18], its application had not been reported in rare diseases. Therefore, in this study we adopted the aforementioned concept of reversal gene expression [15] to identify DR candidates for GBM by leveraging gene expression signature. Specifically, we constructed a GBM gene expression profile (GGEP) through an integrated differential gene expression analysis of transcriptome and proteome, aiming for an optimal characterization of GBM’s mechanism. Targeting this GGEP we identified DR candidates with reversal gene expression signatures, the therapeutic effects of which were validated via cell viability assessment in GBM cell lines and control astrocytes. This omics-based DR approach illustrates the potential to significantly advance DR efforts in rare diseases and certainly common diseases as well.

## Methods and materials

**Fig. 1:**
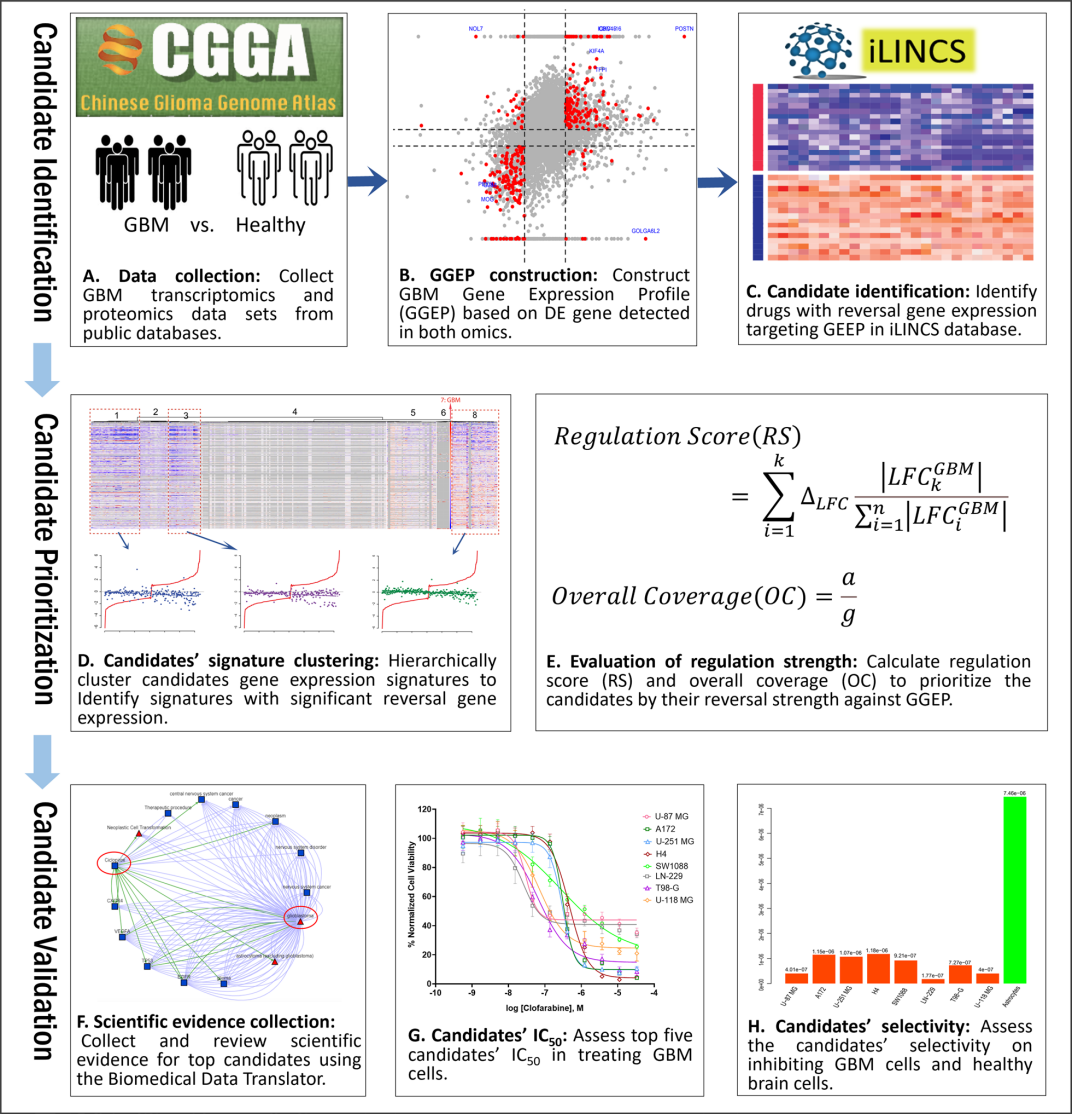
Study workflow

In this study, we attempted to integrate transcriptomics and proteomics for GBM gene expression profile (GGEP) construction toward DR. The drug candidates identified with significant reversal gene expression were evaluated from multiple aspects to identify the top potential repurposing candidates. Figure 1 illustrates the study workflow comprising of four main components, candidate identification based on reversal gene expression (A, B and C), candidate prioritization assessed regarding the reversal strength (D and E), candidate evaluation with the identified scientific evidence (F), and experimental evaluation (G and H). We describe each of the components in the following sections.

## Drug candidate identification with reversal gene expressions to GBM

### GBM based multi-omics data preparation

We collected transcriptome and proteome datasets from the Chinese Glioma Genome Atlas (CGGA) database [19] and an academic research paper [20] by following two criteria: (1) utilizing human brain tissue samples from GBM patients, and (2) conducting experiments on the same or similar platforms with analogous methodologies.

In this study, we utilized message RNA (mRNA) sequencing datasets collected from CGGA. Compared to total RNA transcriptomics, mRNA sequencing focuses on protein-coding genes which are translated into proteins. Proteomics data sets were derived from the experiment conducted by Buser et al. [20], which encompassed three GBM samples and three control samples. To the best of our knowledge, this experiment stands as the sole source of proteomics data that compared healthy control tissues and provided accessible original protein intensities. We downloaded the read counts for each gene from the mRNA sequencing and the signal intensities for each identified protein from proteomics experiments.

Principal component analysis (PCA) [21] was employed to estimate the similarity between each sample’s gene expression profiles. Samples as outliers were excluded from the dataset. The PCA was performed using the R package DEseq2 [22].

### Differential expression identification and GGEP construction

We identified differentially expressed (DE) genes from both transcriptome and proteome datasets. A DE mRNA expression was identified as Benjamini-Hochberg (B-H) [23] adjusted *p*-value < 0.05 and absolute log_2_ foldchange (|LFC|) > 1. DE genes in the transcriptome datasets were determined via the standard procedure with the R package Deseq2. A DE protein translation was defined as Bonferroni [24] adjusted *p*-value < 0.05 and |LFC|> 1. DE proteins were identified from the proteome data set using the R stats package [25]. As the LFC cannot be calculated for proteins that were detected only in one group, we manually set their fold changes as a fixed value which approximates the maximum fold change detected in the experiment. Thereby we included these proteins with significant impacts on GBM. Based on the identified DE genes, we then constructed a GBM gene expression profile (GGEP) comprising genes exhibiting both DE mRNA and DE protein expression in GBM.

### Identification of drug candidates with reversal responses in the iLINCS database

We searched the iLINCS database [13] for drug responses that demonstrate reversal effect to GGEP. The iLINCS defines a signature as the cell line's gene expression when perturbated by a particular chemical or drug. A signature was captured for each perturbation experiment. In this study we queried multiple signature libraries in iLINCS, including Cancer therapeutics response signatures [26], LINCS Chemical perturbagen signatures (LINCS L1000 assay) [12], Connectivity Map signatures [27], DrugMatrix signatures [28], Pharmacogenomics transcriptional signatures [29, 30], and LINCS target proteomics signatures [31]. The iLINCS auto-generated Pearson’s correlation coefficient (i.e., the concordance), was used as an index for preliminary identification of reversal drug response signatures to GGEP. A negative concordance value indicates that the chemical-induced gene expression was inversely correlated with the GGEP [13]. To include all potential candidates, we selected chemicals that induced gene expression signatures of a concordance score < − 0.2 [32]. Among these chosen chemicals, only FDA-approved drugs [33] (Published on June 6th, 2023) were included for further analysis towards DR.

## Drug candidate prioritization

In the previous step, we identified drugs that could induce gene expression signatures that inversely correlated with the GGEP. In this step, we assessed the candidates’ reversal strength via similarity clustering of their gene expression signatures and calculation of two self-defined evaluation indices. In addition, we collected Blood–Brain Barrier (BBB) permeation probabilities of those candidates from the DrugBank database [34] to consider sufficient drug uptake in the brain.

### Candidates’ gene expression signature clustering

We retrieved gene expression signatures of the candidates from the iLINCS via its API [35], utilizing R packages *knitr* [36], *tinytex* [37], *httr* [38], *jsonlite* [39], *htmltools* [40], and *Biobase* [41]. Subsequently, we clustered these signatures based on their expression features using the *ComplexHeatmap* R package [42]. The matrix used for this clustering is DEG’s LFC in each signature. The parameters used for the clustering are the Minkowski distances and Ward's hierarchical cluster method [43]. Heatmap was employed to categorize the drugs’ response signatures based on the similarity between their reversal gene expression and GGEP.

### Regulation strength calculation

To quantify the candidates’ regulation strength, we defined two indices, regulation score (RS) and overall coverage (OC) based on the number of genes in the GGEP they regulate and the LFCs of reversed gene expression they can produce respectively.

#### Regulation score (RS)

Based on the concept of Kullback–Leibler (KL) divergence [44], we introduced the RS which quantifies the regulation strength (i.e., LFC) based on the divergence between the GGEP and drug response signature (Formula 1). The RS is positively correlated with (1) the number of GBM-related genes it regulates, (2) the strength it regulates these GBM-related genes (LFC in the expression signature), and (3) the importance of the GBM-related genes it regulates (LFC in the GGEP). Thus, a potential drug candidate would be associated with a high RS, which illustrates its strong reverse effects on the expressions over GGEP genes.

1 $$RS= \sum_{i=1}^{k}{\Delta }_{\mathit{LFC}}\frac{|{LFC}_{k}^{GBM}|}{\sum_{i=1}^{n}|{LFC}_{i}^{GBM}|}$$

, where

$${\Delta }_{LFC}= \left\{\begin{array}{c}\left|\left({LFC}_{k}^{drug}-{LFC}_{k}^{GBM}\right)\right|, if {(LFC}_{k}^{drug}{LFC}_{k}^{GBM})<0\\ 0, if {(LFC}_{k}^{drug}{LFC}_{k}^{GBM})>0\end{array}\right.$$

The ${LFC}_{k}^{GBM}$ and ${LFC}_{k}^{drug}$ stand for the LFC of gene *k* in the gene expression feature of GBM and drug response signature, respectively. Theoretically, RS is a positive value ranges [0, + ∞). The derivation and interpretation of RS can be found in the supplementary file named “supp file 01.docx”.

#### Overall coverage (OC)

We defined an OC (formula 2) as the ratio of GBM-related genes regulated by drug candidates. OC is defined as the percentage of the GGEP genes, whose gene expression could be reversed by a single drug. The OC was calculated following below formulas:

2 $$OC=\frac{a}{g}$$

In formulas (2), *‘g’* stands the number of the GBM-associated genes in the GGEP, while *‘a’* denotes the GBM-associated genes regulated by drugs (Fig. 1G). OC has positive values, ranged [0,1]. A higher OC score indicates a higher ratio of GGEP genes that a treatment can reverse.

## Drug candidate validation

We evaluated the candidates with their possible mechanism of action in treating GBM in pre-clinical experiments and clinical trials via the Biomedical Data Translator [45] and the top five candidates were further validated in *in- vitro* experiments.

### Evaluation based on scientific evidence

We identified scientific evidence to further evaluate and prioritize drug candidates. First, we examined if these drug candidates have undergone clinical trials for GBM treatment. We queried ClinicalTrial.gov using the keywords "glioblastoma", "high-grade glioma", and “GBM” in the "condition" field to retrieve clinical trials in which the candidates have been used as intervention to treat GBM. In parallel, we also conducted literature search for candidates related clinical trials performed outside the US. Then, we explored their possible pharmacological mechanisms for GBM by collecting scientific evidence from the NCATS Biomedical Data Translator [45]. Specifically, we utilized the ARAX reasoning engine [46] part of the Translator eco-system to identify any possible direct and indirect correlations between the candidates and GBM. In the end, we identified five candidates, namely Ciclopirox, Prochlorperazine, Clofarabine, Tacrolimus, and Tigecycline with promising therapeutic effects that had not yet been investigated for clinical GBM use for further experimental evaluation.

### Therapeutic effect validation on drug candidates

#### Assessment of DR candidates’ half maximal inhibitory concentration (IC_50_)

Eight GBM cell lines were purchased from American Type Culture Collection (ATCC, Manassas, VA, USA) (A-172, H-4, U-87 MG, T98-G, SW-1088, LN-229, and U-118 MG) and Kerafast, Inc. (U-251 MG) (Shirley, MA, USA). All cell lines were cultured and maintained as recommended by the vendor. Seeding densities for each line were optimized in white, solid bottom 1536-well microplates (Greiner BioOne, Monroe, NC, USA) in 6 µL of media per well. Cells were plated using the Multidrop Combi Liquid Dispenser (Thermo Fisher, Waltham, MA, USA) at 200 cells/well except for U-87 mg, T-98 G, U-118 MG, which were plated at 400, 150 and 300 cells/well, respectively. The plates were incubated at 37 °C with 5% CO_2_ for six hours before adding compounds. Ten millimolar stock solution of above mentioned five candidate compounds were titrated in Dimethyl Sulphoxide (DMSO) at a 1:3 dilution in 384-well plates, which were then dispensed at 20 nL/well to 1536-well plates by Echo Acoustic Liquid Handling (Beckman Coulter, Inc., Brea, CA, USA). In addition to testing the candidates, temozolomide (TMZ), the FDA-approved chemotherapy drug for GBM, was included as a reference control. TMZ was tested at concentrations ranging from 8 nM to 500 µM. Cells were incubated at 37 °C with 5% CO_2_ with the compounds for 72 h before adding 4.5 μl of CellTiter-Glo luminescent reagent (Promega, Madison, WI, USA) per well. The plates were incubated at room temperature for 10 min before reading signal luminescence on PHERAStar plate reader (BMG Labtech, Cary, NC, USA). Data was normalized to cells with 0.3% DMSO (100% viability) and 10 µM Staurosporine (0% viability) as a positive control. Concentration–response curves with corresponding relative half-maximal inhibitory concentration (IC_50_) values were plotted and analyzed in GraphPad Prism 9 (GraphPad, Inc., San Diego, USA). All results are shown as means of eight biological replicates ± standard deviation (SD).

##### Selectivity assessment of ciclopirox and clofarabine

We found Ciclopirox and Clofarabine exhibited the best IC_50_ curves in the above experiment, thus, we further evaluated their selectivity between GBM cells and astrocyte cells. Specifically, iPSC-derived astrocytes (Fujifilm Cellular Dynamics, Cat#C1037) and all GBM lines were seeded in laminin-coated 35 µL media at 2400 cells/well in 384-well plates for 24 h at 37 °C with 5% CO_2_. Compounds were diluted in media before adding to the assay plate and further incubated for 72 h at 37 °C with 5% CO_2_. Prior to reading luminescence, the bottom of the plate was sealed with white backing tape (after visualization of cells). A mixture of 35 μL/well of CellTiter-Glo luminescent reagent was added to the plates and the signal was read as described above. Results are shown as means of four or six replicates ± standard deviation (SD).

##### Cell viability staining

GBM and astrocytes cell lines were plated in 1536 black clear bottom plates and treated with Ciclopirox and Clofarabine in parallel with plates for luminescence assays. After 72 h of incubation, cells were fixed with a final concentration of 4% paraformaldehyde (PFA) for 20 min at room temperature. Cells were washed with Phosphate-buffered saline (PBS) followed by incubation with 0.5 µg/mL of high-content screening CellMask green (Thermo Fisher Scientific) and 4 µM Hoechst 33342 (Thermo Fisher Scientific) at room temperature for 30 min. Cells were washed twice and sealed for imaging. Imaging was performed on the Opera Phenix High Content Screening System (Revvity, Inc).

## Results

### Results on identifying drugs with reversal gene expression

#### Results on multi-omics data preparation

Adhering to our inclusion criteria described in the Methods, we obtained mRNA-seq data sets from three projects from the CGGA, containing 358 GBM patients and 20 healthy brain tissues. By performing the PCA, thirty outliers (supplemental Figure S1) were excluded from the subsequent DE analysis. We downloaded proteome datasets of three GBM samples and three control samples from Buser et al.’s study [20]. GBM samples were extracted and pooled from eight GBM patients, while control samples were extracted and pooled from five epileptic patients. There are no outliers identified in the proteome data sets thus all samples were included in the DE analysis (supplementary Figure S2). Table 1 lists clinical distribution about patient subjects from the transcriptomics study involved in this study. A complete clinical characteristics about the patients from both omics’ datasets can be found in the supplementary file named “supp file 02.xlsx”.

**Table 1:** Basic information of patient subjects from the transcriptomics study

	Female	Male	Overall
	(N = 135)	(N = 193)	(N = 328)
GBM type			
Primary	79 (58.5%)	119 (61.7%)	198 (60.4%)
Recurrent	56 (41.5%)	74 (38.3%)	130 (39.6%)
Age			
Mean (SD)	48.9 (12.7)	48.3 (13.6)	48.5 (13.2)
Median [Min, Max]	50.0 [19.0, 72.0]	49.0 [11.0, 79.0]	50.0 [11.0, 79.0]
Overall Survival (Day)			
Mean (SD)	605 (696)	628 (649)	618 (668)
Median [Min, Max]	366 [27.0, 4440]	405 [19.0, 3820]	387 [19.0, 4440]
Missing	4 (3.0%)	6 (3.1%)	10 (3.0%)

### Results on DE gene analysis and GGEP construction

DE analysis of transcriptome datasets revealed 7,106 upregulated and 5,359 downregulated transcripts in GBM. DE analysis of proteome datasets identified 890 upregulated and 309 downregulated proteins in GBM. Table 2 shows calculated values of DE genes for both omics from raw data.

**Table 2:** DE analysis results

	Transcriptomics				Proteomics			
	GBM	Control	LFC	*p*-adj	GBM	Control	LFC	*p*-adj
CDC45	285.1	20.9	3.06	1.67E-23	6.82E5	0	NA	0.011
NOL7	176.9	1082.1	− 3.37	3.95E-197	2.24E6	0	NA	6.33E-05
TFPI	685.2	65.5	2.75	2.53E-19	5.63E6	3.6E4	7.30	0.041
PEX5L	678.2	2861.4	− 2.85	4.64E-10	3.33E5	3.23E7	− 6.60	0.0078
GOLGA6L2	29.1	2.5	4.94	2.83E-09	0	1.20E7	NA	0.017

*p*-adj refers to adjusted p-values of the hypothesis test of mean gene expression level in GBM and control groups. The Transcripts’ LFC in this table were calculated after transformation and normalization of all genes’ read counts using the R package DEseq2

**Fig. 2:**
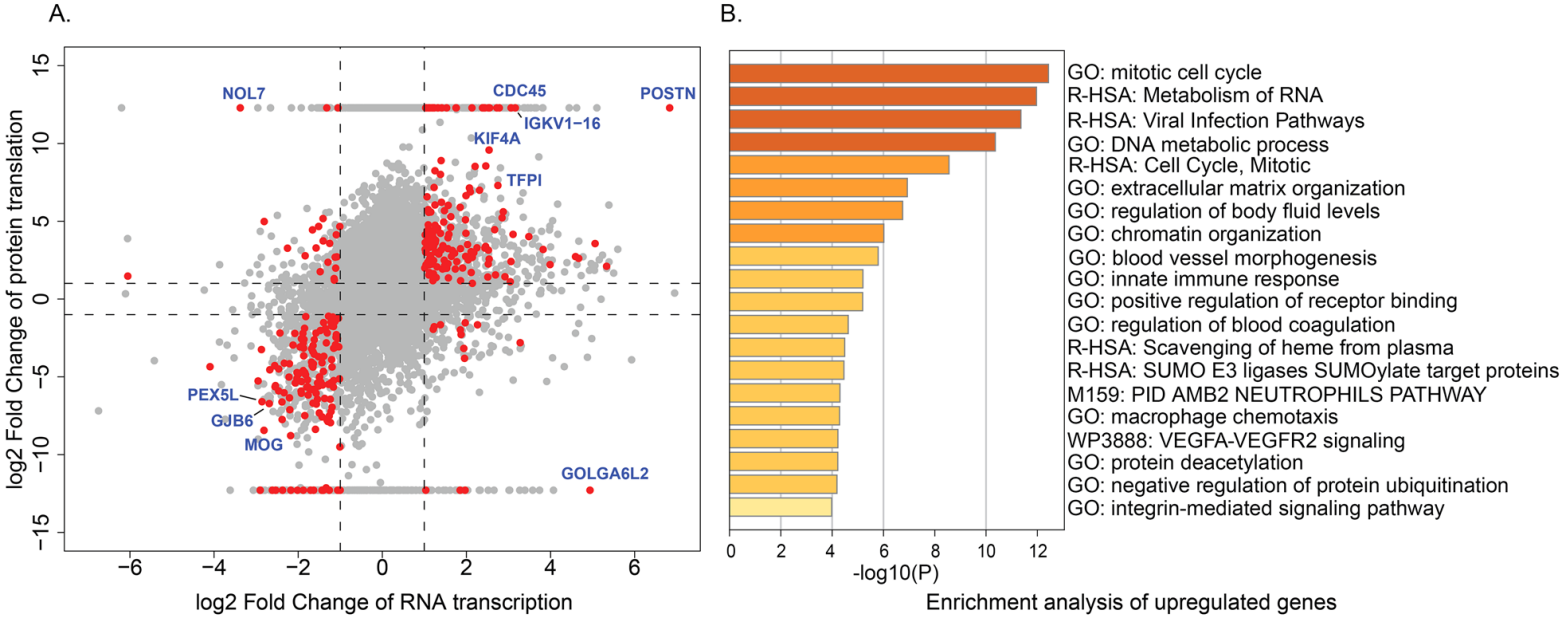
Expression and enrichment analysis of DE genes in the GGEP. **A** GBM gene expression at both transcription and translation stages. Each dot stands for one gene, with its LFCs in RNA transcription and protein translation displayed in the X and Y axes, respectively. Red dots denote the 318 DE genes applied to construct the GGEP. **B** Enrichment analysis results of upregulated genes in the GGEP. Each bar denotes an enriched pathway from GO. The X-axis values are the log-transformed enrichment p-values

Combining these two sets resulted in 318 DE genes that exhibit significant regulation across both transcription and protein translation processes (Fig. 2A). Subsequently, we constructed the GGEP using the LFCs of these 318 genes transcription expression in GBM. The raw data and DE analysis results of both omics were provided as supplementary file named “supp file 03.xlsx”. In the GGEP, the top ten DE genes ranked by the LFC and adjusted p-value are associated with tumorigenesis (CDC45 [47, 48], POSTN [49], KIF4A [50, 51], PEX5L [52], TFPI [53], GOLGA6L2 [54], NOL7 [55], GJB6 [56, 57], IGKV1-16 [58], and MOG [59]). For instance, CDC45 is associated with DNA methylation in a variety of cancers and its expression is negatively correlated with overall survival of GBM [48]. POSTN, a matricellular protein implicated in gliomas and ovarian cancer, drives tumor growth and metastasis, influences cell responses [49], and could serve as a potential biomarker for GBM survival prognosis [60]. NOL7, positioned on chromosome 6p23, exhibits dual roles of suppressing cervical carcinoma cell growth while promoting melanoma progression [55]. As shown in Fig. 2B, the DE genes in GGEP are enriched with cell proliferation-related GO terms and pathways (cell cycle, RNA metabolism, DNA metabolic processes, etc.) which reflect the excessive cell proliferation in tumor progression [61, 62]. Notably, the enrichment of VEGFA-VEGFR2 signaling pathway, a major driver of tumor angiogenesis and metastasis indicates its prominent role in GBM mechanism. This pathway is instrumental in angiogenesis, fostering endothelial cell activities and vascular permeability, rendering it a promising target for therapy development across diverse cancers, including glioblastoma [63–65].

### Results on identification of drugs with reversal gene expression

As shown in Table 3, 1517 gene expression signatures were identified from iLINCS by applying the predefined Concordance cutoff, calculated between the GGEP and the drug response signatures. These signatures were derived from perturbation experiments of 726 chemicals, which include 119 FDA-approved drugs. Detailed information of these signatures and chemicals can be found in the supplemental file named “supp file 04.csv”.

**Table 3:** Drugs identified in iLINCS with reversal gene expression signatures

		Cutoff	Signatures	Chemicals	Drugs
Signature Libraries	Cancer therapeutics response	< − 0.2	431	275	37
	LINCS Chemical perturbagen	< − 0.6	325	263	15
	Connectivity Map	< − 0.2	14	11	5
	DrugMatrix	< − 0.2	337	187	164
	Pharmacogenomics transcriptional	< − 0.2	377	26	126
	LINCS target proteomics	< − 0.2	33	32	3
Total		–	1517	794	350
Unique		–	–	726	119

The Cutoff column lists the Concordance score value used to filter the signatures with reversal gene expressions. A negative concordance denotes a possible reversal gene expression to GGEP. After the first-round screening using concordance < − 0.2 as a cutoff, we further strain the cutoff to < − 0.6 for the LINCS Chemical perturbagen library. This is based on the observation that much lower numbers of overlapped genes between its signatures and GGEP (approximately 10% of other signatures), which will increase false positive rate. The Signatures column lists the number of signatures identified in each signature library following the cutoffs. The Chemicals column lists the number of chemicals tested in these signatures. The Drugs column denotes the number of FDA-approved drugs identified accordingly. The row of Total denotes the numeric sum of signatures, chemicals, and drugs identified from all libraries, while the row of Unique lists the unique numbers of chemicals and drugs

Twenty-one of these 119 drugs have undergone investigation in 215 GBM related clinical trials resulted by searching ClinicalTrials.gov. Temozolomide (TMZ), as one of 21 drugs, has been studied in 169 clinical trials. The remaining 20 drugs have been investigated by an average of 2.3 trials. Dasatinib, Sirolimus, Hydroxyurea, and Etoposide, appeared in five GBM based clinical trials individually. Notably, among the 21 drugs, there are three Vascular Endothelial Growth Factor Receptor 2 (VEGFR2) inhibitors (Axitinib, Cabozantinib, and Dasatinib) and one EGFR inhibitor (Gefitinib). This observation proved the significance of the Vascular Endothelial Growth Factor A (VEGFA)-VEGFR2 signaling pathway in GBM progression, which was highlighted in the GGEP enrichment analysis (Fig. 2B), and thus targeting this pathway provides a promising research direction in the development of GBM treatment strategies. That being said, identification of these 21 drugs proved our methodology is valuable for DR, and remaining 98 drugs might be novel drug candidates for GBM to be examined. The detailed information of the 119 drugs can be found in the supplementary file named “supp file 05.csv”.

## Results on drug candidate prioritization

### Gene expression signatures clustering results

**Fig. 3:**
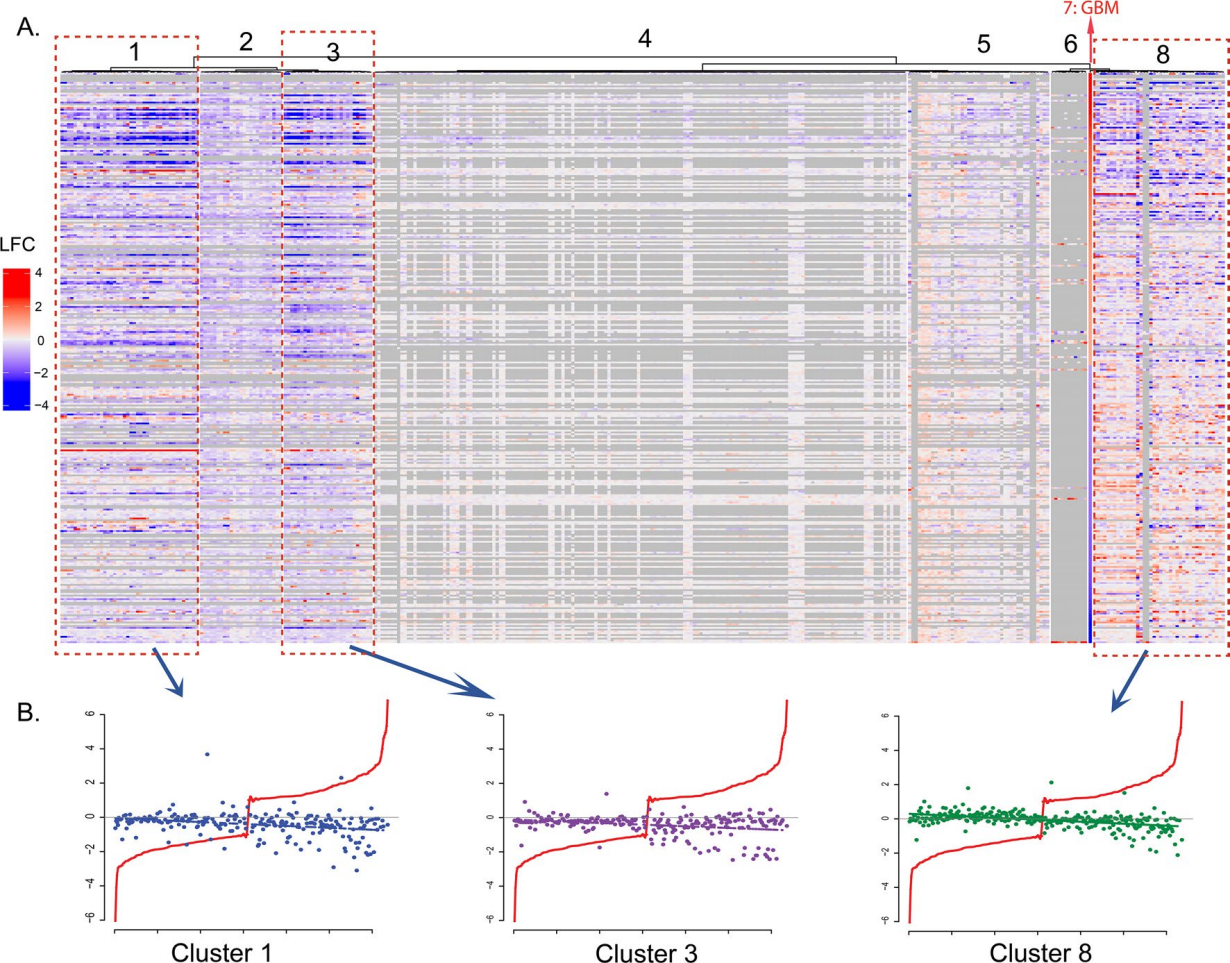
Classification of perturbation signatures. **A** The perturbation gene expression signatures were classified into 7 clusters. Each row corresponds to a gene in GGEP while each column corresponds to one perturbation experiment signature. **B** Scatter plots of signature LFCs in cluster #1, 3, and 8. Each dot represents the LFC of one gene in one signature. The colors of dots denote different clusters. The X-axis presents the genes in GGEP, while the Y-axis presents their corresponding LFCs. The red line denotes the LFCs in GGEP in ascending order

The 350 gene expression signatures of the 119 drugs were categorized into seven clusters with different reversal gene expression patterns, shown as cluster 1–6, and 8 in Fig. 3A (Cluster 7 was the LFC of GGEP in descending order). The cluster # in the heatmap visualized different reversal strengths of the clusters by comparing each gene’s LFC in the drug's gene expression signatures to the GGEP. Among them, 24 drugs in three clusters (Clusters #1, #3, and #8) exhibited obvious reversal expressions targeting the GGEP. As illustrated in Fig. 3B, the GGEP gene expression could be reversed by the drugs in these three clusters. The expressions of the upregulated genes were reduced, and the downregulated genes were increased. It is noteworthy that the GGEP gene with higher LFCs were more strongly reversely regulated, indicating a high potential in reversing the GGEP. In contrast, the reversal effects of drugs in the rest of four clusters are either negligible or inaccessible due to a considerable number of missing values. Besides, cluster # 8 contains two signatures with a high ratio of missing values (gray column in heatmap), indicating that heatmap is not a reliable tool for candidate prioritization. The clustering results can be found in supplementary file named “supp file 06.csv”.

In addition, we plotted the heatmap at the drug level displaying each gene's median LFC of all gene expression signatures, the result confirmed the potential reversal effect of those 24 drugs (Figure S3). Seventeen of the 24 drugs have undergone clinical trials for GBM treatment, including Cabozantinib [66–68], Axitinib (NCT01508117, NCT01562197, NCT03291314), Mitomycin (NCT01580969, NCT02272270, and NCT02770378) [69], and Simvastatin [70]. Twenty-two of these 24 drugs have a blood–brain barrier (BBB) penetration probability greater than 0.9, which indicates their possible drug delivery to GMB brain tissues. Table 4 lists information of these 24 drugs, including their BBB penetration probabilities, FDA-approved indications, and the number of GBM-related clinical trials they have been tested in.

**Table 4:** Clinical characteristics for the identified DR candidates

Drug Candidates	Cluster ID	BBB	#Clinical Trials	FDA-Approved Indications
Temozolomide	8	0.9879	169	Glioblastoma multiforme, refractory anaplastic astrocytoma
Dasatinib	8, 3	0.507	5	Acute lymphoblastic leukemia; chronic myeloid leukemia
Sirolimus	8, 3	0.9599	5	Lymphangioleiomyomatosis and adults with perivascular epithelioid cell tumors
Etoposide	8	0.9609	5	Testicular and small cell lung tumors
Topotecan	8, 3	0.9659	3	Ovarian cancer, small cell lung cancer, or cervical cancer
Cabozantinib	8	Yes^*^	3	Advanced renal cell carcinoma, hepatocellular carcinoma, and medullary thyroid cancer
Mitomycin	8	0.9659	3	Chemotherapeutic agent for various malignancies
Dacarbazine	8	0.9382	2	Malignant melanoma and Hodgkin's disease
Temsirolimus	8	0.9494	2	Renal cell carcinoma
Bortezomib	1	0.6533	2	Multiple myeloma
Axitinib	8	Yes^*^	2	Advanced renal cell carcinoma
Gemcitabine	8	0.9693	1	Adjunct therapy for ovarian cancer, non-small cell lung carcinoma, metastatic breast cancer, and as a single agent for pancreatic cancer
Cytarabine	8	0.9465	1	Acute non-lymphocytic leukemia, lymphocytic leukemia, and the blast phase of chronic myelocytic leukemia
Romidepsin	8	Yes^*^	1	Cutaneous T-cell lymphoma
Simvastatin	8	0.9422	1^#^	Lower lipid levels and reduce the risk of cardiovascular events
Docetaxel	8	Poor^*^	1^#^	Locally advanced or metastatic breast cancer, metastatic prostate cancer, gastric adenocarcinoma, head and neck cancer
Thalidomide	8	0.9382	1	Newly diagnosed multiple myeloma, erythema nodosum leprosum
Epirubicin hydrochloride	8	0.9951	1^#^	Axillary node metastases in patients of primary breast cancer
Tigecycline	8	0.9836	0	Bacterial infections
Podofilox	8	0.5388	0	External genital warts and perianal warts
Prochlorperazine	8	0.9781	0	Schizophrenia and anxiety and to relieve severe nausea and vomiting
Clofarabine	8	0.9827	0	Relapsed or refractory acute lymphoblastic leukemia
Ciclopirox	8	0.9892	0	Mild to moderate onychomycosis of fingernails and toenails in immunocompetent patients
Tacrolimus	8	0.9659	0	Prevent organ transplant rejection and to treat moderate to severe atopic dermatitis

BBBs are the Blood–Brain Barrier permeability probabilities obtained from the Drugbank database, and * indicates that the BBB were obtained from published studies as they were missing in the Drugbank database. The column of Clinical Trials lists the number of GBM related clinical trials registered in ClinicalTrials.gov, and # indicates that the clinical trials were identified via literature review. The column of Approved Indications lists the drugs' FDA-approved indications obtained from the Drugbank database

### Results on candidates’ reversal strength assessment

**Fig. 4:**
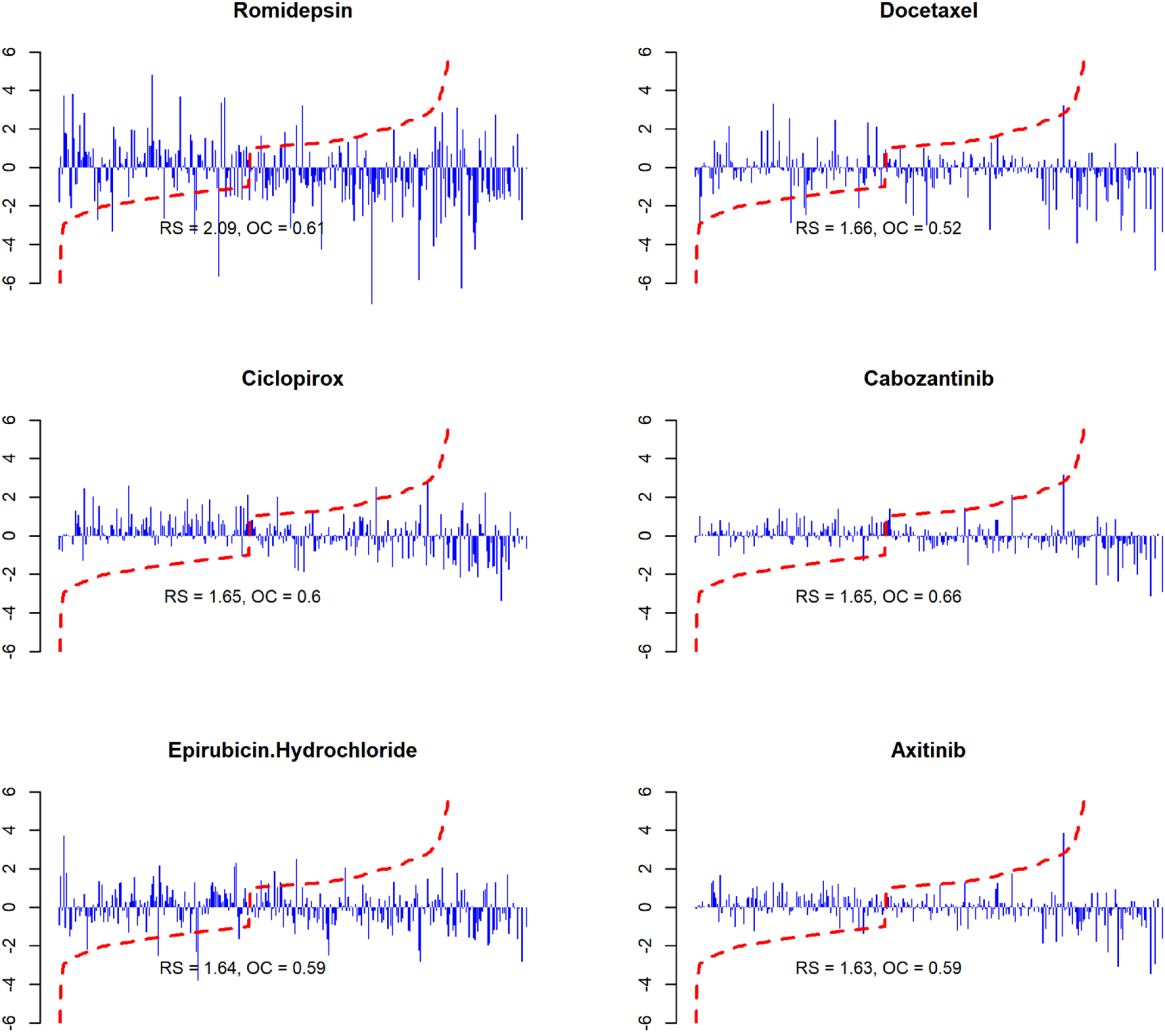
Bar plots of top 6 candidates ranked by the RS. Each bar stands for one gene’s median LFC which were calculated from all identified expression signatures for the drug. The red dotted line stands for the LFCs in GGEP in ascending order. *RS* regulation score, *OC* overall coverage

Based on RS and OC, we evaluated the reversal effect on the candidates. Table 5 lists the top six individual candidates ranked by the calculated RS, which are consistent with their LFC (Fig. 4). The calculated RS and OC and the bar plots for all candidates can be found in the supplementary file named “supp file 07.csv” and “supp file 08.pdf”.

**Table 5:** Top six drug candidates ranked by the RS

Drug	Regulation Score	Overall Coverage	# Clinical Trials
Romidepsin	2.093	0.610	1
Docetaxel	1.664	0.519	1
Ciclopirox	1.653	0.601	0
Cabozantinib	1.652	0.657	1
Epirubicin Hydrochloride	1.641	0.591	1
Axitinib	1.633	0.594	2

Among them, Romidepsin exhibits a significantly higher reversal effect than the others across all indices and from the direct expression of the bar plots. Romidepsin reverses the expression of 61% GGEP genes and its RS, which is a weighted sum of its reversal LFCs targeting these GGEP genes, is 25% higher than the other drugs. An example is Cabozantinib, although it can reverse more GGEP genes than Romidepsin (65.7% vs. 61%), its RS is lower due to smaller reversal LFCs it has. Noteworthy, the results of the signature clustering and the RS evaluation showed high consistency. Specifically, there were 22 candidates (91.6%) presented in both the list of 24 candidates identified by the signature clustering and the list of top 24 candidates ranked by the RS. This suggests that the RS can be applied as an efficient indicator in selecting candidates with top reversal strengths.

We identified five top candidates based on the following criteria: (1) high RS score, (2) not tested in any clinical trials for GBM yet, and (3) high BBB penetration probability. The top five candidates are Ciclopirox, Prochlorperazine, Clofarabine, Tacrolimus, and Tigecycline (Table 6). Some candidates with top RS were excluded because they have undergone clinical trials for GBM, such as, Romidepsin, Cabozantinib, Epirubicin Hydrochloride, and Axitinib, are associated with poor BBB penetration ability [71], or have failed a clinical trial when administered directly [72], like Docetaxel.

**Table 6:** The selected five top candidates

Drug	RS	OC	BBB
Ciclopirox	1.653	0.601	0.9892
Prochlorperazine	1.563	0.623	0.9781
Clofarabine	1.542	0.579	0.9827
Tacrolimus	1.435	0.566	0.9659
Tigecycline	1.302	0.528	0.9836

*RS* regulation score, *OC* overall coverage, *BBB* Blood–Brain Barrier

## Results on drug candidate validation

### Evaluation results with the translator

We evaluated the potential mechanisms of action of these five selected candidates for treating GBM based on scientific evidence collected from the Biomedical Data Translator.

**Fig. 5:**
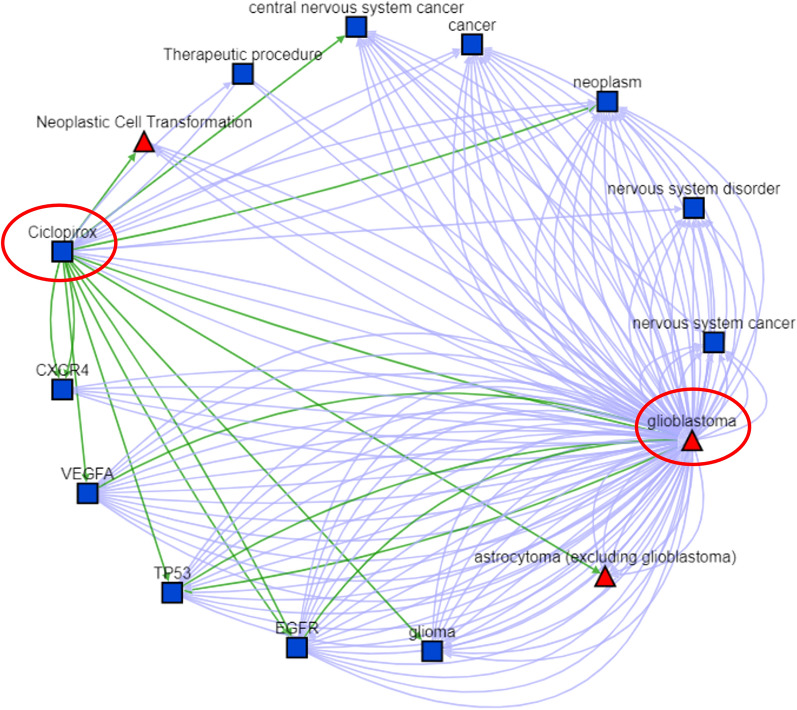
Scientific evidence collected by the Biomedical Data Translator. This network was constructed by possible interactions between Ciclopirox and GBM. We also include indirect interactions connected by another node, such as the VEGFA in this network. The green edges stand for high-confidence associations such as “regulates”, “treats”, “causes”, or “associated with”, while the blue edges stand for low-confidence associations, such as being discussed simultaneously in a study. Please note that direct edges between Ciclopirox and GBM do not always stand for existing studies that GBM has been treated by Ciclopirox

Ciclopirox, an inhibitor of metal-dependent enzymes, was used to treat onychomycosis of fingernails and toenails in immunocompetent patients [73]. The result generated by the Translator is shown in Fig. 5. Detailed evidence can be found in supplementary file named “supp file 09.pdf” or follow the link https://arax.ncats.io/?r=187830. Figure 5 showed that Ciclopirox might impact GBM mechanism via pathways associated with EGFR, VEGFA, TP53, and CXCR4. Subsequent literature review proved that Ciclopirox inhibited the growth of glioblastoma cell lines (U251, SF126, A172, and U118) via simultaneously enhancing JNK/p38 MAPK and NF-κB signaling [74]. Another study showed that Ciclopirox inhibits the proliferation of cancer cell lines including MCF7 breast cancer cells, A549 lung cancer cells, and HT29 colon cancer cells via suppressing Cdc25A [75]. A recent study showed that Ciclopirox could inhibit U-251 GBM cell line via targeting deoxyhypusine hydroxylase [76].

Prochlorperazine is a dopamine D2 receptor antagonists used to treat schizophrenia and anxiety, as well as to relieve severe nausea and vomiting [77]. The search results included in supplementary file named “supp file 10.pdf” (https://arax.ncats.io/?r=187832), from the Translator showed that Prochlorperazine might impact GBM mechanism of neoplastic cell transformation and tumor progression. One publication reported that Prochlorperazine induces concentration-dependent loss in the viability of human glioblastoma cells and its EC50 has been evaluated at the U87-MG cell line [78].

Clofarabine is a DNA polymerase inhibitor used to treat relapsed or refractory acute lymphoblastic leukemia [79]. The evidence, generated by the Translator, is included in the supplementary file named “supp file 11.pdf” (https://arax.ncats.io/?r=233468) and shows that Clofarabine might impact GBM mechanism via pathways associated with STAT3, TP53, apoptosis, and neoplastic cell transformation. Currently Clofarabine is being tested as a repurposing drug to treat CLDN18.2 + solid tumors (NCT05862324) and relapsed solid tumors (NCT02211755). However, its effect on GBM has not been reported yet.

Tacrolimus is an FDA-approved immunosuppressive agent used to prevent organ transplant rejection and to treat moderate to severe atopic dermatitis [80]. The evidence generated by the Translator is included in the supplementary file named “supp file 11.pdf” (https://arax.ncats.io/?r=187831). It shows that Tacrolimus might impact GBM mechanism via pathways associated with EGFR, VEGFA, TP53, and apoptosis. The relevant publication proved that Tacrolimus attenuated the MRP1-mediated chemoresistant phenotype i2 GBM stem-like Cells [81]. Tacrolimus could confer chemosensitivity to anticancer drugs in glioblastoma multiforme cells, offering a possible improvement to the current poor therapy available for high-grade human gliomas [82].

Tigecycline is a Glycylcycline antibiotic used to treat bacterial infections [83]. The Translator results included in the supplementary file named “supp file 13.pdf” (https://arax.ncats.io/?r=187834) shows that Prochlorperazine might impact GBM tumor growth. Similar published results showed that Tigecycline inhibited glioma cell growth in an in vitro study by regulating the miRNA-199b-5p-HES1-AKT pathway [84]. Besides, Tigecycline has demonstrated efficacy in restraining proliferation across various cancer types, including gastric cancer, melanoma, and neuroblastoma [85].

### Therapeutic effects evaluation of top five drug candidates

Based on the systematic assessment of the drug candidates’ reversal strength and evaluation of scientific evidence regarding their mechanism of actions, we considered Ciclopirox, Prochlorperazine, Clofarabine, Tacrolimus, and Tigecycline as the most optimal candidates for *in- vitro* evaluation on GBM cell lines.

#### Concentration response assessment of top five candidates on eight GBM cell lines

For cell viability assay in each glioblastoma cell line, cell seeding density, choice and concentration of positive control, 0.3% DMSO, and incubation times were optimized for assay performance in 1536-well plates. Cells were incubated with 11 concentrations of each drug ranging from 0.56 nM to 33 µM. Data was normalized to cells treated with 0.3% DMSO as 100% viable cells and to 10 µM staurosporine as 0% viable cells. Based on these parameters, the calculated Z-factor of the assay for each cell line was between 0.65–0.82. The IC_50_ values and efficacy of drugs was determined by cell viability assays via a luminescent ATP content readouts.

**Fig. 6:**
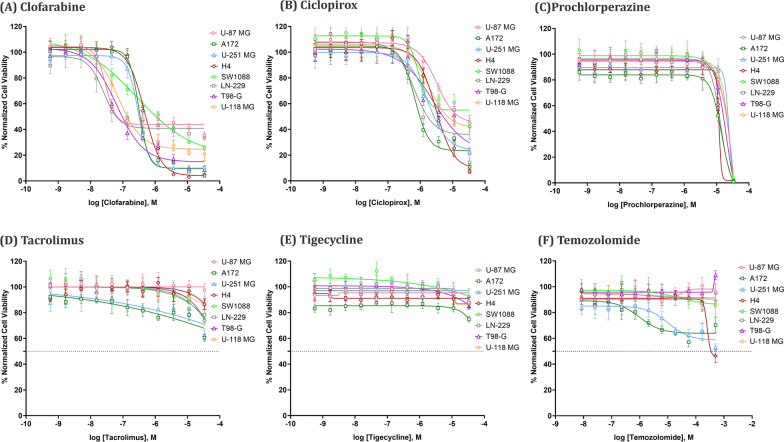
Concentration–response curves of the five drugs. All drugs were tested in eight GBM cell lines in 1536-well plates. Each data point is presented as mean ± SD; n = 8 biological replicates for each condition. Clofarabine and Ciclopirox showed mediate to strong anti-GBM effect while Prochlorperazine, Tacrolimus, and Tigecycline showed little to no efficacy

Out of the five drugs tested, Clofarabine was the most efficacious in killing all glioblastoma cell lines with IC_50_ values ranging from 36.9 nM to 467.5 nM (Fig. 6A and Table 7). Ciclopirox was moderately efficacious, with IC_50_ values between 927.7 nM to 3.2 µM (Fig. 6B and Table 7). TMZ was included for a comparative experiment. Consistent with findings from reported studies [86, 87], TMZ demonstrated high IC_50_ values ranging from 252 µM in H4 cells to approximately 500 µM in U251 cells (Fig. 6F and Table 7). It’s IC_50_ on other GBM cell lines could not be estimated. These results illustrate the superior therapeutic efficacy of Clofarabine and Ciclopirox over TMZ in targeting GBM cells. Prochlorperazine exhibited steep dose–response curves, with estimated IC_50_ values ranging from 12.4 to 19.6 µM (Fig. 6C and Table 7). Due to its relatively high IC_50_ values, Prochlorperazine was not included in subsequent selectivity assessments. Besides, the IC_50_ of Tacrolimus and Tigecycline could not be estimated from their concentration–response curves, demonstrating little to no effect on killing GBM cell lines (Fig. 6D, and E).

**Table 7:** IC_50_ of Candidates

Cell line	IC50			
	Clofarabine	Ciclopirox	Prochlorperazine	Temozolomide
U-87 MG	3.69E-08	3.188E-06	1.27E-05	–
A172	3.148E-07	9.277E-07	1.26E-05	–
U-251 MG	2.997E-07	1.768E-06	1.96E-05	~ 5.0E-4
H4	4.675E-07	2.983E-06	1.33E-05	2.52E-4
SW1088	2.226E-07	1.516E-06	1.59E-05	–
LN-229	3.55E-07	1.616E-06	1.24E-05	–
T98-G	6.475E-08	2.456E-06	1.48E-05	–
U-118 MG	8.027E-08	1.839E-06	1.71E-05	–

##### Selectivity and cell viability assessment of Ciclopirox and Clofarabine

**Fig. 7:**
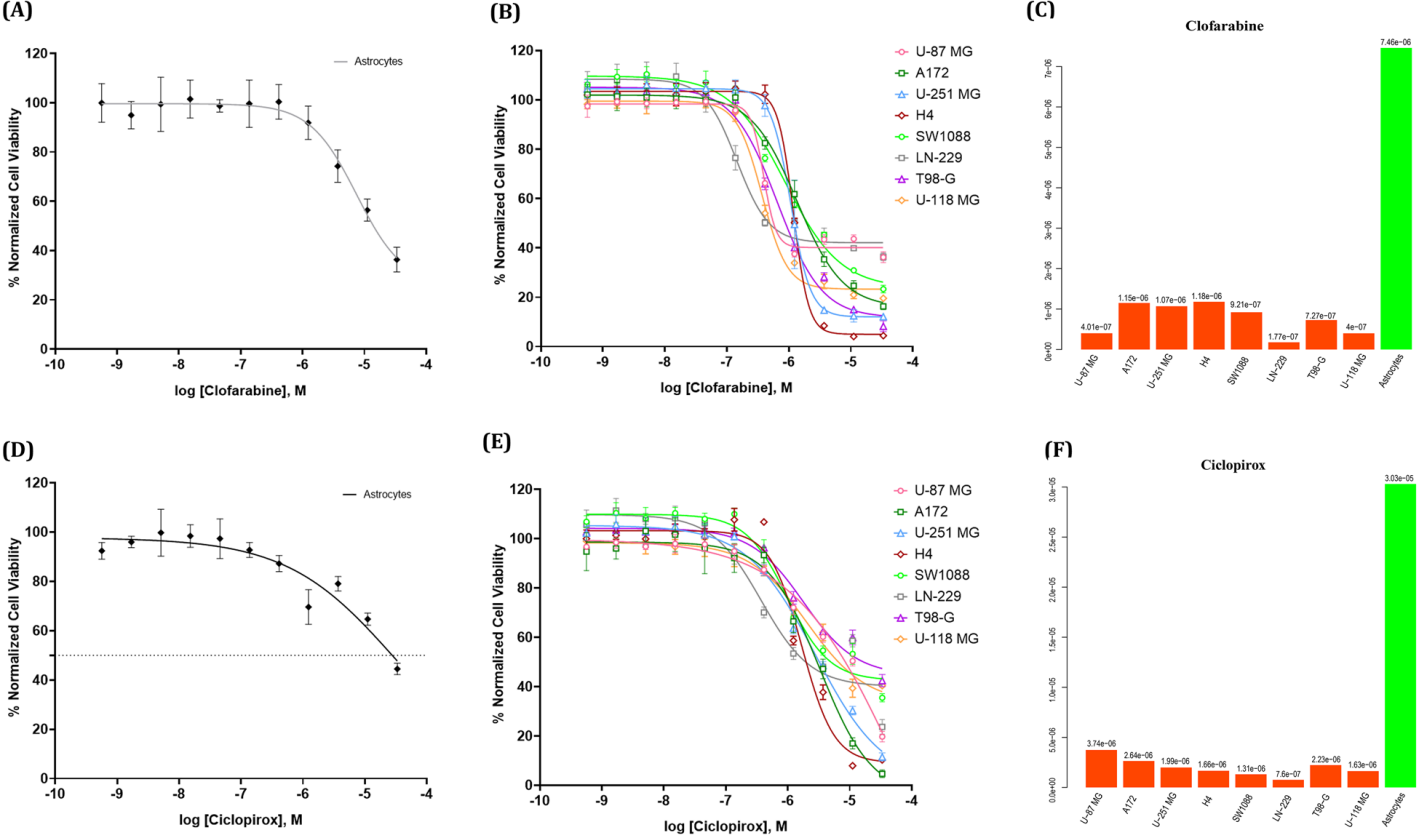
Concentration–response curves of Clofarabine and Ciclopirox in iPSC-derived astrocytes and GBM cells. A: Concentration–response curves of Clofarabine in astrocytes; B: Concentration–response curves of Clofarabine in GBM cells; C: Clofarabine’s IC_50_ on astrocytes (green bar) and GBM cells (red orange bars); D: Concentration–response curves of Ciclopirox in astrocytes; E: Concentration–response curves of Ciclopirox in GBM cells; F: Ciclopirox's IC_50_ on astrocytes (green bar) and GBM cells (red orange bars); In **A**, **B**, **D**, and **E**, Each data point is presented as mean ± SD; n = 4–6 biological replicates for each condition

**Fig. 8:**
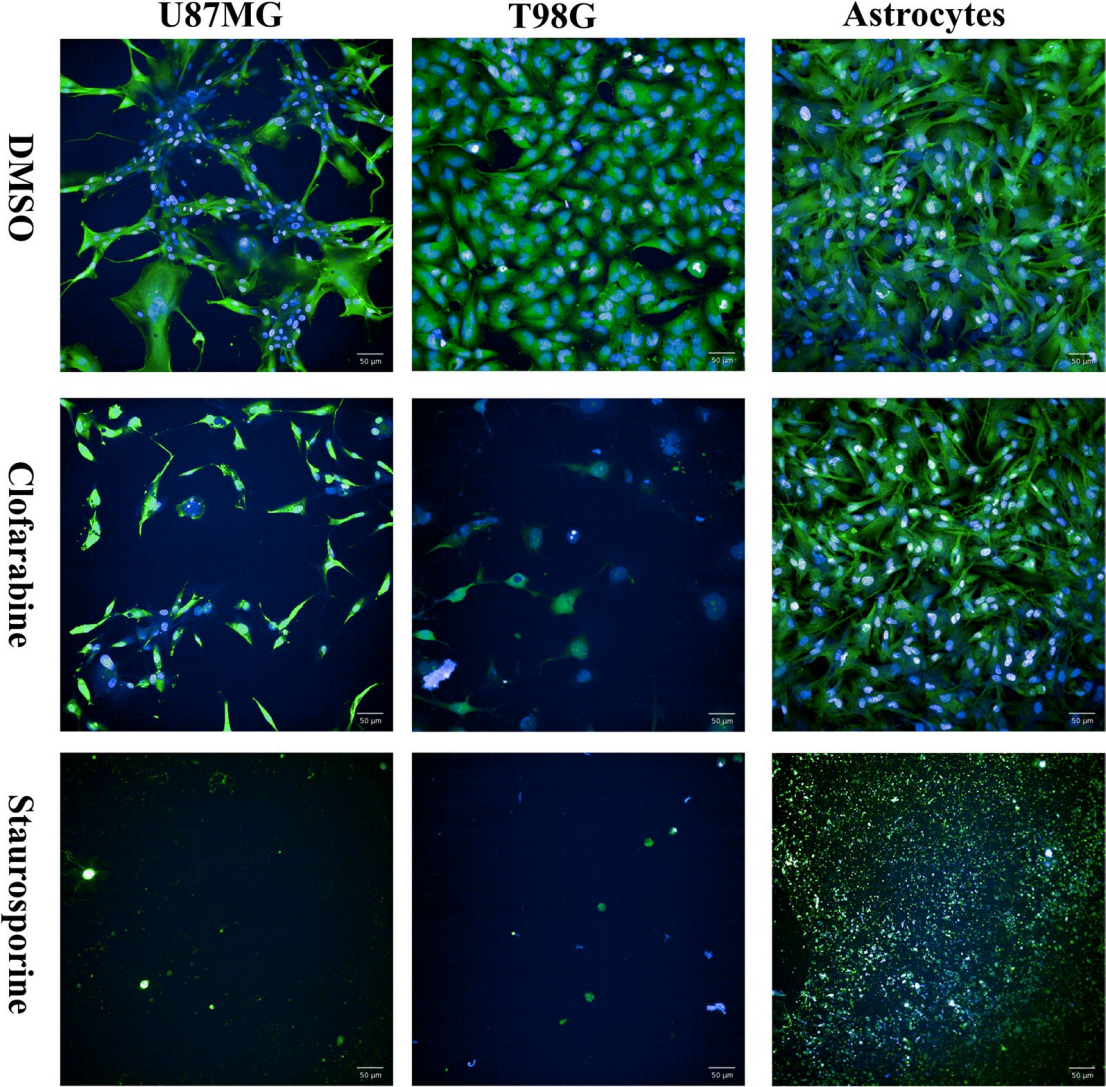
Viability staining GBM and astrocyte cells after Clofarabine (1.2 µM) treatment. Blue color denotes cell nuclei and green denotes plasma membrane of vital cells. DMSO treatment served as a negative control, while 10 µM Staurosporine as a positive control

To assess the efficacy and specificity of Clofarabine and Ciclopirox on GBM cell lines, we then conducted a confirmation assay utilizing both the eight GBM cell lines and an astrocyte cell line as a non-cancerous control. For consistency, all GBM lines and astrocytes were tested in 384-well plates under matching culture conditions. Data was normalized as described above, and the calculated Z-factor of this assay was 0.68. The IC_50_ values for Clofarabine and Ciclopirox in astrocytes was 7.46 nM and 30.03 µM respectively (Fig. 7A and D). In comparison, the IC_50_ values ranged from 177 nM to 1.06 µM for Clofarabine and 760 nM to 3.74 µM for Ciclopirox for the GBM cell lines (Fig. 7B and E). These data indicates that Clofarabine was more efficacious in killing GBM cells compared to astrocytes by a magnitude of 6- to 42-fold (Fig. 7C). In the case of Ciclopirox, GBM cells were 8- to 40-fold more susceptible than astrocytes to the drug (Fig. 7F). The results showed that both drugs had high specificity targeting GBM cell lines, their therapeutic effect on GBM warrants further investigation. Figure 8 shows representative images of the difference in viabilities of two GBM cell lines and astrocytes when treated with 1.2 µM Clofarabine. At this concentration, Clofarabine at this concentration can kill GBM cells, while it has minimal effect on astrocytes. The staining images of all GBM and astrocyte cell lines treated with Clofarabine and Ciclopirox at 1.2 µM are provided in the supplementary file named “supp file 14.pdf”. The staining images at other concentrations are available upon request.

## Discussion

The development of pharmaceutical interventions for rare diseases are challenged by low prevalence. Among them, GBM remains a devastating rare disease with limited treatment options and a short life expectancy. To fill the gap, in this study, we introduced a novel computational drug repurposing approach for GBM with consideration of the concept of reversal gene expression by performing multi-omics data analysis and *in-vitro* experiments. To this end, we successfully identified two promising drug candidates, Clofarabine and Ciclopirox for GBM, for further investigation.

In this study, we collected 328 transcriptome and 3 proteome data sets of GBM patients from a public database and a published study. Subsequently, we constructed the GGEP based on 318 DEGs resulting from multi-omics analysis. This GGEP proved to be an effective profile in identifying DR candidates. However, the data type and sample size we used were limited due to the limited existing studies. When possible, the inclusion of more data types, such as whole genome sequencing data, metabolism data, and clinical data would produce deepened insight in GBM mechanisms and possibly more promising drug repurposing candidates.

We utilized two self-defined indices, RS, and OC to quantify DR candidates’ reversal strength. The results showed that RS and OC can effectively prioritize candidates, resulting in promising candidates that were validated by *in-vitro* experiments. Five of the top six candidates ranked by RS (Table 5) are currently in clinical trials for GBM. The sixth candidate, Ciclopirox, exhibited promising in vitro efficacy in this study. These two indices were calculated by comparing the averaged LFCs in drug expression signatures with those in GGEP. Inclusion of more features, such as drug concentrations and treatment time will improve the prioritization. Furthermore, these indices focus on individual drugs and cannot be directly applied on the prediction of combination therapies. The next step in our investigation is to expand the prioritization methods to reflect more aspects of the candidates’ characteristics, such as toxicity, adverse effects, and drug-drug interactions. This will increase the robustness of the final candidate selection, especially for the combination therapies.

Through the in-vitro experiments, we identified Clofarabine and Ciclopirox as two promising repurposing drug candidates for GBM, which are further proved by the existing studies. Ciclopirox has been repurposed to treat breast cancer, lung cancer cells, and colon cancer, it has demonstrated inhibitory effect on GBM cell lines [74, 75]. Similarly, Clofarabine is being tested as a repurposing drug to treat solid tumors (NCT05862324 and NCT02211755). Future work will include in vivo studies to confirm their therapeutic efficacy in GBM models, followed by the design of clinical trials for candidates that could successfully pass preclinical testing. Additionally, we examined the therapeutic potential of combination therapies. Using the RS and OC scores, we ranked the combinations of top-ranked candidate drugs. We have planned in vitro experiments to evaluate whether these drug combinations exhibit synergistic or additive effects on GBM cell viability, while simultaneously investigating the molecular mechanisms underlying these interactions. The methods and results of these studies will be reported in a separate publication.

In this study, we identified one psychotropic drug, Prochlorperazine as an effective drug candidate in inhibiting GBM cell lines. Several studies have reported psychotropic drugs as potential anti-GBM agents given their ability to penetrate the BBB and modulate neurotransmitter levels in the brain [91–95]. It is worthy to note that a small number of psychotropic drugs identified from this study, which might be due to the minimal overlap between GGEP and psychotropic drug perturbation signatures in iLINCS. Applying the RS and OC indices to additional signature databases could potentially uncover more promising psychotropic drug candidates for GBM repurposing.

In this preliminary study, the iLINCS database was chosen for its perturbation signature comparison function, to validate predictive power of RS and OC. For the next step, we will integrate more perturbation signature databases, such as Cancer Cell Line Encyclopedia (CCLE) [88], Genomics of Drug Sensitivity in Cancer (GDSC) [89], ChemPert [96], and PerturBase [97] to identify more potential drug candidates with stronger therapeutic effect. Furthermore, at the time of performing this study, there was no existing multi-omics database designed specifically for rare diseases, therefore we manually collected the omics data sets from various sources after laborious searching and reviewing. Thus, it concludes that a rare disease-based omics data repository would greatly speed up the pace of DR in rare diseases, as well as various translational studies employing advanced artificial intelligence (AI) tools.

## Supplementary Information

Additional file 1

Additional file 2

Additional file 3

Additional file 4

Additional file 5

Additional file 6

Additional file 7

Additional file 8

Additional file 9

Additional file 10

Additional file 11

Additional file 12

Additional file 13

Additional file 14

Additional file 15

Additional file 16

Additional file 17

## Data Availability

The transcriptomics datasets analyzed in this study are publicly available from the Chinese Glioma Genome Atlas (CGGA) and can be accessed at https://www.cgga.org.cn/, as originally described in Zhao et al. [19] 10.1016/j.gpb.2020.10.005. The proteomics dataset re-analyzed in this study was previously published by Buser et al. [20] in EBioMedicine. The R code used for this project is publicly available at https://github.com/ncats/drug_rep/tree/sx-rep-branch/omics-based%20GBM%20rep.
