# Supplementary material for: Reversal gene expression assessment for drug repurposing, a case study of glioblastoma
Source: J Transl Med. 2025 Jan 7;23:25. doi: 10.1186/s12967-024-06046-1 (PMC11706105; doi:10.1186/s12967-024-06046-1)
Supplement: Supplementary file 1 — Additional file 1 [file 12967_2024_6046_MOESM1_ESM.pdf]

ARAX User Interface

Documentation: Overview TRAPI 1.4.2 Resources

Input

Queries

Settings

List A 0

List B 0

Compare Lists

History 2

Output

Summary

Provenance

Knowledge Graph

Results 17

Messages 1329

Tools

Synonyms

Dev Info

System Activity

SmartAPI Info

Translator Testing

Reset All

Late Wildfowl

KNOWLEDGE GRAPH

↻
B
F
C
R

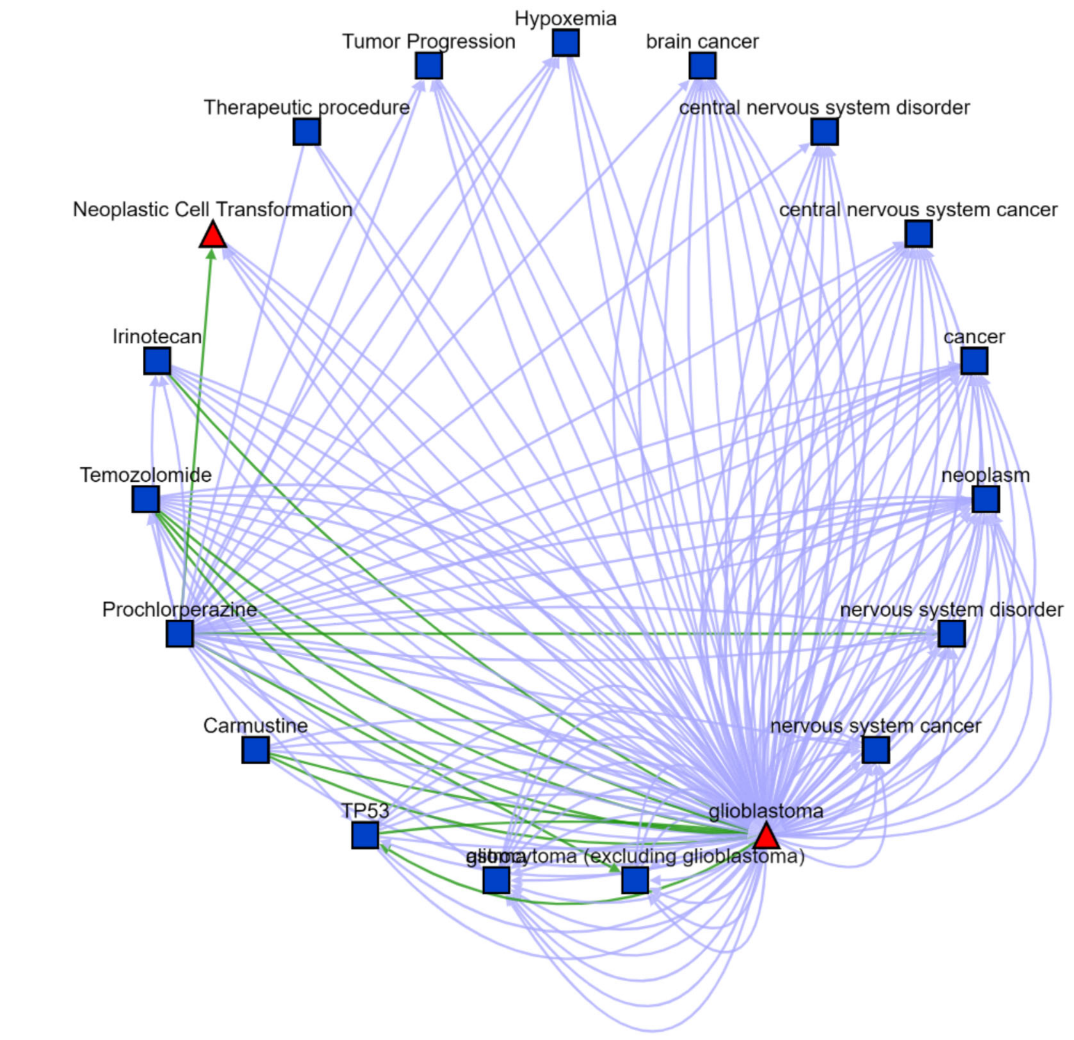

All nodes and edges:

HP:0012418 *Hypoxemia*

biolink:affects ➔ MONDO:0018177

biolink:coexists_with ➔ MONDO:0018177

biolink:occurs_together_in_literature_with ➔ MONDO:0018177

Input
Queries
Settings
List A 0
List B 0
Compare Lists
History 2

Output
Summary
Provenance
Knowledge Graph
Results 17
Messages 1329

Tools
Synonyms
Dev Info
System Activity
SmartAPI Info
Translator Testing
Reset All

Late Wildfowl

← biolink:positively_correlated_with PUBCHEM.COMPOUND:4917

MONDO:0001657 *brain cancer*

biolink:affects →	MONDO:0018177
biolink:causes →	MONDO:0018177
biolink:coexists_with →	MONDO:0018177
biolink:occurs_together_in_literature_with →	MONDO:0018177
biolink:occurs_together_in_literature_with →	MONDO:0018177
biolink:occurs_together_in_literature_with →	MONDO:0018177
biolink:occurs_together_in_literature_with →	MONDO:0018177
← biolink:positively_correlated_with	PUBCHEM.COMPOUND:4917
← biolink:coexists_with	MONDO:0018177
← biolink:subclass_of	MONDO:0018177
← biolink:subclass_of	MONDO:0018177

MONDO:0002602 *central nervous system disorder*

biolink:occurs_together_in_literature_with →	MONDO:0018177
← biolink:subclass_of	MONDO:0018177
← biolink:subclass_of	MONDO:0018177
← biolink:subclass_of	MONDO:0018177
← biolink:subclass_of	MONDO:0018177
← biolink:subclass_of	MONDO:0018177
← biolink:positively_correlated_with	PUBCHEM.COMPOUND:4917

MONDO:0002714 *central nervous system cancer*

biolink:coexists_with →	MONDO:0018177
biolink:occurs_together_in_literature_with →	MONDO:0018177
biolink:occurs_together_in_literature_with →	MONDO:0018177
biolink:occurs_together_in_literature_with →	MONDO:0018177
← biolink:subclass_of	MONDO:0018177
← biolink:subclass_of	MONDO:0018177
← biolink:subclass_of	MONDO:0018177
← biolink:subclass_of	MONDO:0018177
← biolink:subclass_of	MONDO:0018177
← biolink:subclass_of	MONDO:0018177
← biolink:positively_correlated_with	PUBCHEM.COMPOUND:4917
← biolink:subclass_of	MONDO:0018177

MONDO:0004992 *cancer*

biolink:affects →	MONDO:0018177
biolink:causes →	MONDO:0018177
biolink:coexists_with →	MONDO:0018177
biolink:occurs_together_in_literature_with →	MONDO:0018177
biolink:occurs_together_in_literature_with →	MONDO:0018177

ARAX User Interface

Documentation: Overview TRAPI 1.4.2 Resources

Input

Queries

Settings

List A 0

List B 0

Compare Lists

History 2

Output

Summary

Provenance

Knowledge Graph

Results 17

Messages 1329

Tools

Synonyms

Dev Info

System Activity

SmartAPI Info

Translator Testing

Reset All

Late Wildfowl

	[illegible]	[illegible]
	← biolink:subclass_of	MONDO:0018177
	← biolink:treats	PUBCHEM.COMPOUND:4917
	← biolink:subclass_of	MONDO:0018177
	← biolink:positively_correlated_with	PUBCHEM.COMPOUND:4917
	← biolink:affects	MONDO:0018177
	← biolink:coexists_with	MONDO:0018177
	← biolink:subclass_of	MONDO:0018177
	← biolink:occurs_together_in_literature_with	PUBCHEM.COMPOUND:4917

**MONDO:0005070**	*neoplasm*	
	biolink:causes →	MONDO:0018177
	biolink:occurs_together_in_literature_with →	MONDO:0018177
	biolink:occurs_together_in_literature_with →	MONDO:0018177
	biolink:occurs_together_in_literature_with →	MONDO:0018177
	biolink:occurs_together_in_literature_with →	MONDO:0018177
	← biolink:subclass_of	MONDO:0018177
	← biolink:subclass_of	MONDO:0018177
	← biolink:subclass_of	MONDO:0018177
	← biolink:subclass_of	MONDO:0018177
	← biolink:treats	PUBCHEM.COMPOUND:4917
	← biolink:subclass_of	MONDO:0018177
	← biolink:affects	MONDO:0018177
	← biolink:causes	MONDO:0018177
	← biolink:coexists_with	MONDO:0018177
	← biolink:manifestation_of	MONDO:0018177
	← biolink:subclass_of	MONDO:0018177
	← biolink:occurs_together_in_literature_with	PUBCHEM.COMPOUND:4917
	← biolink:occurs_together_in_literature_with	PUBCHEM.COMPOUND:4917

**MONDO:0005071**	*nervous system disorder*	
	biolink:occurs_together_in_literature_with →	MONDO:0018177
	← biolink:subclass_of	MONDO:0018177
	← biolink:subclass_of	MONDO:0018177
	← biolink:subclass_of	MONDO:0018177
	← biolink:subclass_of	MONDO:0018177
	← biolink:treats	PUBCHEM.COMPOUND:4917
	← biolink:subclass_of	MONDO:0018177
	← biolink:associated_with [q]	PUBCHEM.COMPOUND:4917
	← biolink:subclass_of	MONDO:0018177
	← biolink:occurs_together_in_literature_with	PUBCHEM.COMPOUND:4917

**MONDO:0005872**	*nervous system cancer*	
	biolink:occurs_together_in_literature_with →	MONDO:0018177

	[illegible]	[illegible]
	← biolink:subclass_of	MONDO:0018177
	← biolink:subclass_of	MONDO:0018177
	← biolink:positively_correlated_with	PUBCHEM.COMPOUND:4917
	← biolink:subclass_of	MONDO:0018177

**MONDO:0018177**	*glioblastoma*	
	biolink:subclass_of →	MONDO:0002602
	biolink:subclass_of →	MONDO:0002714
	biolink:subclass_of →	MONDO:0004992
	biolink:subclass_of →	MONDO:0005070
	biolink:subclass_of →	MONDO:0005071
	biolink:subclass_of →	MONDO:0005872
	biolink:subclass_of →	MONDO:0021042
	biolink:subclass_of →	MONDO:0002602
	biolink:subclass_of →	MONDO:0002714
	biolink:subclass_of →	MONDO:0004992
	biolink:subclass_of →	MONDO:0005070
	biolink:subclass_of →	MONDO:0005071
	biolink:subclass_of →	MONDO:0005872
	biolink:subclass_of →	MONDO:0021042
	biolink:subclass_of →	MONDO:0002714
	biolink:subclass_of →	MONDO:0002602
	biolink:subclass_of →	MONDO:0002714
	biolink:subclass_of →	MONDO:0004992
	biolink:subclass_of →	MONDO:0005070
	biolink:subclass_of →	MONDO:0005071
	biolink:subclass_of →	MONDO:0005872
	biolink:subclass_of →	MONDO:0021042
	biolink:subclass_of →	MONDO:0002602
	biolink:subclass_of →	MONDO:0002714
	biolink:subclass_of →	MONDO:0004992
	biolink:subclass_of →	MONDO:0005070
	biolink:subclass_of →	MONDO:0005071
	biolink:subclass_of →	MONDO:0005872
	biolink:subclass_of →	MONDO:0021042
	biolink:subclass_of →	MONDO:0002602
	biolink:subclass_of →	MONDO:0002714
	biolink:subclass_of →	MONDO:0004992
	biolink:subclass_of →	MONDO:0005070
	biolink:subclass_of →	MONDO:0005071
	biolink:subclass_of →	MONDO:0005872
	biolink:subclass_of →	MONDO:0021042
	biolink:has_participant [q] →	NCBIGene:7157

ARAX User Interface

Documentation: Overview TRAPI 1.4.2 Resources

Input

Queries

Settings

List A 0

List B 0

Compare Lists

History 2

Output

Summary

Provenance

Knowledge Graph

Results 17

Messages 1329

Tools

Synonyms

Dev Info

System Activity

SmartAPI Info

Translator Testing

Reset All

Late Wildfowl

[illegible]	[illegible]
biolink:affects →	UMLS:C0178874
biolink:causes →	MONDO:0005070
biolink:causes →	UMLS:C0178874
biolink:coexists_with →	MONDO:0001657
biolink:coexists_with →	MONDO:0004992
biolink:coexists_with →	MONDO:0005070
biolink:coexists_with →	MONDO:0019781
biolink:coexists_with →	MONDO:0021042
biolink:coexists_with →	UMLS:C0007621
biolink:has_part →	NCBIGene:7157
biolink:manifestation_of →	MONDO:0005070
biolink:subclass_of →	MONDO:0001657
biolink:subclass_of →	MONDO:0001657
biolink:subclass_of →	MONDO:0002714
biolink:subclass_of →	MONDO:0004992
biolink:subclass_of →	MONDO:0005070
biolink:subclass_of →	MONDO:0005071
biolink:subclass_of →	MONDO:0005872
biolink:subclass_of →	MONDO:0019781
biolink:subclass_of →	MONDO:0019781
biolink:subclass_of →	MONDO:0019781
biolink:subclass_of →	MONDO:0019781
biolink:subclass_of →	MONDO:0021042
biolink:subclass_of →	MONDO:0021042
biolink:subclass_of →	MONDO:0021042
← biolink:ameliorates	PUBCHEM.COMPOUND:2578
← biolink:ameliorates	PUBCHEM.COMPOUND:5394
← biolink:ameliorates	PUBCHEM.COMPOUND:60838
← biolink:has_adverse_event	PUBCHEM.COMPOUND:5394
← biolink:genetic_association	NCBIGene:7157
← biolink:genetic_association	NCBIGene:7157
← biolink:treats	PUBCHEM.COMPOUND:4917
← biolink:treats [q]	PUBCHEM.COMPOUND:2578
← biolink:treats [q]	PUBCHEM.COMPOUND:2578
← biolink:treats [q]	PUBCHEM.COMPOUND:5394
← biolink:treats [q]	PUBCHEM.COMPOUND:5394
← biolink:treats [q]	PUBCHEM.COMPOUND:60838
← biolink:affects	HP:0012418
← biolink:coexists_with	HP:0012418
← biolink:affects	MONDO:0001657
← biolink:causes	MONDO:0001657
← biolink:coexists_with	MONDO:0001657
← biolink:coexists_with	MONDO:0002714

← biolink:coexists_with	[illegible]
← biolink:causes	MONDO:0005070
← biolink:coexists_with	MONDO:0019781
← biolink:coexists_with	MONDO:0021042
← biolink:affects	NCBIGene:7157
← biolink:affects [q]	NCBIGene:7157
← biolink:causes	NCBIGene:7157
← biolink:gene_associated_with_condition	NCBIGene:7157
← biolink:gene_associated_with_condition	NCBIGene:7157
← biolink:related_to	NCBIGene:7157
← biolink:treats	PUBCHEM.COMPOUND:2578
← biolink:treats	PUBCHEM.COMPOUND:2578
← biolink:affects [q]	PUBCHEM.COMPOUND:5394
← biolink:causes	PUBCHEM.COMPOUND:5394
← biolink:disrupts	PUBCHEM.COMPOUND:5394
← biolink:related_to	PUBCHEM.COMPOUND:5394
← biolink:treats	PUBCHEM.COMPOUND:5394
← biolink:treats	PUBCHEM.COMPOUND:5394
← biolink:treats	PUBCHEM.COMPOUND:5394
← biolink:related_to	PUBCHEM.COMPOUND:60838
← biolink:treats	PUBCHEM.COMPOUND:60838
← biolink:treats	PUBCHEM.COMPOUND:60838
← biolink:causes	UMLS:C0007621
← biolink:coexists_with	UMLS:C0007621
← biolink:prevents	UMLS:C0087111
← biolink:treats	UMLS:C0087111
← biolink:causes	UMLS:C0178874
← biolink:coexists_with	UMLS:C0178874
← biolink:has_adverse_event	PUBCHEM.COMPOUND:4917
← biolink:treats	PUBCHEM.COMPOUND:4917
← biolink:treats	PUBCHEM.COMPOUND:4917
← biolink:occurs_together_in_literature_with	PUBCHEM.COMPOUND:2578
← biolink:occurs_together_in_literature_with	PUBCHEM.COMPOUND:4917
← biolink:occurs_together_in_literature_with	PUBCHEM.COMPOUND:60838
← biolink:occurs_together_in_literature_with	PUBCHEM.COMPOUND:5394
← biolink:occurs_together_in_literature_with	MONDO:0005070
← biolink:occurs_together_in_literature_with	MONDO:0005070
← biolink:occurs_together_in_literature_with	MONDO:0019781
← biolink:occurs_together_in_literature_with	MONDO:0021042
← biolink:occurs_together_in_literature_with	MONDO:0021042
← biolink:occurs_together_in_literature_with	HP:0012418
← biolink:occurs_together_in_literature_with	MONDO:0001657
← biolink:occurs_together_in_literature_with	MONDO:0001657
← biolink:occurs_together_in_literature_with	MONDO:0002714

← biolink:occurs_together_in_literature_with	[illegible]
← biolink:occurs_together_in_literature_with	MONDO:0002602
← biolink:occurs_together_in_literature_with	MONDO:0002714
← biolink:occurs_together_in_literature_with	MONDO:0004992
← biolink:occurs_together_in_literature_with	MONDO:0004992
← biolink:occurs_together_in_literature_with	MONDO:0005070
← biolink:occurs_together_in_literature_with	MONDO:0005070
← biolink:occurs_together_in_literature_with	MONDO:0005071
← biolink:occurs_together_in_literature_with	MONDO:0005872
← biolink:occurs_together_in_literature_with	MONDO:0019781
← biolink:occurs_together_in_literature_with	MONDO:0021042
← biolink:occurs_together_in_literature_with	MONDO:0021042

MONDO:0019781 *astrocytoma (excluding glioblastoma)*

biolink:coexists_with →	MONDO:0018177
biolink:occurs_together_in_literature_with →	MONDO:0018177
biolink:occurs_together_in_literature_with →	MONDO:0018177
← biolink:associated_with [q]	PUBCHEM.COMPOUND:4917
← biolink:coexists_with	MONDO:0018177
← biolink:subclass_of	MONDO:0018177
← biolink:subclass_of	MONDO:0018177
← biolink:subclass_of	MONDO:0018177
← biolink:subclass_of	MONDO:0018177

MONDO:0021042 *glioma*

biolink:coexists_with →	MONDO:0018177
biolink:occurs_together_in_literature_with →	MONDO:0018177
biolink:occurs_together_in_literature_with →	MONDO:0018177
biolink:occurs_together_in_literature_with →	MONDO:0018177
biolink:occurs_together_in_literature_with →	MONDO:0018177
← biolink:subclass_of	MONDO:0018177
← biolink:subclass_of	MONDO:0018177
← biolink:subclass_of	MONDO:0018177
← biolink:subclass_of	MONDO:0018177
← biolink:treats	PUBCHEM.COMPOUND:4917
← biolink:subclass_of	MONDO:0018177
← biolink:coexists_with	MONDO:0018177
← biolink:subclass_of	MONDO:0018177
← biolink:subclass_of	MONDO:0018177
← biolink:subclass_of	MONDO:0018177

NCBIGene:7157 *TP53*

biolink:genetic_association →	MONDO:0018177
biolink:genetic_association →	MONDO:0018177

Input

Queries

Settings

List A 0

List B 0

Compare Lists

History 2

Output

Summary

Provenance

Knowledge Graph

Results 17

Messages 1329

Tools

Synonyms

Dev Info

System Activity

SmartAPI Info

Translator Testing

Reset All

Late Wildfowl

biolink:causes →	[illegible]
biolink:gene_associated_with_condition →	MONDO:0018177
biolink:gene_associated_with_condition →	MONDO:0018177
biolink:related_to →	MONDO:0018177
← biolink:has_participant [q]	MONDO:0018177
← biolink:has_part	MONDO:0018177
← biolink:affects	PUBCHEM.COMPOUND:4917

PUBCHEM.COMPOUND:2578 *Carmustine*

biolink:ameliorates →	MONDO:0018177
biolink:treats [q] →	MONDO:0018177
biolink:treats [q] →	MONDO:0018177
biolink:treats →	MONDO:0018177
biolink:treats →	MONDO:0018177
biolink:occurs_together_in_literature_with →	MONDO:0018177
← biolink:occurs_together_in_literature_with	PUBCHEM.COMPOUND:4917

PUBCHEM.COMPOUND:4917 *Prochlorperazine*

biolink:has_adverse_event →	HP:0012418
biolink:has_adverse_event →	UMLS:C0178874
biolink:has_adverse_event →	HP:0012418
biolink:has_adverse_event →	UMLS:C0178874
biolink:treats →	MONDO:0004992
biolink:treats →	MONDO:0005070
biolink:treats →	MONDO:0005071
biolink:treats →	MONDO:0018177
biolink:treats →	MONDO:0021042
biolink:positively_correlated_with →	HP:0012418
biolink:positively_correlated_with →	MONDO:0001657
biolink:positively_correlated_with →	MONDO:0002602
biolink:positively_correlated_with →	MONDO:0002714
biolink:positively_correlated_with →	MONDO:0004992
biolink:positively_correlated_with →	MONDO:0005872
biolink:positively_correlated_with →	PUBCHEM.COMPOUND:5394
biolink:positively_correlated_with →	PUBCHEM.COMPOUND:60838
biolink:associated_with [q] →	MONDO:0005071
biolink:associated_with [q] →	MONDO:0019781
biolink:associated_with [q] →	UMLS:C0007621
biolink:affects →	NCBIGene:7157
biolink:has_adverse_event →	MONDO:0018177
biolink:treats →	MONDO:0018177
biolink:treats →	MONDO:0018177
biolink:occurs_together_in_literature_with →	PUBCHEM.COMPOUND:2578
biolink:occurs_together_in_literature_with →	PUBCHEM.COMPOUND:60838

biolink:occurs_together_in_literature_with →	[illegible]
biolink:occurs_together_in_literature_with →	MONDO:0005071
biolink:occurs_together_in_literature_with →	MONDO:0018177
← biolink:has_input	UMLS:C0087111

PUBCHEM.COMPOUND:5394 *Temozolomide*

biolink:ameliorates →	MONDO:0018177
biolink:has_adverse_event →	MONDO:0018177
biolink:treats [q] →	MONDO:0018177
biolink:treats [q] →	MONDO:0018177
biolink:affects [q] →	MONDO:0018177
biolink:causes →	MONDO:0018177
biolink:disrupts →	MONDO:0018177
biolink:related_to →	MONDO:0018177
biolink:treats →	MONDO:0018177
biolink:treats →	MONDO:0018177
biolink:treats →	MONDO:0018177
biolink:occurs_together_in_literature_with →	MONDO:0018177
← biolink:positively_correlated_with	PUBCHEM.COMPOUND:4917

PUBCHEM.COMPOUND:60838 *Irinotecan*

biolink:ameliorates →	MONDO:0018177
biolink:treats [q] →	MONDO:0018177
biolink:related_to →	MONDO:0018177
biolink:treats →	MONDO:0018177
biolink:treats →	MONDO:0018177
biolink:occurs_together_in_literature_with →	MONDO:0018177
← biolink:positively_correlated_with	PUBCHEM.COMPOUND:4917
← biolink:occurs_together_in_literature_with	PUBCHEM.COMPOUND:4917

UMLS:C0007621 *Neoplastic Cell Transformation*

biolink:causes →	MONDO:0018177
biolink:coexists_with →	MONDO:0018177
← biolink:associated_with [q]	PUBCHEM.COMPOUND:4917
← biolink:affects	MONDO:0018177
← biolink:coexists_with	MONDO:0018177

UMLS:C0087111 *Therapeutic procedure*

biolink:has_input →	PUBCHEM.COMPOUND:4917
biolink:prevents →	MONDO:0018177
biolink:treats →	MONDO:0018177

UMLS:C0178874 *Tumor Progression*

biolink:causes →	MONDO:0018177

ARAX User Interface

Documentation: Overview TRAPI 1.4.2 Resources

Input

Queries

Settings

List A 0

List B 0

Compare Lists

History 2

Output

Summary

Provenance

Knowledge Graph

Results 17

Messages 1329

Tools

Synonyms

Dev Info

System Activity

SmartAPI Info

Translator Testing

Reset All

Late Wildfowl

ARAX User Interface

Documentation: Overview TRAPI 1.4.2 Resources

Input

Queries

Settings

List A 0

List B 0

Compare Lists

History 2

Output

Summary

Provenance

Knowledge Graph

Results 17

Messages 1329

Tools

Synonyms

Dev Info

System Activity

SmartAPI Info

Translator Testing

Reset All

Late Wildfowl

ARAX User Interface

Documentation: Overview TRAPI 1.4.2 Resources

Input

Queries

Settings

List A 0

List B 0

Compare Lists

History 2

Output

Summary

Provenance

Knowledge Graph

Results 17

Messages 1329

Tools

Synonyms

Dev Info

System Activity

SmartAPI Info

Translator Testing

Reset All

Late Wildfowl

ARAX User Interface

Documentation: Overview TRAPI 1.4.2 Resources

Input

Queries

Settings

List A 0

List B 0

Compare Lists

History 2

Output

Summary

Provenance

Knowledge Graph

Results 17

Messages 1329

Tools

Synonyms

Dev Info

System Activity

SmartAPI Info

Translator Testing

Reset All

Late Wildfowl

ARAX User Interface

Documentation: Overview TRAPI 1.4.2 Resources

Input

Queries

Settings

List A 0

List B 0

Compare Lists

History 2

Output

Summary

Provenance

Knowledge Graph

Results 17

Messages 1329

Tools

Synonyms

Dev Info

System Activity

SmartAPI Info

Translator Testing

Reset All

Late Wildfowl

ARAX User Interface

Documentation: Overview TRAPI 1.4.2 Resources

Input

Queries

Settings

List A 0

List B 0

Compare Lists

History 2

Output

Summary

Provenance

Knowledge Graph

Results 17

Messages 1329

Tools

Synonyms

Dev Info

System Activity

SmartAPI Info

Translator Testing

Reset All

Late Wildfowl

ARAX User Interface

Documentation: Overview TRAPI 1.4.2 Resources

Input

Queries

Settings

List A 0

List B 0

Compare Lists

History 2

Output

Summary

Provenance

Knowledge Graph

Results 17

Messages 1329

Tools

Synonyms

Dev Info

System Activity

SmartAPI Info

Translator Testing

Reset All

Late Wildfowl
