# Supplementary material for: Reversal gene expression assessment for drug repurposing, a case study of glioblastoma
Source: J Transl Med. 2025 Jan 7;23:25. doi: 10.1186/s12967-024-06046-1 (PMC11706105; doi:10.1186/s12967-024-06046-1)
Supplement: Supplementary file 2 — Additional file 2 [file 12967_2024_6046_MOESM2_ESM.pdf]

ARAX User Interface

Documentation: Overview TRAPI 1.4.2 Resources

Input

Queries

Settings

List A 0

List B 0

Compare Lists

History 1

Output

Summary

Provenance

Knowledge Graph

Results 15

Messages 1671

Tools

Synonyms

Dev Info

System Activity

SmartAPI Info

Translator Testing

Reset All

Late Wildfowl

KNOWLEDGE GRAPH

↻ B F C R

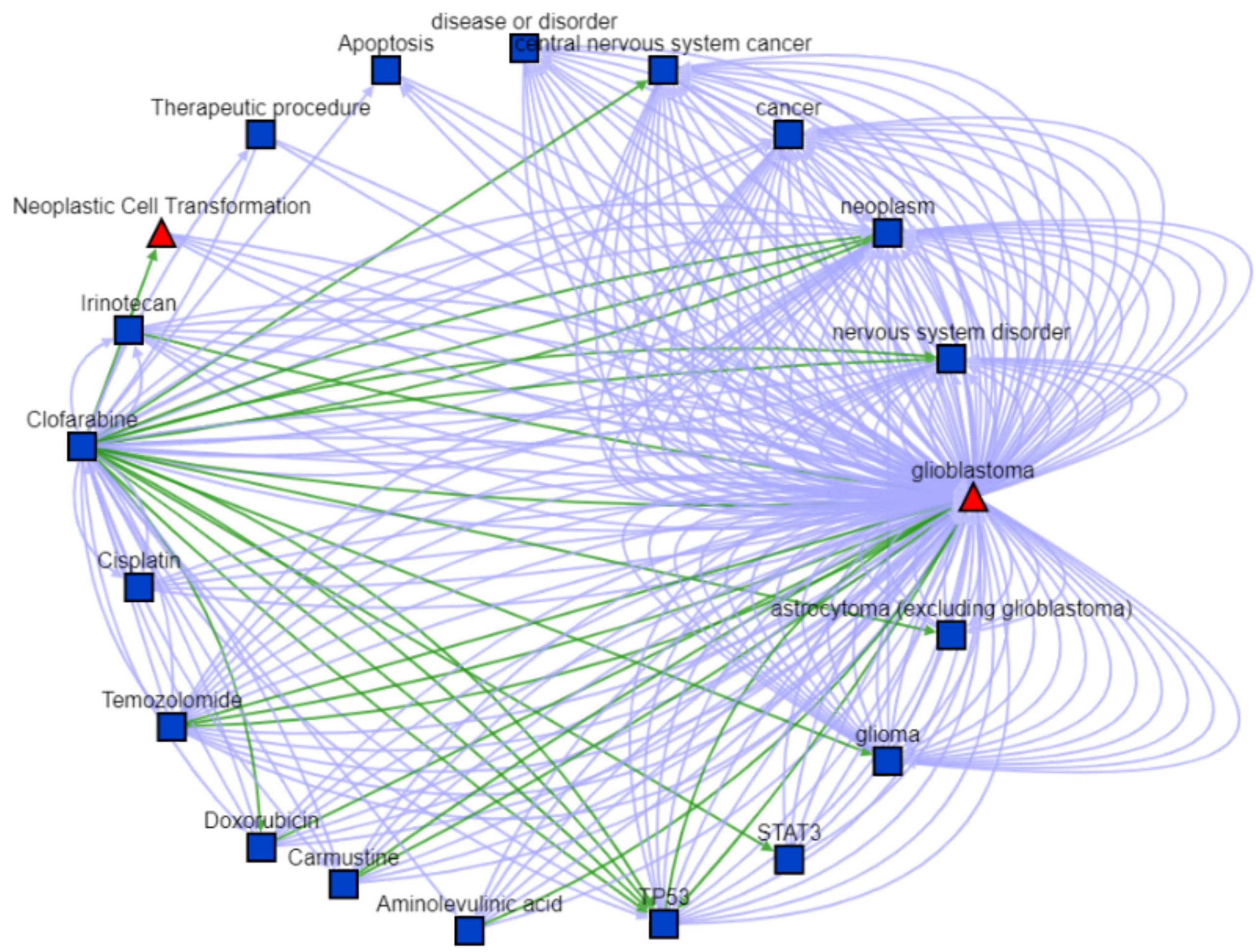

All nodes and edges:

MONDO:0000001 *disease or disorder*

biolink:occurs_together_in_literature_with → MONDO:0018177

biolink:occurs_together_in_literature_with → MONDO:0018177

← biolink:subclass_of MONDO:0018177

← biolink:subclass_of	MONDO:0018177
← biolink:subclass_of	MONDO:0018177
← biolink:subclass_of	MONDO:0018177
← biolink:subclass_of	MONDO:0018177
← biolink:subclass_of	MONDO:0018177
← biolink:subclass_of	MONDO:0018177
← biolink:subclass_of	MONDO:0018177
← biolink:subclass_of	MONDO:0018177
← biolink:subclass_of	MONDO:0005071

MONDO:0002714 *central nervous system cancer*

biolink:subclass_of →	MONDO:0005070
biolink:coexists_with →	MONDO:0018177
biolink:occurs_together_in_literature_with →	MONDO:0018177
biolink:occurs_together_in_literature_with →	MONDO:0018177
biolink:occurs_together_in_literature_with →	MONDO:0018177
← biolink:subclass_of	MONDO:0018177
← biolink:subclass_of	MONDO:0018177
← biolink:subclass_of	MONDO:0018177
← biolink:subclass_of	MONDO:0018177
← biolink:subclass_of	MONDO:0018177
← biolink:subclass_of	MONDO:0018177
← biolink:subclass_of	MONDO:0018177
← biolink:subclass_of	MONDO:0018177
← biolink:subclass_of	MONDO:0018177
← biolink:subclass_of	MONDO:0018177
← biolink:subclass_of	MONDO:0018177
← biolink:subclass_of	MONDO:0018177
← biolink:subclass_of	MONDO:0018177
← biolink:subclass_of	MONDO:0018177
← biolink:subclass_of	MONDO:0018177
← biolink:associated_with [q]	PUBCHEM.COMPOUND:119182
← biolink:subclass_of	MONDO:0018177

MONDO:0004992 *cancer*

biolink:affects →	MONDO:0018177
biolink:causes →	MONDO:0018177
biolink:coexists_with →	MONDO:0018177
biolink:occurs_together_in_literature_with →	MONDO:0018177
biolink:occurs_together_in_literature_with →	MONDO:0018177
← biolink:subclass_of	MONDO:0018177
← biolink:subclass_of	MONDO:0018177
← biolink:subclass_of	MONDO:0018177

ARAX User Interface

Documentation: Overview TRAPI 1.4.2 Resources

Input
Queries
Settings
List A 0
List B 0
Compare Lists
History 1

Output
Summary
Provenance
Knowledge Graph
Results 15
Messages 1671

Tools
Synonyms
Dev Info
System Activity
SmartAPI Info
Translator Testing
Reset All

Late Wildfowl

	← biolink:subclass_of	[illegible]
	← biolink:subclass_of	MONDO:0018177
	← biolink:subclass_of	MONDO:0018177
	← biolink:subclass_of	MONDO:0018177
	← biolink:subclass_of	MONDO:0018177
	← biolink:subclass_of	MONDO:0018177
	← biolink:subclass_of	MONDO:0018177
	← biolink:subclass_of	MONDO:0018177
	← biolink:treats	PUBCHEM.COMPOUND:119182
	← biolink:subclass_of	MONDO:0018177
	← biolink:subclass_of	MONDO:0018177
	← biolink:affects	MONDO:0018177
	← biolink:coexists_with	MONDO:0018177
	← biolink:subclass_of	MONDO:0018177
	← biolink:occurs_together_in_literature_with	PUBCHEM.COMPOUND:119182

**MONDO:0005070**	*neoplasm*	
	biolink:causes →	MONDO:0018177
	biolink:occurs_together_in_literature_with →	MONDO:0018177
	biolink:occurs_together_in_literature_with →	MONDO:0018177
	biolink:occurs_together_in_literature_with →	MONDO:0018177
	biolink:occurs_together_in_literature_with →	MONDO:0018177
	← biolink:subclass_of	MONDO:0018177
	← biolink:subclass_of	MONDO:0018177
	← biolink:subclass_of	MONDO:0018177
	← biolink:subclass_of	MONDO:0018177
	← biolink:subclass_of	MONDO:0018177
	← biolink:subclass_of	MONDO:0018177
	← biolink:subclass_of	MONDO:0018177
	← biolink:causes	PUBCHEM.COMPOUND:119182
	← biolink:subclass_of	MONDO:0018177
	← biolink:subclass_of	MONDO:0018177
	← biolink:subclass_of	MONDO:0018177
	← biolink:subclass_of	MONDO:0018177
	← biolink:subclass_of	MONDO:0018177
	← biolink:subclass_of	MONDO:0018177
	← biolink:causes	PUBCHEM.COMPOUND:119182
	← biolink:treats	PUBCHEM.COMPOUND:119182
	← biolink:subclass_of	MONDO:0018177
	← biolink:subclass_of	MONDO:0018177
	← biolink:subclass_of	MONDO:0002714
	← biolink:subclass_of	MONDO:0021042
	← biolink:associated_with [q]	PUBCHEM.COMPOUND:119182
	← biolink:has_side_effect [q]	PUBCHEM.COMPOUND:119182

← biolink:causes	[illegible]
← biolink:coexists_with	MONDO:0018177
← biolink:manifestation_of	MONDO:0018177
← biolink:subclass_of	MONDO:0018177
← biolink:treats	PUBCHEM.COMPOUND:119182
← biolink:occurs_together_in_literature_with	PUBCHEM.COMPOUND:119182
← biolink:occurs_together_in_literature_with	PUBCHEM.COMPOUND:119182

MONDO:0005071 *nervous system disorder*

biolink:subclass_of →	MONDO:0000001
biolink:occurs_together_in_literature_with →	MONDO:0018177
← biolink:subclass_of	MONDO:0018177
← biolink:subclass_of	MONDO:0018177
← biolink:subclass_of	MONDO:0018177
← biolink:subclass_of	MONDO:0018177
← biolink:subclass_of	MONDO:0018177
← biolink:subclass_of	MONDO:0018177
← biolink:subclass_of	MONDO:0018177
← biolink:causes	PUBCHEM.COMPOUND:119182
← biolink:subclass_of	MONDO:0018177
← biolink:subclass_of	MONDO:0018177
← biolink:subclass_of	MONDO:0018177
← biolink:subclass_of	MONDO:0018177
← biolink:subclass_of	MONDO:0018177
← biolink:subclass_of	MONDO:0018177
← biolink:causes	PUBCHEM.COMPOUND:119182
← biolink:subclass_of	MONDO:0018177
← biolink:subclass_of	MONDO:0018177
← biolink:associated_with [q]	PUBCHEM.COMPOUND:119182
← biolink:has_side_effect [q]	PUBCHEM.COMPOUND:119182
← biolink:subclass_of	MONDO:0018177

MONDO:0018177 *glioblastoma*

biolink:subclass_of →	MONDO:0000001
biolink:subclass_of →	MONDO:0002714
biolink:subclass_of →	MONDO:0004992
biolink:subclass_of →	MONDO:0005070
biolink:subclass_of →	MONDO:0005071
biolink:subclass_of →	MONDO:0021042
biolink:subclass_of →	MONDO:0002714
biolink:subclass_of →	MONDO:0005070
biolink:subclass_of →	MONDO:0005071
biolink:subclass_of →	MONDO:0021042
biolink:subclass_of →	MONDO:0000001

[illegible]	[illegible]
biolink:subclass_of →	MONDO:0005071
biolink:subclass_of →	MONDO:0021042
biolink:subclass_of →	MONDO:0002714
biolink:subclass_of →	MONDO:0004992
biolink:subclass_of →	MONDO:0005070
biolink:subclass_of →	MONDO:0005071
biolink:subclass_of →	MONDO:0021042
biolink:subclass_of →	MONDO:0000001
biolink:subclass_of →	MONDO:0002714
biolink:subclass_of →	MONDO:0004992
biolink:subclass_of →	MONDO:0005070
biolink:subclass_of →	MONDO:0005071
biolink:subclass_of →	MONDO:0021042
biolink:subclass_of →	MONDO:0000001
biolink:subclass_of →	MONDO:0002714
biolink:subclass_of →	MONDO:0004992
biolink:subclass_of →	MONDO:0021042
biolink:subclass_of →	MONDO:0000001
biolink:subclass_of →	MONDO:0002714
biolink:subclass_of →	MONDO:0004992
biolink:subclass_of →	MONDO:0005070
biolink:subclass_of →	MONDO:0005071
biolink:subclass_of →	MONDO:0021042
biolink:subclass_of →	MONDO:0002714
biolink:subclass_of →	MONDO:0004992
biolink:subclass_of →	MONDO:0005070
biolink:subclass_of →	MONDO:0005071
biolink:subclass_of →	MONDO:0021042
biolink:subclass_of →	MONDO:0000001
biolink:subclass_of →	MONDO:0002714
biolink:subclass_of →	MONDO:0004992
biolink:subclass_of →	MONDO:0005070
biolink:subclass_of →	MONDO:0005071
biolink:subclass_of →	MONDO:0021042
biolink:subclass_of →	MONDO:0002714
biolink:subclass_of →	MONDO:0004992
biolink:subclass_of →	MONDO:0005070
biolink:subclass_of →	MONDO:0005071
biolink:subclass_of →	MONDO:0021042
biolink:subclass_of →	MONDO:0000001
biolink:subclass_of →	MONDO:0002714
biolink:subclass_of →	MONDO:0004992
biolink:subclass_of →	MONDO:0005070

[illegible]	[illegible]
biolink:subclass_of ➔	MONDO:0004992
biolink:subclass_of ➔	MONDO:0005070
biolink:subclass_of ➔	MONDO:0005071
biolink:subclass_of ➔	MONDO:0000001
biolink:subclass_of ➔	MONDO:0002714
biolink:subclass_of ➔	MONDO:0004992
biolink:subclass_of ➔	MONDO:0005070
biolink:subclass_of ➔	MONDO:0005071
biolink:subclass_of ➔	MONDO:0021042
biolink:subclass_of ➔	MONDO:0000001
biolink:subclass_of ➔	MONDO:0002714
biolink:subclass_of ➔	MONDO:0004992
biolink:subclass_of ➔	MONDO:0005070
biolink:subclass_of ➔	MONDO:0005071
biolink:subclass_of ➔	MONDO:0021042
biolink:subclass_of ➔	MONDO:0000001
biolink:subclass_of ➔	MONDO:0002714
biolink:subclass_of ➔	MONDO:0004992
biolink:subclass_of ➔	MONDO:0005070
biolink:subclass_of ➔	MONDO:0005071
biolink:subclass_of ➔	MONDO:0021042
biolink:subclass_of ➔	MONDO:0000001
biolink:subclass_of ➔	MONDO:0002714
biolink:subclass_of ➔	MONDO:0004992
biolink:subclass_of ➔	MONDO:0005070
biolink:subclass_of ➔	MONDO:0005071
biolink:subclass_of ➔	MONDO:0021042
biolink:has_participant [q] ➔	NCBIGene:7157
biolink:affects ➔	MONDO:0004992
biolink:affects ➔	MONDO:0005070
biolink:affects ➔	UMLS:C0007621
biolink:affects ➔	UMLS:C0162638
biolink:causes ➔	MONDO:0005070
biolink:coexists_with ➔	MONDO:0004992
biolink:coexists_with ➔	MONDO:0005070
biolink:coexists_with ➔	MONDO:0019781
biolink:coexists_with ➔	MONDO:0021042
biolink:coexists_with ➔	UMLS:C0007621
biolink:coexists_with ➔	UMLS:C0162638
biolink:has_part ➔	NCBIGene:7157
biolink:manifestation_of ➔	MONDO:0005070
biolink:subclass_of ➔	MONDO:0002714
biolink:subclass_of ➔	MONDO:0004992

biolink:subclass_of →	[illegible]
biolink:subclass_of →	MONDO:0019781
biolink:subclass_of →	MONDO:0019781
biolink:subclass_of →	MONDO:0019781
biolink:subclass_of →	MONDO:0021042
biolink:subclass_of →	MONDO:0021042
biolink:subclass_of →	MONDO:0021042
← biolink:ameliorates	PUBCHEM.COMPOUND:2578
← biolink:ameliorates	PUBCHEM.COMPOUND:31703
← biolink:ameliorates	PUBCHEM.COMPOUND:5394
← biolink:ameliorates	PUBCHEM.COMPOUND:5460033
← biolink:ameliorates	PUBCHEM.COMPOUND:60838
← biolink:has_adverse_event	PUBCHEM.COMPOUND:5394
← biolink:genetically_associated_with	NCBIGene:7157
← biolink:genetically_associated_with	NCBIGene:7157
← biolink:contributes_to	NCBIGene:6774
← biolink:contributes_to	NCBIGene:7157
← biolink:genetically_associated_with	NCBIGene:7157
← biolink:genetically_associated_with	NCBIGene:7157
← biolink:has_phenotype	NCBIGene:7157
← biolink:treats	PUBCHEM.COMPOUND:119182
← biolink:treats	PUBCHEM.COMPOUND:137
← biolink:ameliorates	PUBCHEM.COMPOUND:2578
← biolink:treats	PUBCHEM.COMPOUND:2578
← biolink:ameliorates	PUBCHEM.COMPOUND:31703
← biolink:treats	PUBCHEM.COMPOUND:31703
← biolink:ameliorates	PUBCHEM.COMPOUND:5394
← biolink:has_adverse_event	PUBCHEM.COMPOUND:5394
← biolink:treats	PUBCHEM.COMPOUND:5394
← biolink:ameliorates	PUBCHEM.COMPOUND:5460033
← biolink:treats	PUBCHEM.COMPOUND:5460033
← biolink:ameliorates	PUBCHEM.COMPOUND:60838
← biolink:treats	PUBCHEM.COMPOUND:60838
← biolink:associated_with [q]	PUBCHEM.COMPOUND:119182
← biolink:treats [q]	PUBCHEM.COMPOUND:137
← biolink:treats [q]	PUBCHEM.COMPOUND:2578
← biolink:treats [q]	PUBCHEM.COMPOUND:2578
← biolink:treats [q]	PUBCHEM.COMPOUND:31703
← biolink:treats [q]	PUBCHEM.COMPOUND:5394
← biolink:treats [q]	PUBCHEM.COMPOUND:5394
← biolink:treats [q]	PUBCHEM.COMPOUND:60838
← biolink:coexists_with	MONDO:0002714
← biolink:affects	MONDO:0004992
← biolink:causes	MONDO:0004992

ARAX User Interface

← biolink:coexists_with	MONDO:[illegible]
← biolink:coexists_with	MONDO:0021042
← biolink:affects	NCBIGene:6774
← biolink:gene_associated_with_condition	NCBIGene:6774
← biolink:gene_associated_with_condition	NCBIGene:6774
← biolink:predisposes	NCBIGene:6774
← biolink:related_to	NCBIGene:6774
← biolink:affects	NCBIGene:7157
← biolink:affects [q]	NCBIGene:7157
← biolink:causes	NCBIGene:7157
← biolink:gene_associated_with_condition	NCBIGene:7157
← biolink:gene_associated_with_condition	NCBIGene:7157
← biolink:related_to	NCBIGene:7157
← biolink:related_to	PUBCHEM.COMPOUND:137
← biolink:treats	PUBCHEM.COMPOUND:137
← biolink:treats	PUBCHEM.COMPOUND:137
← biolink:treats	PUBCHEM.COMPOUND:137
← biolink:treats	PUBCHEM.COMPOUND:2578
← biolink:treats	PUBCHEM.COMPOUND:2578
← biolink:treats	PUBCHEM.COMPOUND:31703
← biolink:treats	PUBCHEM.COMPOUND:31703
← biolink:affects [q]	PUBCHEM.COMPOUND:5394
← biolink:causes	PUBCHEM.COMPOUND:5394
← biolink:disrupts	PUBCHEM.COMPOUND:5394
← biolink:related_to	PUBCHEM.COMPOUND:5394
← biolink:treats	PUBCHEM.COMPOUND:5394
← biolink:treats	PUBCHEM.COMPOUND:5394
← biolink:treats	PUBCHEM.COMPOUND:5394
← biolink:related_to	PUBCHEM.COMPOUND:5460033
← biolink:related_to	PUBCHEM.COMPOUND:60838
← biolink:treats	PUBCHEM.COMPOUND:60838
← biolink:treats	PUBCHEM.COMPOUND:60838
← biolink:causes	UMLS:C0007621
← biolink:coexists_with	UMLS:C0007621
← biolink:prevents	UMLS:C0087111
← biolink:treats	UMLS:C0087111
← biolink:affects	UMLS:C0162638
← biolink:occurs_in	UMLS:C0162638
← biolink:treats	PUBCHEM.COMPOUND:119182
← biolink:treats	PUBCHEM.COMPOUND:119182
← biolink:occurs_together_in_literature_with	PUBCHEM.COMPOUND:2578
← biolink:occurs_together_in_literature_with	PUBCHEM.COMPOUND:5460033
← biolink:occurs_together_in_literature_with	PUBCHEM.COMPOUND:119182
← biolink:occurs_together_in_literature_with	PUBCHEM.COMPOUND:60838

ARAX User Interface

← biolink:occurs_together_in_literature_with	MONDO:0005070
← biolink:occurs_together_in_literature_with	MONDO:0005070
← biolink:occurs_together_in_literature_with	MONDO:0019781
← biolink:occurs_together_in_literature_with	MONDO:0021042
← biolink:occurs_together_in_literature_with	MONDO:0021042
← biolink:occurs_together_in_literature_with	MONDO:0002714
← biolink:occurs_together_in_literature_with	MONDO:0002714
← biolink:occurs_together_in_literature_with	MONDO:0000001
← biolink:occurs_together_in_literature_with	MONDO:0000001
← biolink:occurs_together_in_literature_with	MONDO:0002714
← biolink:occurs_together_in_literature_with	MONDO:0004992
← biolink:occurs_together_in_literature_with	MONDO:0004992
← biolink:occurs_together_in_literature_with	MONDO:0005070
← biolink:occurs_together_in_literature_with	MONDO:0005070
← biolink:occurs_together_in_literature_with	MONDO:0005071
← biolink:occurs_together_in_literature_with	MONDO:0019781
← biolink:occurs_together_in_literature_with	MONDO:0021042
← biolink:occurs_together_in_literature_with	MONDO:0021042

MONDO:0019781

astrocytoma (excluding glioblastoma)

biolink:coexists_with →	MONDO:0018177
biolink:occurs_together_in_literature_with →	MONDO:0018177
biolink:occurs_together_in_literature_with →	MONDO:0018177
← biolink:associated_with [q]	PUBCHEM.COMPOUND:119182
← biolink:coexists_with	MONDO:0018177
← biolink:subclass_of	MONDO:0018177
← biolink:subclass_of	MONDO:0018177
← biolink:subclass_of	MONDO:0018177
← biolink:subclass_of	MONDO:0018177

MONDO:0021042

glioma

biolink:subclass_of →	MONDO:0005070
biolink:coexists_with →	MONDO:0018177
biolink:occurs_together_in_literature_with →	MONDO:0018177
biolink:occurs_together_in_literature_with →	MONDO:0018177
biolink:occurs_together_in_literature_with →	MONDO:0018177
biolink:occurs_together_in_literature_with →	MONDO:0018177
← biolink:subclass_of	MONDO:0018177
← biolink:subclass_of	MONDO:0018177
← biolink:subclass_of	MONDO:0018177
← biolink:subclass_of	MONDO:0018177
← biolink:subclass_of	MONDO:0018177
← biolink:subclass_of	MONDO:0018177

← biolink:subclass_of	[illegible]
← biolink:subclass_of	MONDO:0018177
← biolink:subclass_of	MONDO:0018177
← biolink:subclass_of	MONDO:0018177
← biolink:subclass_of	MONDO:0018177
← biolink:subclass_of	MONDO:0018177
← biolink:subclass_of	MONDO:0018177
← biolink:associated_with [q]	PUBCHEM.COMPOUND:119182
← biolink:coexists_with	MONDO:0018177
← biolink:subclass_of	MONDO:0018177
← biolink:subclass_of	MONDO:0018177
← biolink:subclass_of	MONDO:0018177

NCBIGene:6774 *STAT3*

biolink:contributes_to →	MONDO:0018177
biolink:affects →	MONDO:0018177
biolink:gene_associated_with_condition →	MONDO:0018177
biolink:gene_associated_with_condition →	MONDO:0018177
biolink:predisposes →	MONDO:0018177
biolink:related_to →	MONDO:0018177
← biolink:affects [q]	PUBCHEM.COMPOUND:119182

NCBIGene:7157 *TP53*

biolink:genetically_associated_with →	MONDO:0018177
biolink:genetically_associated_with →	MONDO:0018177
biolink:contributes_to →	MONDO:0018177
biolink:genetically_associated_with →	MONDO:0018177
biolink:genetically_associated_with →	MONDO:0018177
biolink:has_phenotype →	MONDO:0018177
biolink:affects →	MONDO:0018177
biolink:affects [q] →	MONDO:0018177
biolink:causes →	MONDO:0018177
biolink:gene_associated_with_condition →	MONDO:0018177
biolink:gene_associated_with_condition →	MONDO:0018177
biolink:related_to →	MONDO:0018177
← biolink:affects	PUBCHEM.COMPOUND:119182
← biolink:causes	PUBCHEM.COMPOUND:119182
← biolink:affects [q]	PUBCHEM.COMPOUND:119182
← biolink:affects [q]	PUBCHEM.COMPOUND:119182
← biolink:affects [q]	PUBCHEM.COMPOUND:119182
← biolink:has_participant [q]	MONDO:0018177
← biolink:affects [q]	PUBCHEM.COMPOUND:119182
← biolink:has_part	MONDO:0018177

ARAX User Interface

Documentation: Overview TRAPI 1.4.2 Resources

Input
Queries
Settings
List A 0
List B 0
Compare Lists
History 1

Output
Summary
Provenance
Knowledge Graph
Results 15
Messages 1671

Tools
Synonyms
Dev Info
System Activity
SmartAPI Info
Translator Testing
Reset All

Late Wildfowl

biolink:causes ➔	NCBIGene:7157
biolink:affects [q] ➔	NCBIGene:7157
biolink:causes ➔	MONDO:0005070
biolink:causes ➔	MONDO:0005071
biolink:affects [q] ➔	NCBIGene:7157
biolink:affects [q] ➔	NCBIGene:6774
biolink:affects [q] ➔	NCBIGene:7157
biolink:causes ➔	MONDO:0005070
biolink:causes ➔	MONDO:0005071
biolink:treats ➔	MONDO:0004992
biolink:treats ➔	MONDO:0005070
biolink:treats ➔	MONDO:0018177
biolink:affects [q] ➔	NCBIGene:7157
biolink:associated_with [q] ➔	MONDO:0002714
biolink:associated_with [q] ➔	MONDO:0005070
biolink:associated_with [q] ➔	MONDO:0005071
biolink:associated_with [q] ➔	MONDO:0018177
biolink:associated_with [q] ➔	MONDO:0019781
biolink:associated_with [q] ➔	MONDO:0021042
biolink:associated_with [q] ➔	UMLS:C0007621
biolink:correlated_with [q] ➔	PUBCHEM.COMPOUND:31703
biolink:has_side_effect [q] ➔	MONDO:0005070
biolink:has_side_effect [q] ➔	MONDO:0005071
biolink:treats [q] ➔	MONDO:0005070
biolink:causes ➔	UMLS:C0162638
biolink:physically_interacts_with ➔	PUBCHEM.COMPOUND:2578
biolink:physically_interacts_with ➔	PUBCHEM.COMPOUND:31703
biolink:physically_interacts_with ➔	PUBCHEM.COMPOUND:5394
biolink:physically_interacts_with ➔	PUBCHEM.COMPOUND:5460033
biolink:physically_interacts_with ➔	PUBCHEM.COMPOUND:60838
biolink:subclass_of ➔	UMLS:C0087111
biolink:treats ➔	MONDO:0005070
biolink:treats ➔	MONDO:0018177
biolink:treats ➔	MONDO:0018177
biolink:occurs_together_in_literature_with ➔	PUBCHEM.COMPOUND:2578
biolink:occurs_together_in_literature_with ➔	PUBCHEM.COMPOUND:5460033
biolink:occurs_together_in_literature_with ➔	PUBCHEM.COMPOUND:60838
biolink:occurs_together_in_literature_with ➔	PUBCHEM.COMPOUND:5394
biolink:occurs_together_in_literature_with ➔	PUBCHEM.COMPOUND:137
biolink:occurs_together_in_literature_with ➔	PUBCHEM.COMPOUND:31703
biolink:occurs_together_in_literature_with ➔	MONDO:0005070
biolink:occurs_together_in_literature_with ➔	MONDO:0004992
biolink:occurs_together_in_literature_with ➔	MONDO:0005070
biolink:occurs_together_in_literature_with ➔	MONDO:0018177

← biolink:physically_interacts_with	PUBCHEM.COMPOUND:[illegible]
← biolink:physically_interacts_with	PUBCHEM.COMPOUND:5460033
← biolink:physically_interacts_with	PUBCHEM.COMPOUND:60838
← biolink:has_input	UMLS:C0087111

PUBCHEM.COMPOUND:137 *Aminolevulinic acid*

biolink:treats →	MONDO:0018177
biolink:treats [q] →	MONDO:0018177
biolink:related_to →	MONDO:0018177
biolink:treats →	MONDO:0018177
biolink:treats →	MONDO:0018177
biolink:treats →	MONDO:0018177
biolink:occurs_together_in_literature_with →	MONDO:0018177
← biolink:occurs_together_in_literature_with	PUBCHEM.COMPOUND:119182

PUBCHEM.COMPOUND:2578 *Carmustine*

biolink:ameliorates →	MONDO:0018177
biolink:ameliorates →	MONDO:0018177
biolink:treats →	MONDO:0018177
biolink:treats [q] →	MONDO:0018177
biolink:treats [q] →	MONDO:0018177
biolink:physically_interacts_with →	PUBCHEM.COMPOUND:119182
biolink:treats →	MONDO:0018177
biolink:treats →	MONDO:0018177
biolink:occurs_together_in_literature_with →	MONDO:0018177
← biolink:physically_interacts_with	PUBCHEM.COMPOUND:119182
← biolink:occurs_together_in_literature_with	PUBCHEM.COMPOUND:119182

PUBCHEM.COMPOUND:31703 *Doxorubicin*

biolink:ameliorates →	MONDO:0018177
biolink:ameliorates →	MONDO:0018177
biolink:treats →	MONDO:0018177
biolink:treats [q] →	MONDO:0018177
biolink:physically_interacts_with →	PUBCHEM.COMPOUND:119182
biolink:treats →	MONDO:0018177
biolink:treats →	MONDO:0018177
biolink:occurs_together_in_literature_with →	MONDO:0018177
← biolink:correlated_with [q]	PUBCHEM.COMPOUND:119182
← biolink:physically_interacts_with	PUBCHEM.COMPOUND:119182
← biolink:occurs_together_in_literature_with	PUBCHEM.COMPOUND:119182

PUBCHEM.COMPOUND:5394 *Temozolomide*

biolink:ameliorates →	MONDO:0018177
biolink:has_adverse_event →	MONDO:0018177

[illegible]

biolink:treats [q] → MONDO:0018177
biolink:treats [q] → MONDO:0018177
biolink:affects [q] → MONDO:0018177
biolink:causes → MONDO:0018177
biolink:disrupts → MONDO:0018177
biolink:physically_interacts_with → PUBCHEM.COMPOUND:119182
biolink:related_to → MONDO:0018177
biolink:treats → MONDO:0018177
biolink:treats → MONDO:0018177
biolink:treats → MONDO:0018177
biolink:occurs_together_in_literature_with → MONDO:0018177
← biolink:physically_interacts_with PUBCHEM.COMPOUND:119182
← biolink:occurs_together_in_literature_with PUBCHEM.COMPOUND:119182

PUBCHEM.COMPOUND:5460033 *Cisplatin*

biolink:ameliorates → MONDO:0018177
biolink:ameliorates → MONDO:0018177
biolink:treats → MONDO:0018177
biolink:physically_interacts_with → PUBCHEM.COMPOUND:119182
biolink:related_to → MONDO:0018177
biolink:occurs_together_in_literature_with → MONDO:0018177
← biolink:physically_interacts_with PUBCHEM.COMPOUND:119182
← biolink:occurs_together_in_literature_with PUBCHEM.COMPOUND:119182

PUBCHEM.COMPOUND:60838 *Irinotecan*

biolink:ameliorates → MONDO:0018177
biolink:ameliorates → MONDO:0018177
biolink:treats → MONDO:0018177
biolink:treats [q] → MONDO:0018177
biolink:physically_interacts_with → PUBCHEM.COMPOUND:119182
biolink:related_to → MONDO:0018177
biolink:treats → MONDO:0018177
biolink:treats → MONDO:0018177
biolink:occurs_together_in_literature_with → MONDO:0018177
← biolink:physically_interacts_with PUBCHEM.COMPOUND:119182
← biolink:occurs_together_in_literature_with PUBCHEM.COMPOUND:119182

UMLS:C0007621 *Neoplastic Cell Transformation*

biolink:causes → MONDO:0018177
biolink:coexists_with → MONDO:0018177
← biolink:associated_with [q] PUBCHEM.COMPOUND:119182
← biolink:affects MONDO:0018177
← biolink:coexists_with MONDO:0018177

Input
Queries
Settings
List A 0
List B 0
Compare Lists
History 1
Output
Summary
Provenance
Knowledge Graph
Results 15
Messages 1671
Tools
Synonyms
Dev Info
System Activity
SmartAPI Info
Translator Testing
Reset All
Late Wildfowl

	biolink:prevents →	MONDO:0018177
	biolink:treats →	MONDO:0018177
	← biolink:subclass_of	PUBCHEM.COMPOUND:119182
**UMLS:C0162638**	*Apoptosis*	
