# Supplementary material for: Reversal gene expression assessment for drug repurposing, a case study of glioblastoma
Source: J Transl Med. 2025 Jan 7;23:25. doi: 10.1186/s12967-024-06046-1 (PMC11706105; doi:10.1186/s12967-024-06046-1)
Supplement: Supplementary file 4 — Additional file 4 [file 12967_2024_6046_MOESM4_ESM.pdf]

ARAX User Interface

Documentation: Overview TRAPI 1.4.2 Resources

Input

Queries

Settings

List A 0

List B 0

Compare Lists

History 2

Output

Summary

Provenance

Knowledge Graph

Results 10

Messages 1353

Tools

Synonyms

Dev Info

System Activity

SmartAPI Info

Translator Testing

Reset All

Late Wildfowl

KNOWLEDGE GRAPH

↻ B F C R

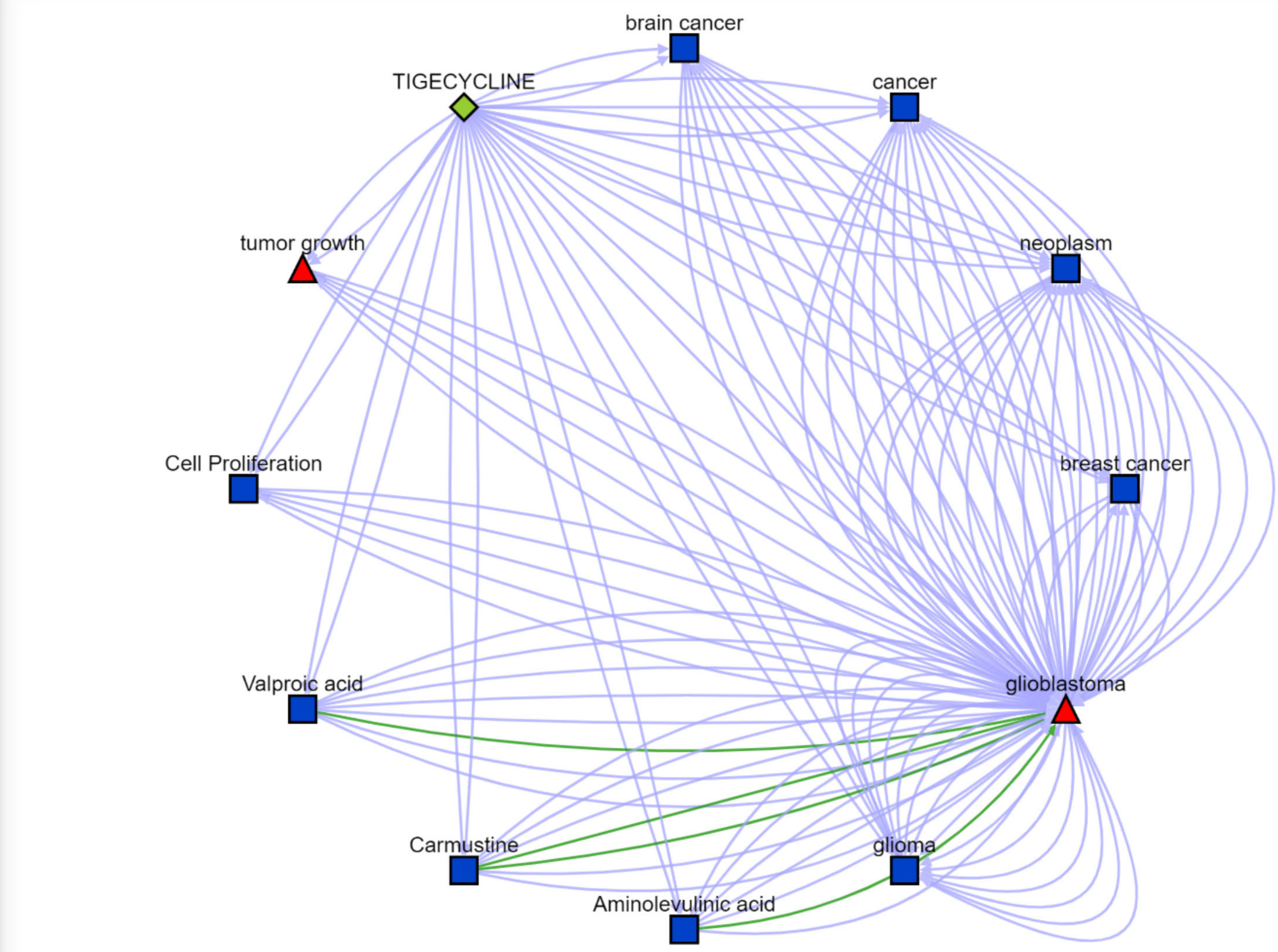

Click on a node or edge to get details

ARAX User Interface

Documentation: Overview TRAPI 1.4.2 Resources

Input

Queries

Settings

List A 0

List B 0

Compare Lists

History 2

Output

Summary

Provenance

Knowledge Graph

Results 10

Messages 1353

Tools

Synonyms

Dev Info

System Activity

SmartAPI Info

Translator Testing

Reset All

Late Wildfowl

ARAX User Interface

Documentation: Overview TRAPI 1.4.2 Resources

Input

Queries

Settings

List A 0

List B 0

Compare Lists

History 2

Output

Summary

Provenance

Knowledge Graph

Results 10

Messages 1353

Tools

Synonyms

Dev Info

System Activity

SmartAPI Info

Translator Testing

Reset All

Late Wildfowl

ARAX User Interface

Documentation: Overview TRAPI 1.4.2 Resources

Input

Queries

Settings

List A 0

List B 0

Compare Lists

History 2

Output

Summary

Provenance

Knowledge Graph

Results 10

Messages 1353

Tools

Synonyms

Dev Info

System Activity

SmartAPI Info

Translator Testing

Reset All

Late Wildfowl

ARAX User Interface

Documentation: Overview TRAPI 1.4.2 Resources

Input

Queries

Settings

List A 0

List B 0

Compare Lists

History 2

Output

Summary

Provenance

Knowledge Graph

Results 10

Messages 1353

Tools

Synonyms

Dev Info

System Activity

SmartAPI Info

Translator Testing

Reset All

Late Wildfowl

ARAX User Interface

Documentation: Overview TRAPI 1.4.2 Resources

Input

Queries

Settings

List A 0

List B 0

Compare Lists

History 2

Output

Summary

Provenance

Knowledge Graph

Results 10

Messages 1353

Tools

Synonyms

Dev Info

System Activity

SmartAPI Info

Translator Testing

Reset All

Late Wildfowl

ARAX User Interface

Documentation: Overview TRAPI 1.4.2 Resources

Input

Queries

Settings

List A 0

List B 0

Compare Lists

History 2

Output

Summary

Provenance

Knowledge Graph

Results 10

Messages 1353

Tools

Synonyms

Dev Info

System Activity

SmartAPI Info

Translator Testing

Reset All

Late Wildfowl

ARAX User Interface

Documentation: Overview TRAPI 1.4.2 Resources

Input

Queries

Settings

List A 0

List B 0

Compare Lists

History 2

Output

Summary

Provenance

Knowledge Graph

Results 10

Messages 1353

Tools

Synonyms

Dev Info

System Activity

SmartAPI Info

Translator Testing

Reset All

Late Wildfowl
