# Supplementary material for: Reversal gene expression assessment for drug repurposing, a case study of glioblastoma
Source: J Transl Med. 2025 Jan 7;23:25. doi: 10.1186/s12967-024-06046-1 (PMC11706105; doi:10.1186/s12967-024-06046-1)
Supplement: Supplementary file 5 — Additional file 5 [file 12967_2024_6046_MOESM5_ESM.pdf]

DMSO | 10µM staurosporine | 1.2 µM Clofarabine | 1.2 µM Ciclopirox

U87MG

A172

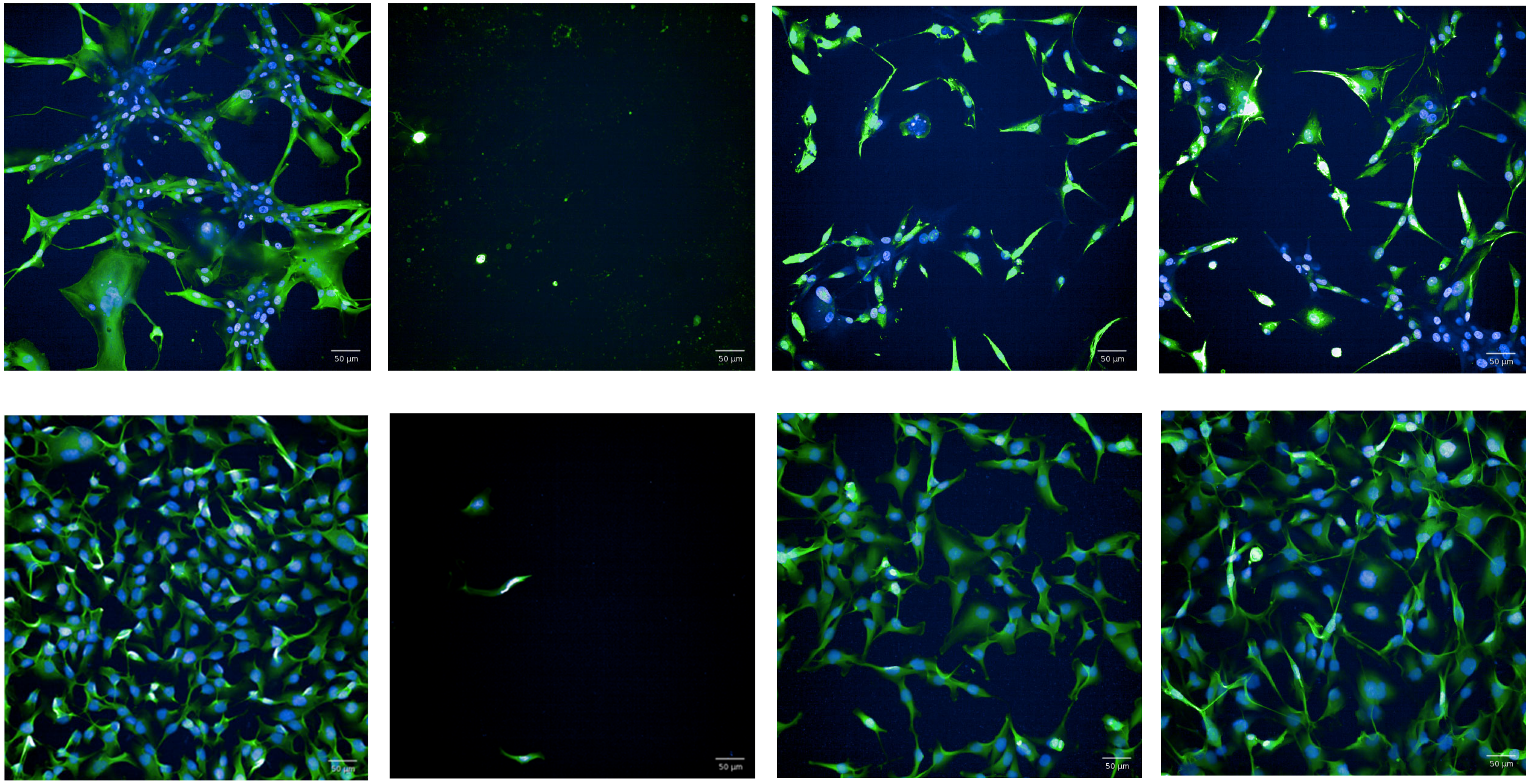

Blue= nuclei
Green= HC CMgreen

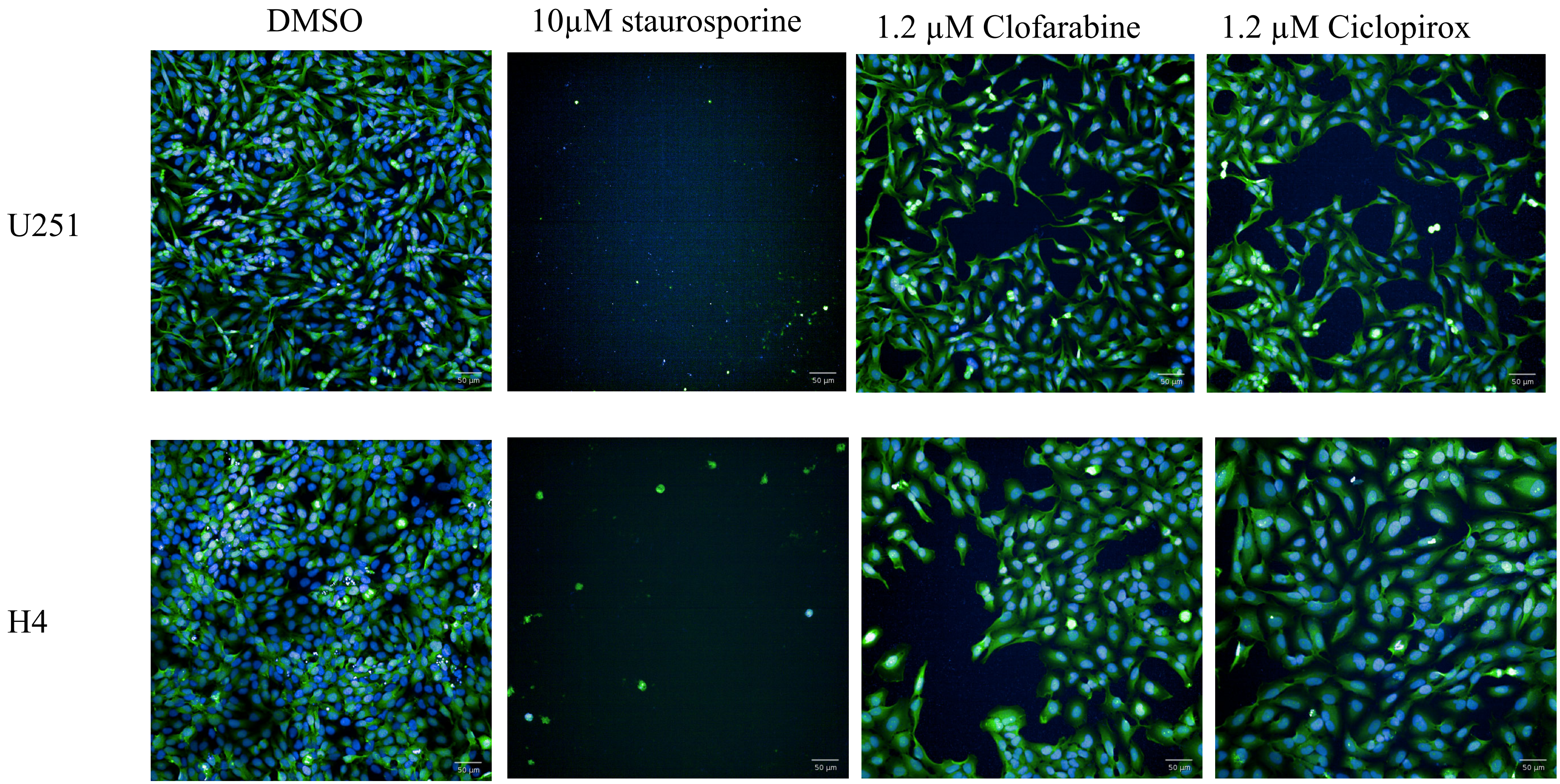
DMSO
10µM staurosporine
1.2 µM Clofarabine
1.2 µM Ciclopirox
U251
H4

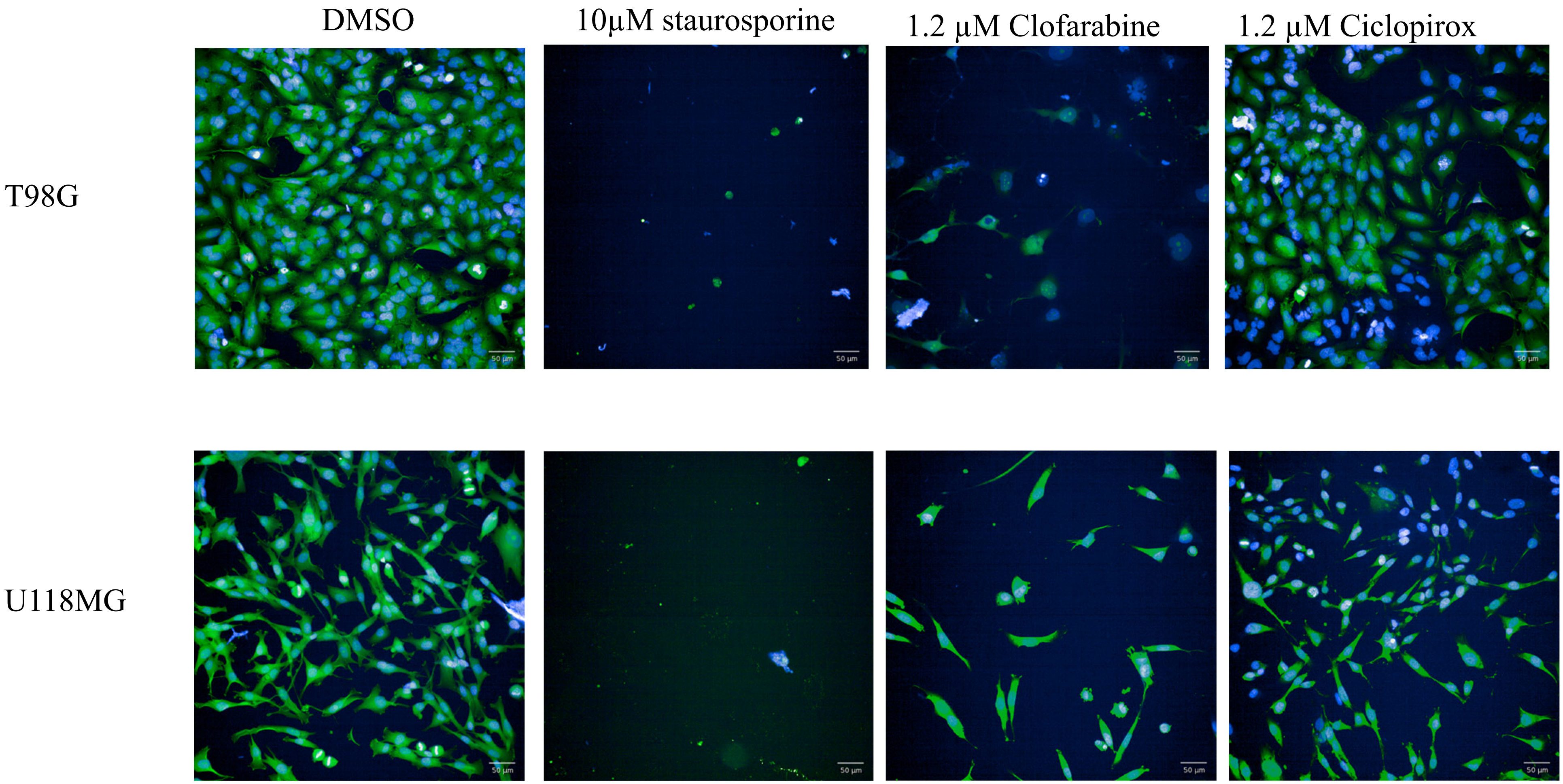
DMSO
10μM staurosporine
1.2 μM Clofarabine
1.2 μM Ciclopirox
T98G
U118MG

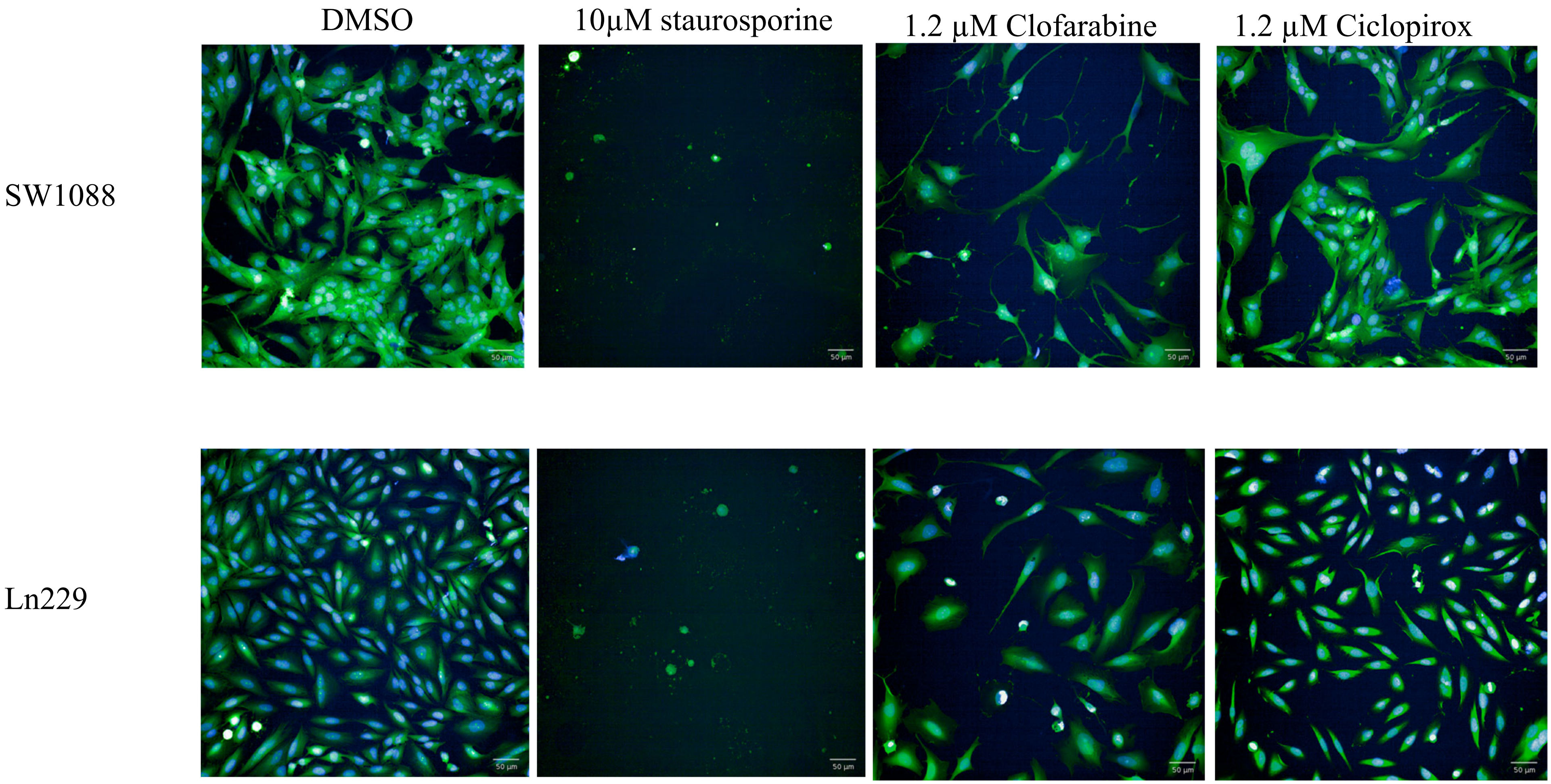
DMSO
10μM staurosporine
1.2 μM Clofarabine
1.2 μM Ciclopirox
SW1088
Ln229
50 μm

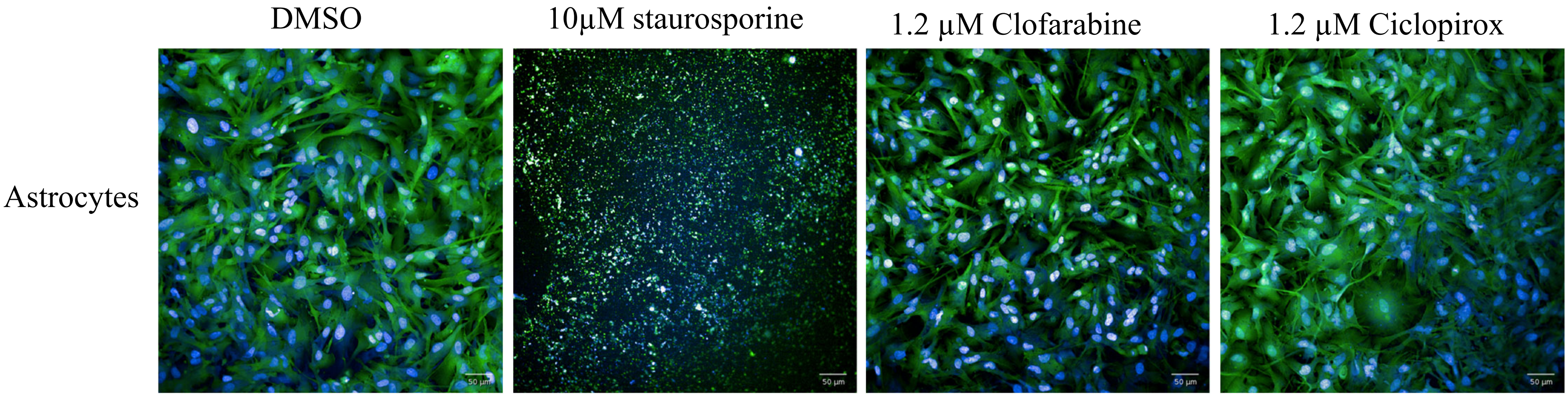
DMSO
10µM staurosporine
1.2 µM Clofarabine
1.2 µM Ciclopirox
Astrocytes
50 µm
50 µm
50 µm
50 µm
