# Supplementary figures and images for: Reversal gene expression assessment for drug repurposing, a case study of glioblastoma
Source: J Transl Med. 2025 Jan 7;23:25. doi: 10.1186/s12967-024-06046-1 (PMC11706105; doi:10.1186/s12967-024-06046-1)

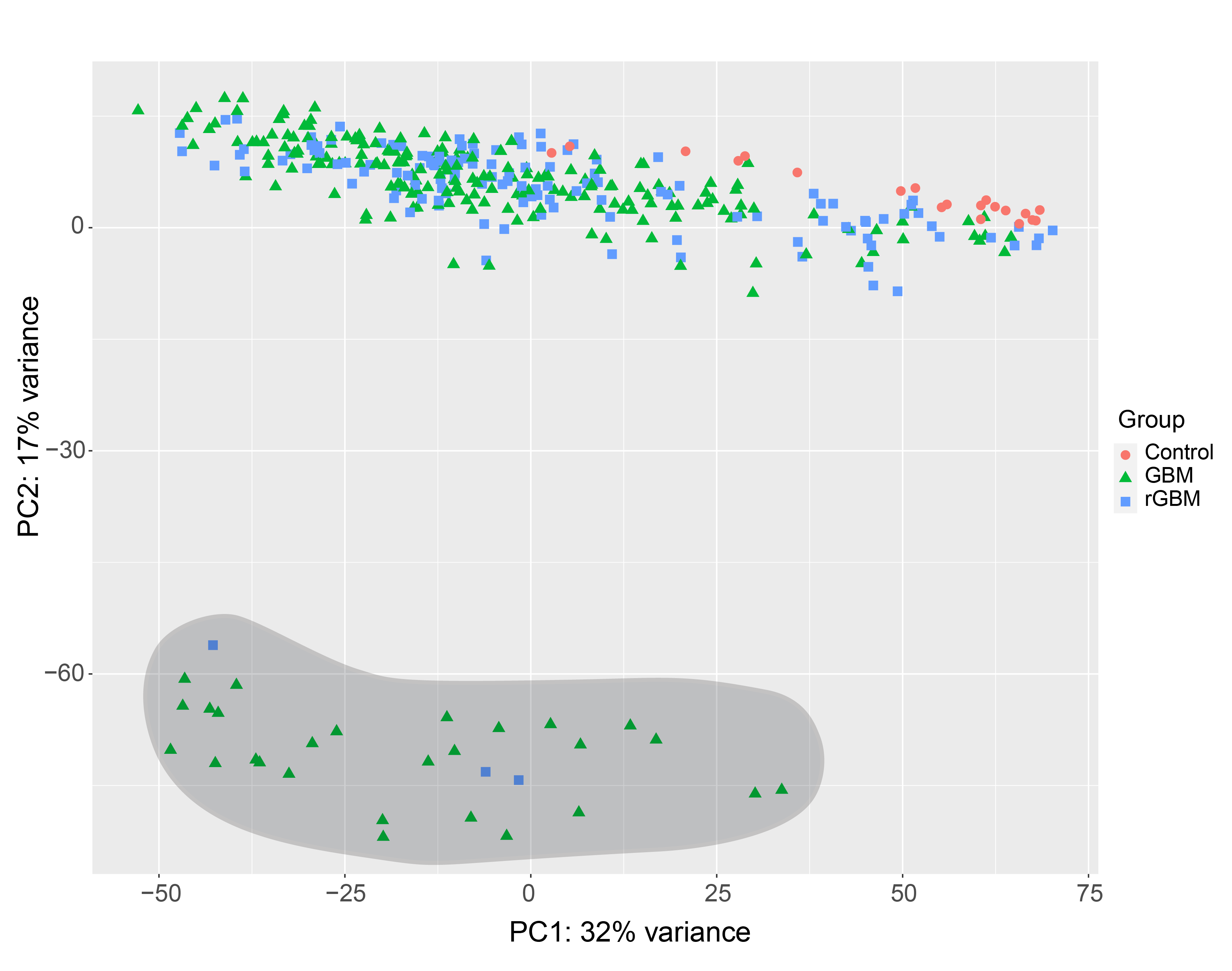

Supplement: Supplementary file 6 — Additional file 6 [file 12967_2024_6046_MOESM6_ESM.png]

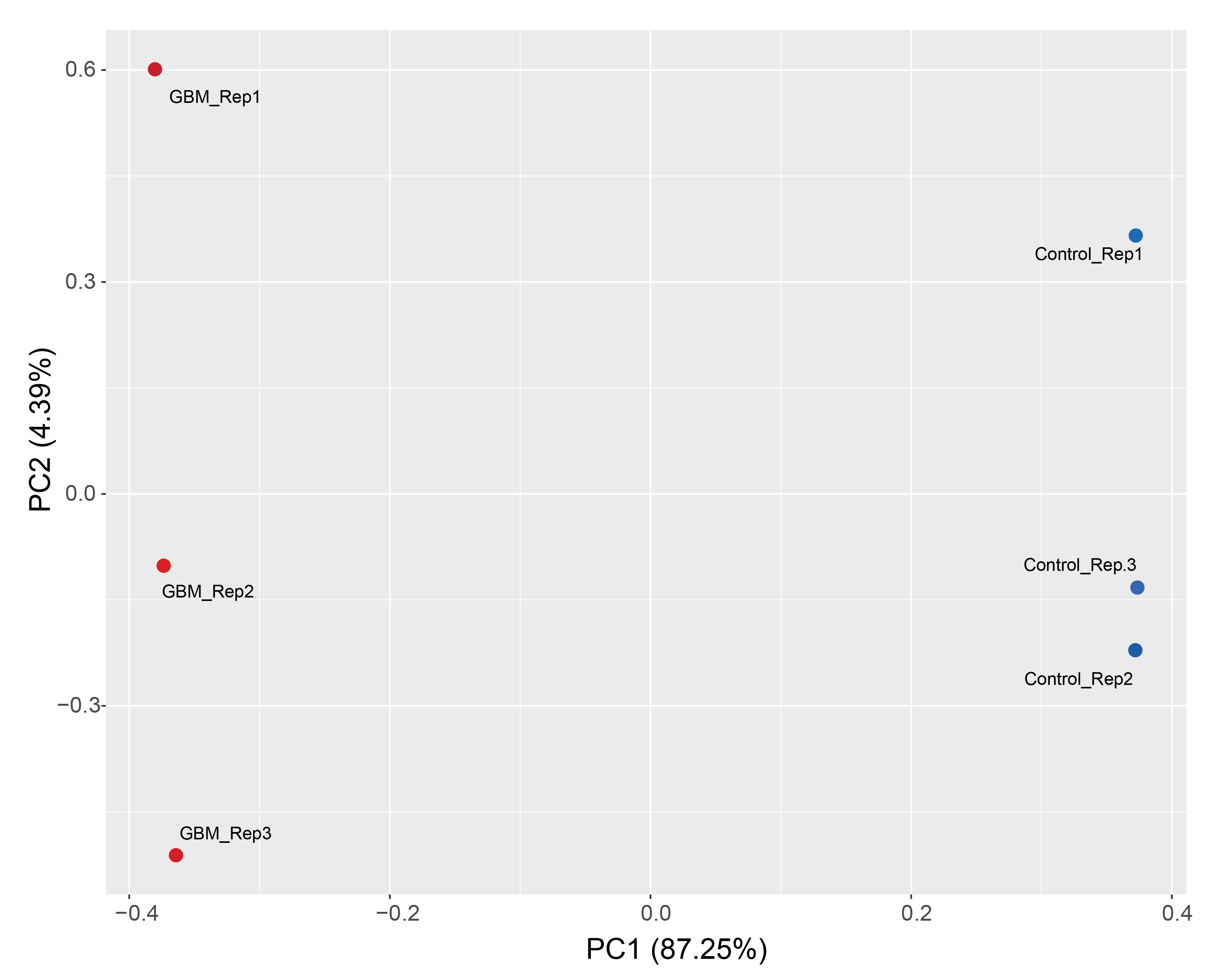

Supplement: Supplementary file 7 — Additional file 7 [file 12967_2024_6046_MOESM7_ESM.png]

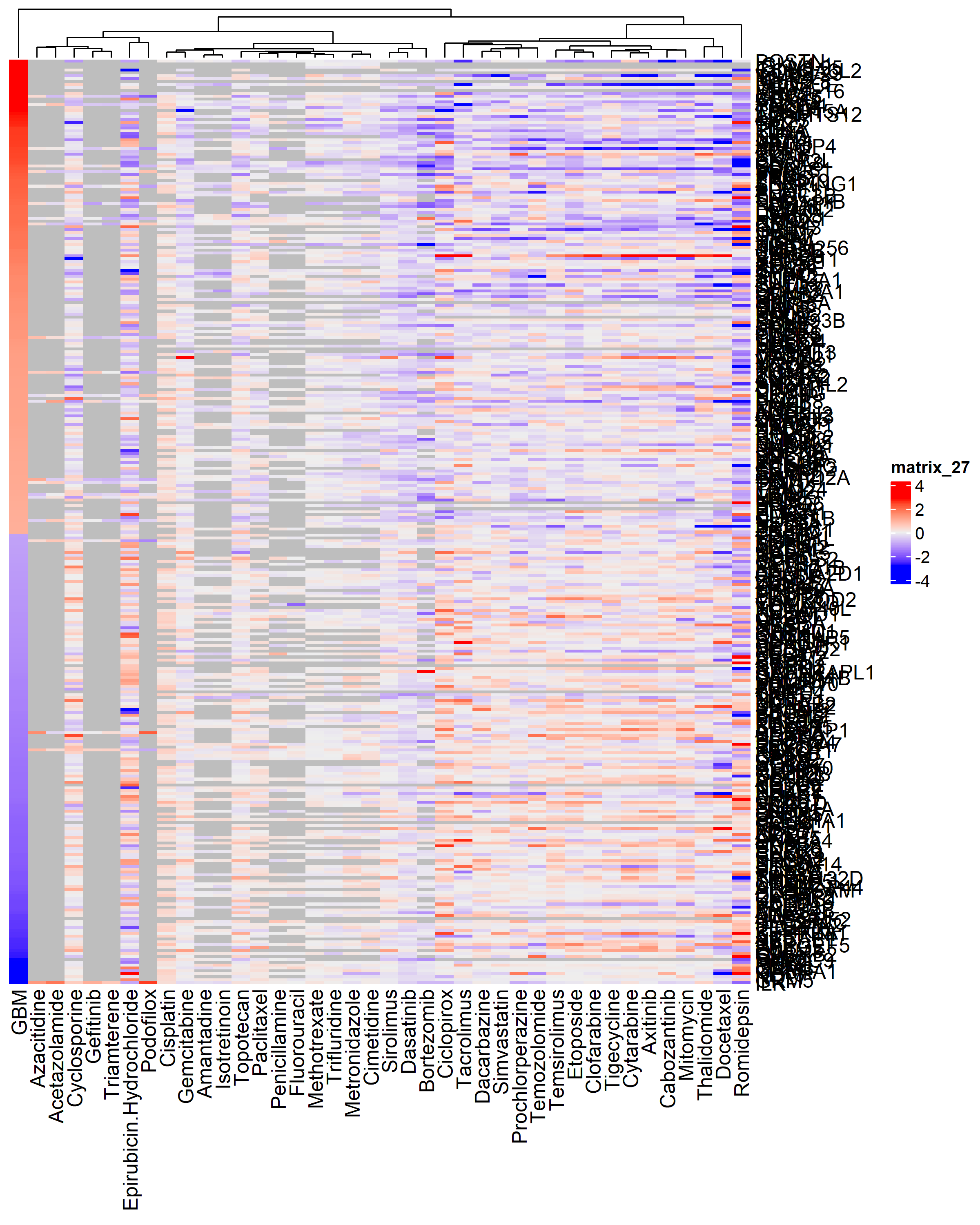

Supplement: Supplementary file 8 — Additional file 8 [file 12967_2024_6046_MOESM8_ESM.png]
