# Supplementary material for: Reversal gene expression assessment for drug repurposing, a case study of glioblastoma
Source: J Transl Med. 2025 Jan 7;23:25. doi: 10.1186/s12967-024-06046-1 (PMC11706105; doi:10.1186/s12967-024-06046-1)
Supplement: Supplementary file 9 — Additional file 9 [file 12967_2024_6046_MOESM9_ESM.docx]

similarity study and regulation score development

###### Introduction

The main idea to index the potential of a repurposing drug when evaluating their gene expression features is the similarity.

There are several indices being used in drug repurposing (see table 1).

However, there are no index developed particularly for the repurposing. All these are borrowed from other areas.

The other disadvantage is that all these indices do not take account in the significance of the gene expression levels. It is the same situation with the most commonly used similarity matrix such as "Euclidean", "maximum", "Manhattan", "Canberra", "binary", and "Minkowski".

An ideal matrix should be able to reflect the number of genes a medicine can regulate and the strength it can regulate.

Kullback-Leibler (KL) Divergence is a measure from information theory that can be used to compare two probability distributions. In the context of ranked lists, it quantifies how much one list differs from another in terms of their underlying probability distributions.

Combine the concept of running sum, spearman rank correlation, and Kullback–Leibler (KL) divergence (1),

KL divergence was built up upon Shannon entropy in Information theory (2). In Information theory, KL divergence reflects the excess entropy between two distributions. This divergence reflects weight of the original probability of all x in the distribution and their fold changes. KL distance is non-negative, with a value of 0 indicating that the two distributions are identical, and a value greater than 0 indicating that the two distributions are different.

So based on KL, we derived the formula to fit the features of gene expression matrix. Steps as below:

###### Method

Formula Derivation:

The calculation of Basic K-L divergence or distance:

$$D_{KL}(P\parallel Q)= \sum_{i=1}^{k} P(x)log(\frac{P(x)}{Q(x)})$$

Where P(x) and Q(x) stand for the probability of x in the distribution of P and Q, respectively.

As log transformed fold changes (LFC) approximately follows a to a zero-centered normal distribution (3), thus we replace the P(x) and Q(x) with the LFC in the gene expression of medication and disease.

So, the regulation score is defined as:

$$RS= \sum_{i=1}^{k} \rho_{({FC}_{k}^{GBM})}log(\frac{{FC}_{k}^{drug}}{{FC}_{k}^{GBM}})$$

, where

$$\rho_{{(FC}_{k}^{GBM})}=\frac{|{FC}_{k}^{GBM}|}{\sum_{i=1}^{n} |{FC}_{i}^{GBM}|}$$

The FC in all formulas, unless specified, refers to log transformed fold change.

Here we also changed the P(x) out the log with formula 3 to weight the gene’s significance in all disease-related genes, which is associated with its fold change. In this formula, changes of genes with bigger fold changes in disease have higher regulation scores.

As the fold changes obtained from DE analysis have already been log transformed (range from -∞ ~ ∞), so the formula then becomes:

$$RS= \sum_{i=1}^{k} \rho_{\left( {LFC}_{k}^{GBM} \right)} \left( {LFC}_{k}^{drug}-L{FC}_{k}^{GBM} \right)$$

$$= \sum_{i=1}^{k} \left( L{FC}_{k}^{drug}-{LFC}_{k}^{GBM} \right) \frac{|{LFC}_{k}^{GBM}|}{\sum_{i=1}^{n} |L{FC}_{i}^{GBM}|}$$

Then, no regulation effect when the ${FC}_{k}^{drug}$ and ${FC}_{k}^{GBM}$ are in the same direction, i.e., both positive or both negative. Thus, the final regulation score was calculated when they are in different directions, i.e.,

$$RS= \sum_{i=1}^{k} \Delta_{LFC} \frac{|{LFC}_{k}^{GBM}|}{\sum_{i=1}^{n} |{LFC}_{i}^{GBM}|}$$

$${where \Delta}_{LFC}= \left\{ \begin{aligned} \left| \left( {LFC}_{k}^{drug}-{LFC}_{k}^{GBM} \right) \right|, if {(LFC}_{k}^{drug}L{FC}_{k}^{GBM})<0 \\ 0, if {(LFC}_{k}^{drug}{LFC}_{k}^{GBM})>0 \end{aligned} \right.$$

Geometric interpretation of $\Delta_{LFC}$:


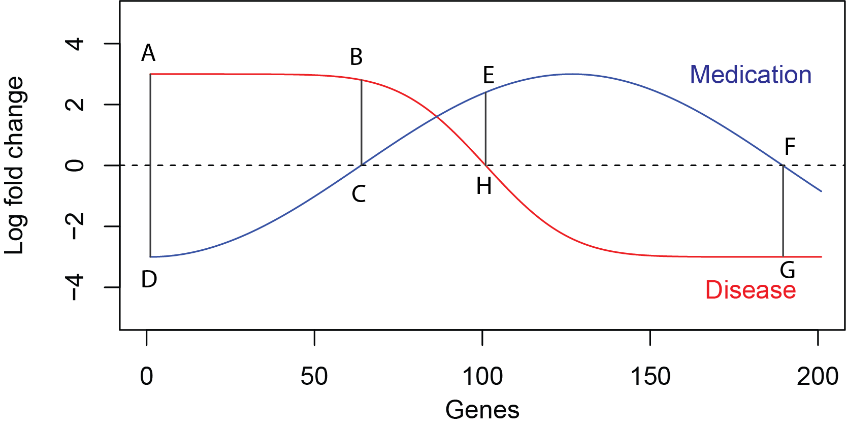
This figure illustrates the geometric interpretation of $\Delta_{LFC}$. The blue line stands for the LFC in each gene caused by a medicine, while the red line stands for the LFC of the DE genes. the area of ABCD and EFGH are calculated. In both areas, the medication reverses the expression of disease associated genes. The area is impacted by numbers of genes (the width in x axis) and the LFC (the height in y axis).

Features of regulation score:

From the above figure, we reason that the feature of the regulation score makes it an appropriate indictor for drug repurposing candidate evaluation.

1. It ranges from 0 ~ ∞. The score is 0 when a drug can regulate no disease related genes.
2. It increases with the number of regulated genes, so this avoids the issue of the Spearmen correlation.
3. It increases with the increase in strength to which extend a drug can regulate the gene (represented by the fold change value)
4. Genes with higher significance in disease (represented by higher fold change, which indicates more significant impact on overall biological functions) are assigned a higher weight during the calculation.
5. Compare with similarity score based on Gene set enrichment score (GSEA): the GSEA also rank the genes based on fold change,
6. Un-regulated genes are not taken into consideration. Effects and side effects cannot be mixed together, instead, they should be evaluated separately. – MRS.

A higher regulation score will be resulted when a medicine has 1) a bigger number of regulated genes, 2) higher fold changes when regulating these genes, and 3) these regulated genes have higher importance in the disease mechanism.


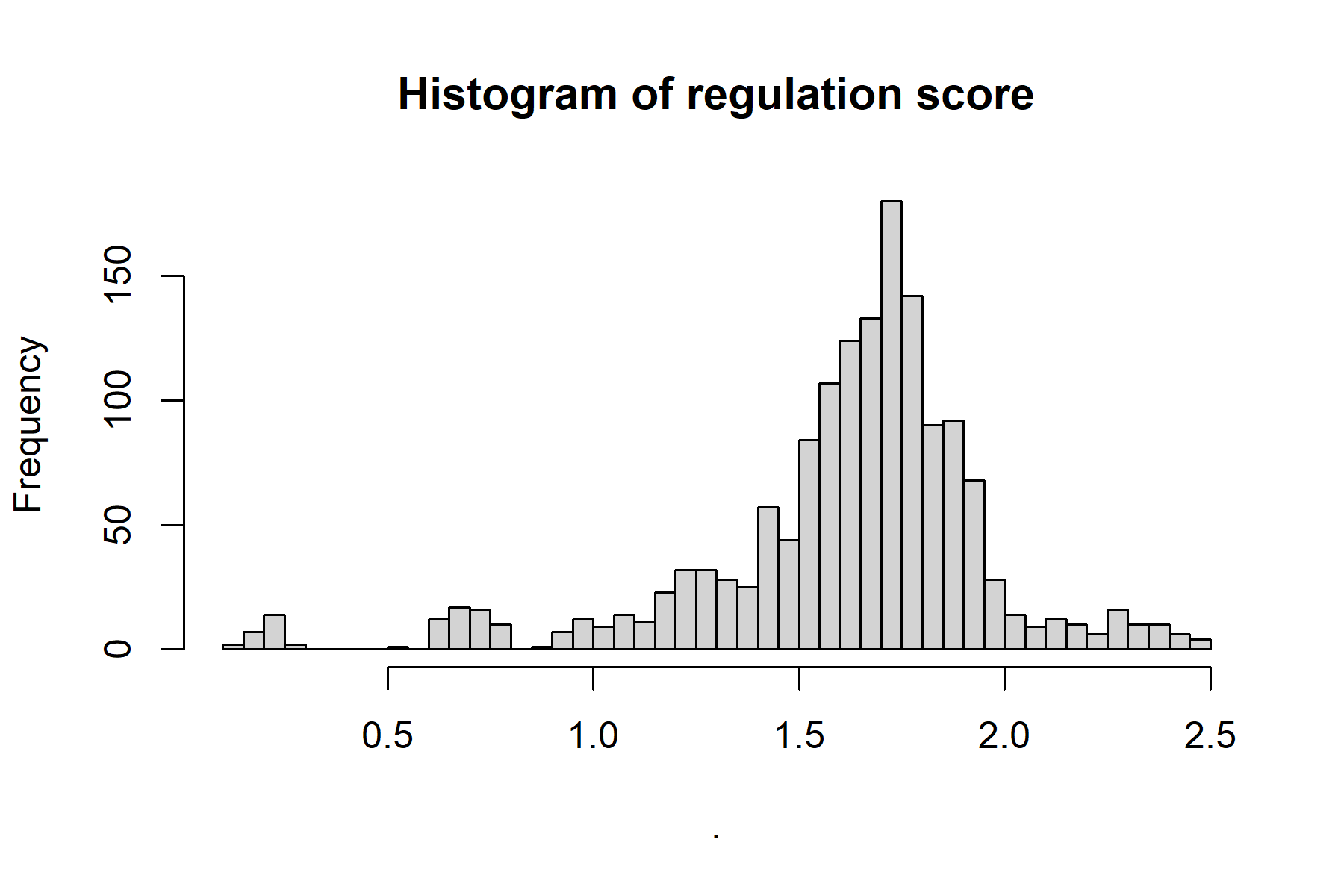


1. Kullback S, Leibler RA. On Information and Sufficiency. The Annals of Mathematical Statistics. 1951;22(1):79-86, 8.

2. Shannon CE. A mathematical theory of communication. The Bell System Technical Journal. 1948;27(3):379-423.

3. Love MI, Huber W, Anders S. Moderated estimation of fold change and dispersion for RNA-seq data with DESeq2. Genome Biol. 2014;15(12):550.
