# Supplementary material for: Reversal gene expression assessment for drug repurposing, a case study of glioblastoma
Source: J Transl Med. 2025 Jan 7;23:25. doi: 10.1186/s12967-024-06046-1 (PMC11706105; doi:10.1186/s12967-024-06046-1)
Supplement: Supplementary file 16 — Additional file 16 [file 12967_2024_6046_MOESM16_ESM.pdf]

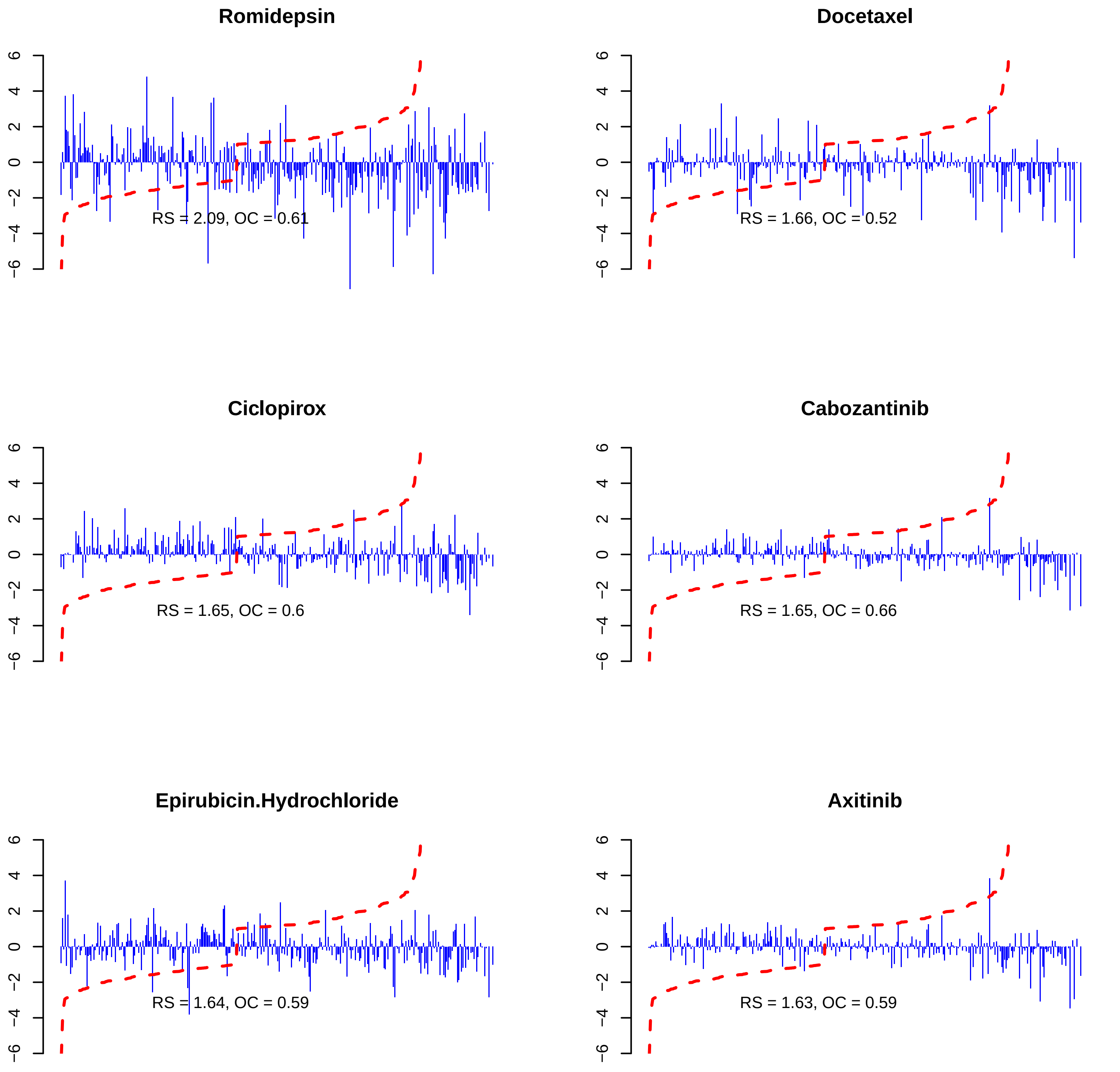
Romidepsin
RS = 2.09, OC = 0.61
Docetaxel
RS = 1.66, OC = 0.52
Ciclopirox
RS = 1.65, OC = 0.6
Cabozantinib
RS = 1.65, OC = 0.66
Epirubicin.Hydrochloride
RS = 1.64, OC = 0.59
Axitinib
RS = 1.63, OC = 0.59

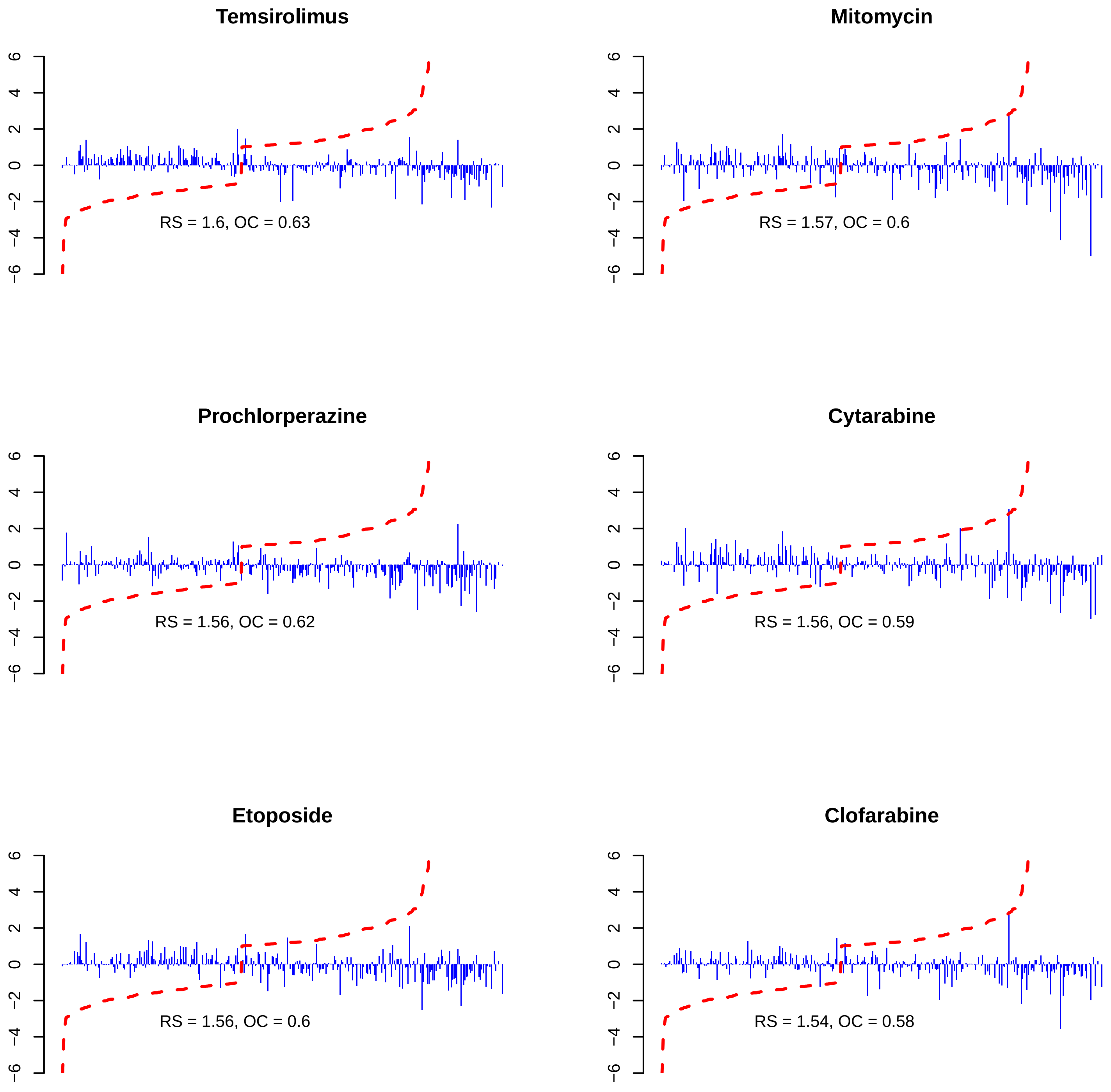
Temsirolimus
RS = 1.6, OC = 0.63
Mitomycin
RS = 1.57, OC = 0.6
Prochlorperazine
RS = 1.56, OC = 0.62
Cytarabine
RS = 1.56, OC = 0.59
Etoposide
RS = 1.56, OC = 0.6
Clofarabine
RS = 1.54, OC = 0.58
6
4
2
0
−2
−4
−6

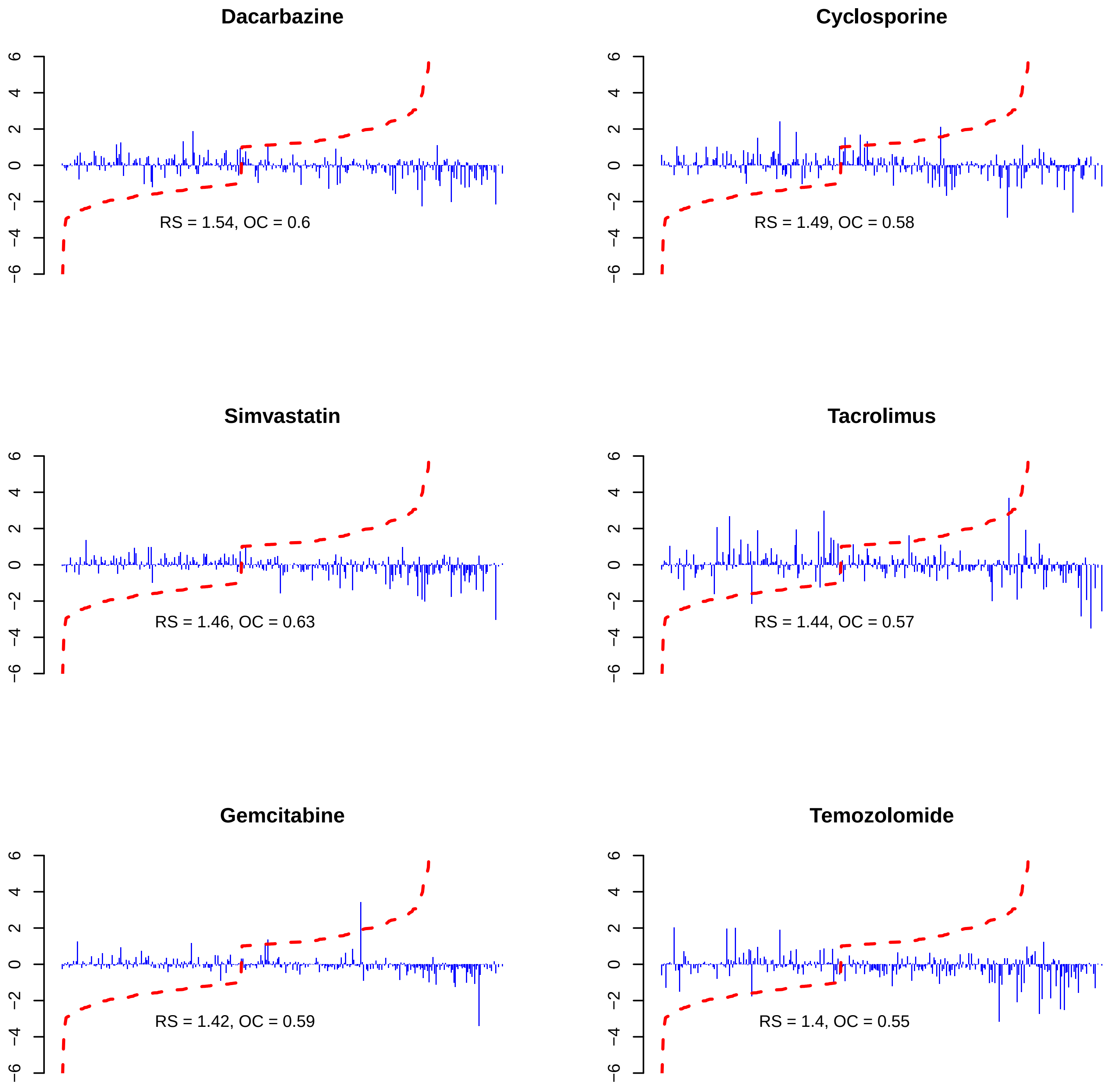
Dacarbazine
RS = 1.54, OC = 0.6
Cyclosporine
RS = 1.49, OC = 0.58
Simvastatin
RS = 1.46, OC = 0.63
Tacrolimus
RS = 1.44, OC = 0.57
Gemcitabine
RS = 1.42, OC = 0.59
Temozolomide
RS = 1.4, OC = 0.55

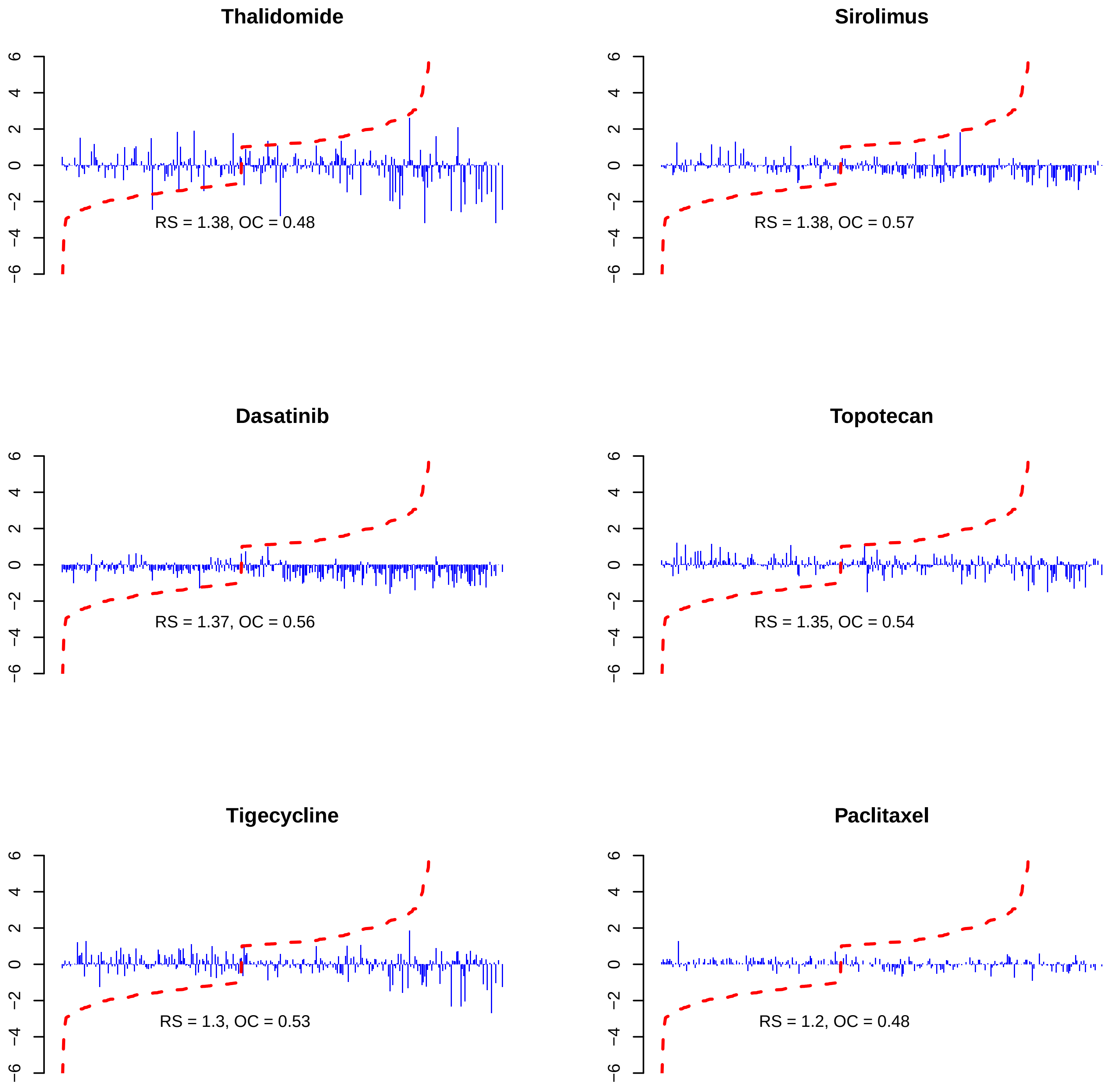
Thalidomide
RS = 1.38, OC = 0.48
Sirolimus
RS = 1.38, OC = 0.57
Dasatinib
RS = 1.37, OC = 0.56
Topotecan
RS = 1.35, OC = 0.54
Tigecycline
RS = 1.3, OC = 0.53
Paclitaxel
RS = 1.2, OC = 0.48
6
4
2
0
−2
−4
−6

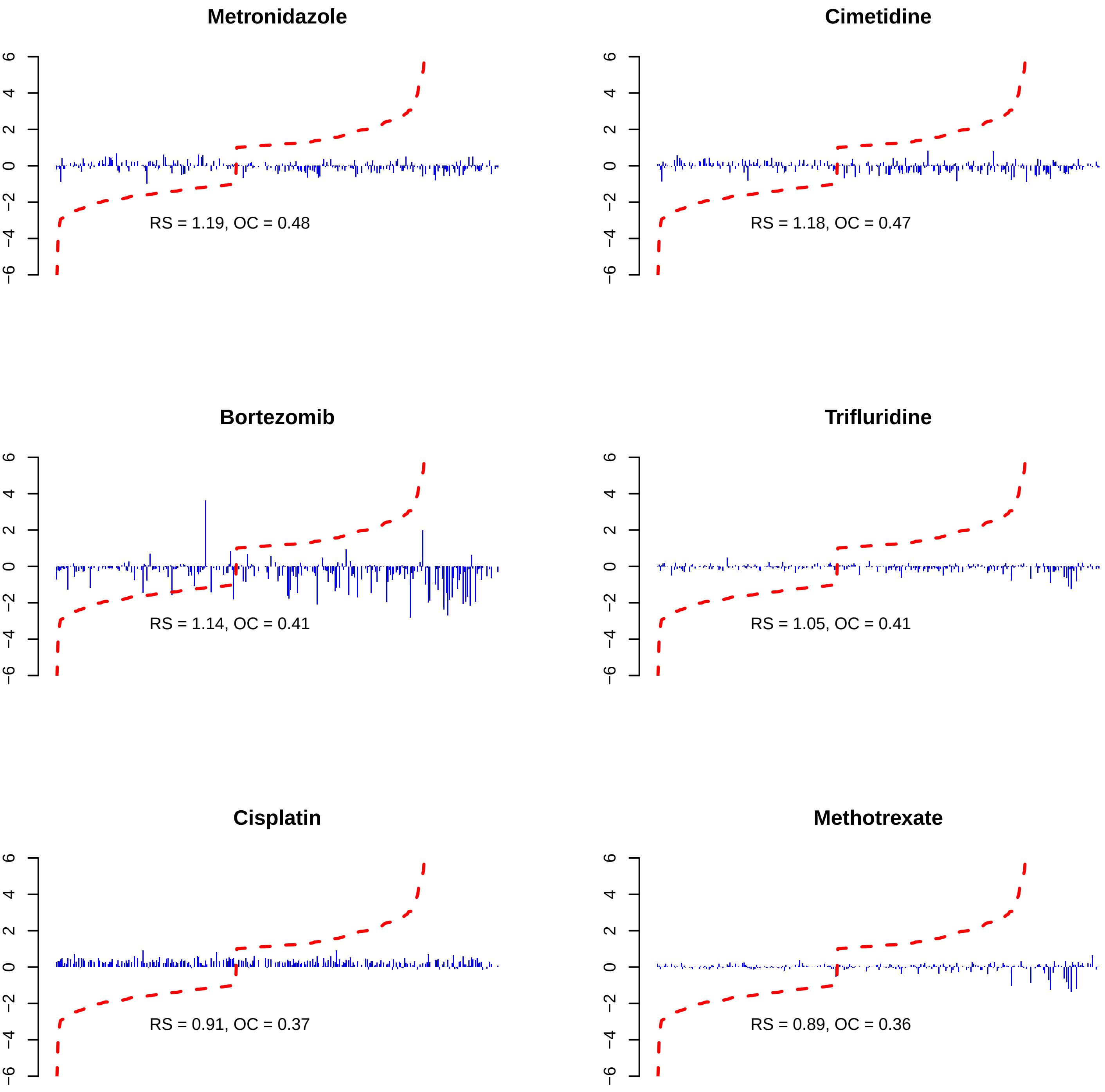
Metronidazole
RS = 1.19, OC = 0.48
Cimetidine
RS = 1.18, OC = 0.47
Bortezomib
RS = 1.14, OC = 0.41
Trifluridine
RS = 1.05, OC = 0.41
Cisplatin
RS = 0.91, OC = 0.37
Methotrexate
RS = 0.89, OC = 0.36
6
4
2
0
−2
−4
−6

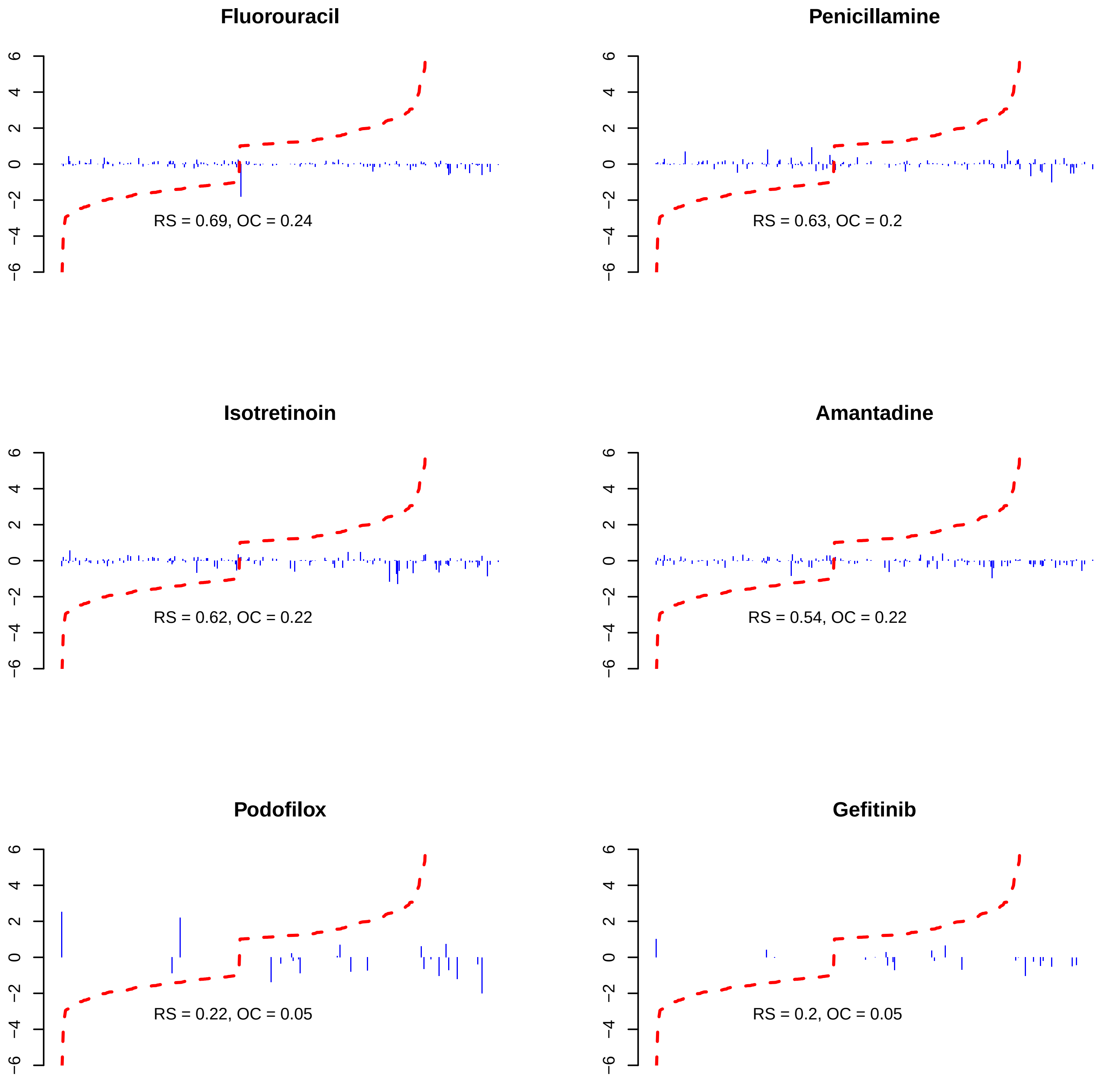
Fluorouracil
RS = 0.69, OC = 0.24
Penicillamine
RS = 0.63, OC = 0.2
Isotretinoin
RS = 0.62, OC = 0.22
Amantadine
RS = 0.54, OC = 0.22
Podofilox
RS = 0.22, OC = 0.05
Gefitinib
RS = 0.2, OC = 0.05

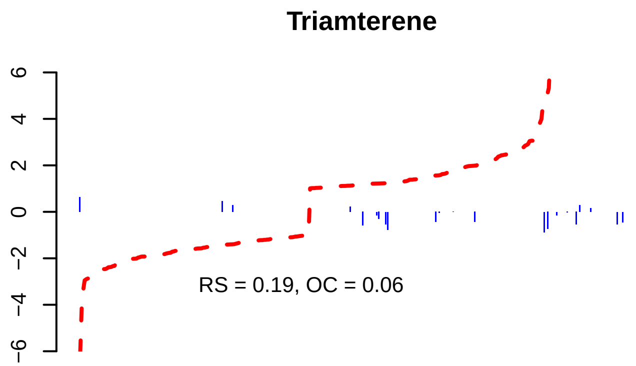
Triamterene
6
4
2
0
−2
−4
−6
RS = 0.19, OC = 0.06

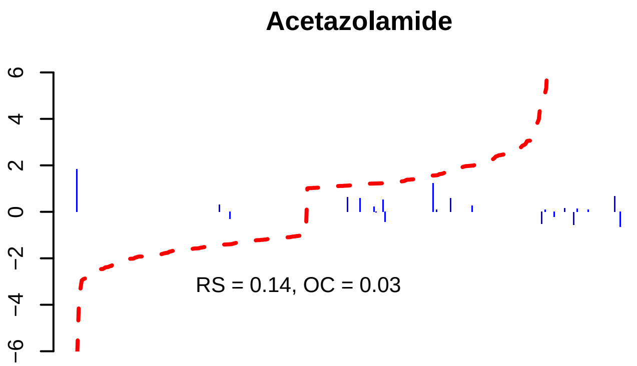
Acetazolamide
6
4
2
0
−2
−4
−6
RS = 0.14, OC = 0.03

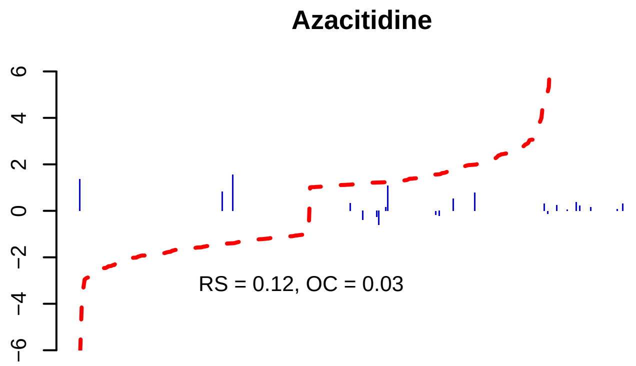
Azacitidine
6
4
2
0
−2
−4
−6
RS = 0.12, OC = 0.03
