# Supplementary material for: Reversal gene expression assessment for drug repurposing, a case study of glioblastoma
Source: J Transl Med. 2025 Jan 7;23:25. doi: 10.1186/s12967-024-06046-1 (PMC11706105; doi:10.1186/s12967-024-06046-1)
Supplement: Supplementary file 17 — Additional file 17 [file 12967_2024_6046_MOESM17_ESM.pdf]

ARAX User Interface

Documentation: Overview TRAPI 1.4.2 Resources

Input

Queries

Settings

List A 0

List B 0

Compare Lists

History 3

Output

Summary

Provenance

Knowledge Graph

Results 12

Messages 1204

Tools

Synonyms

Dev Info

System Activity

SmartAPI Info

Translator Testing

Reset All

Late Wildfowl

KNOWLEDGE GRAPH

↻
B
F
C
R

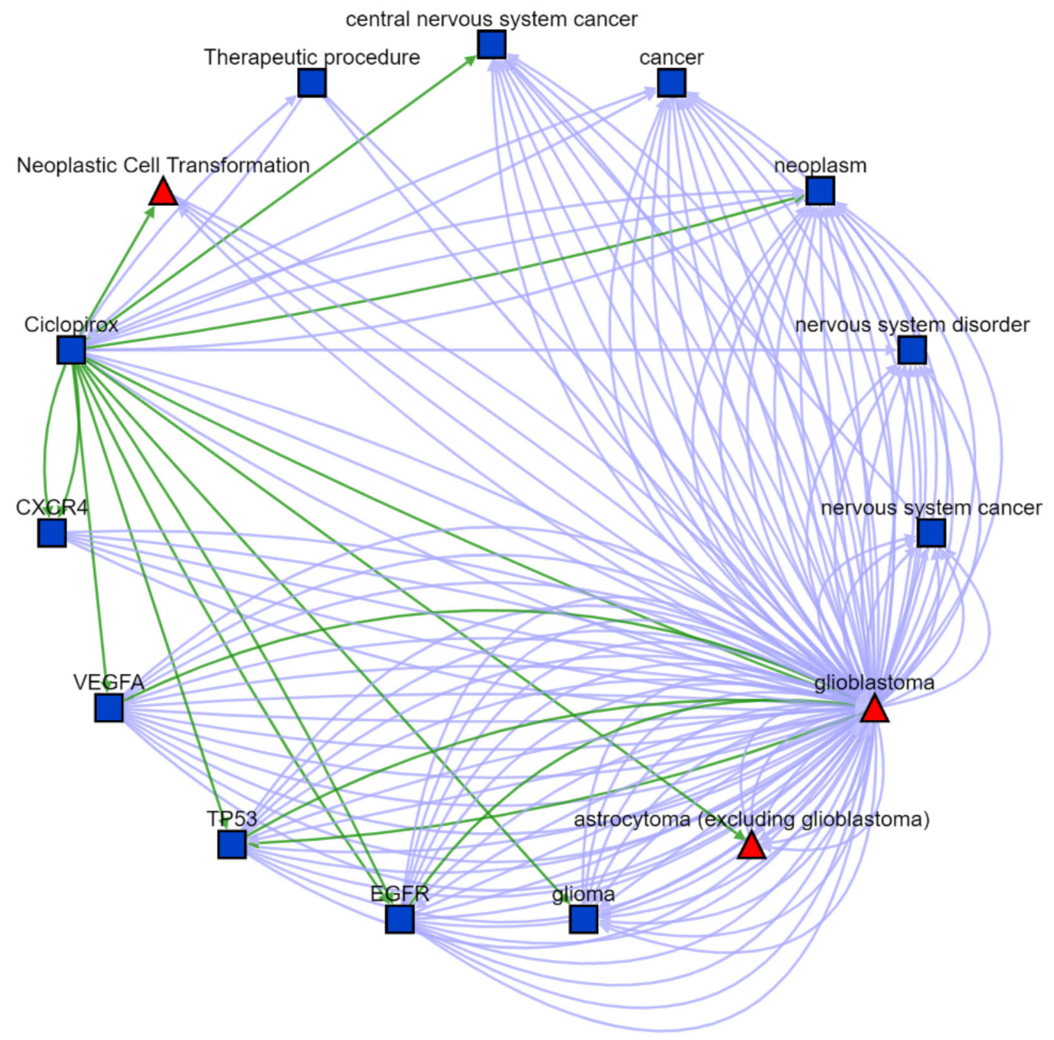

All nodes and edges:

MONDO:0002714 *central nervous system cancer*

biolink:subclass_of →	MONDO:0005872
biolink:coexists_with →	MONDO:0018177
← biolink:subclass_of	MONDO:0018177

Input

Queries

Settings

List A 0

List B 0

Compare Lists

History 3

Output

Summary

Provenance

Knowledge Graph

Results 12

Messages 1204

Tools

Synonyms

Dev Info

System Activity

SmartAPI Info

Translator Testing

Reset All

Late Wildfowl

	[illegible]	[illegible]
	← biolink:subclass_of	MONDO:0018177
	← biolink:subclass_of	MONDO:0018177
	← biolink:subclass_of	MONDO:0018177
	← biolink:associated_with [q]	PUBCHEM.COMPOUND:2749
	← biolink:subclass_of	MONDO:0018177
**MONDO:0004992**	*cancer*	
	biolink:affects →	MONDO:0018177
	biolink:causes →	MONDO:0018177
	biolink:coexists_with →	MONDO:0018177
	← biolink:subclass_of	MONDO:0018177
	← biolink:subclass_of	MONDO:0018177
	← biolink:subclass_of	MONDO:0018177
	← biolink:subclass_of	MONDO:0018177
	← biolink:subclass_of	MONDO:0018177
	← biolink:treats	PUBCHEM.COMPOUND:2749
	← biolink:subclass_of	MONDO:0018177
	← biolink:affects	MONDO:0018177
	← biolink:coexists_with	MONDO:0018177
	← biolink:subclass_of	MONDO:0018177
	← biolink:occurs_together_in_literature_with	PUBCHEM.COMPOUND:2749
**MONDO:0005070**	*neoplasm*	
	biolink:causes →	MONDO:0018177
	← biolink:subclass_of	MONDO:0018177
	← biolink:subclass_of	MONDO:0018177
	← biolink:subclass_of	MONDO:0018177
	← biolink:subclass_of	MONDO:0018177
	← biolink:subclass_of	MONDO:0018177
	← biolink:treats	PUBCHEM.COMPOUND:2749
	← biolink:subclass_of	MONDO:0018177
	← biolink:associated_with [q]	PUBCHEM.COMPOUND:2749
	← biolink:affects	MONDO:0018177
	← biolink:causes	MONDO:0018177
	← biolink:coexists_with	MONDO:0018177
	← biolink:manifestation_of	MONDO:0018177
	← biolink:subclass_of	MONDO:0018177
	← biolink:occurs_together_in_literature_with	PUBCHEM.COMPOUND:2749
	← biolink:occurs_together_in_literature_with	PUBCHEM.COMPOUND:2749
**MONDO:0005071**	*nervous system disorder*	
	← biolink:subclass_of	MONDO:0018177
	← biolink:subclass_of	MONDO:0018177

ARAX User Interface

Documentation: Overview TRAPI 1.4.2 Resources

Input
Queries
Settings
List A 0
List B 0
Compare Lists
History 3

Output
Summary
Provenance
Knowledge Graph
Results 12
Messages 1204

Tools
Synonyms
Dev Info
System Activity
SmartAPI Info
Translator Testing
Reset All

Late Wildfowl

		[illegible]	[illegible]
		← biolink:subclass_of	MONDO:0018177
		← biolink:subclass_of	MONDO:0018177
		← biolink:occurs_together_in_literature_with	PUBCHEM.COMPOUND:2749
**MONDO:0005872**	*nervous system cancer*		
		← biolink:subclass_of	MONDO:0018177
		← biolink:subclass_of	MONDO:0018177
		← biolink:subclass_of	MONDO:0018177
		← biolink:subclass_of	MONDO:0018177
		← biolink:subclass_of	MONDO:0018177
		← biolink:subclass_of	MONDO:0018177
		← biolink:subclass_of	MONDO:0002714
		← biolink:subclass_of	MONDO:0018177
**MONDO:0018177**	*glioblastoma*		
		biolink:subclass_of →	MONDO:0002714
		biolink:subclass_of →	MONDO:0004992
		biolink:subclass_of →	MONDO:0005070
		biolink:subclass_of →	MONDO:0005071
		biolink:subclass_of →	MONDO:0005872
		biolink:subclass_of →	MONDO:0021042
		biolink:subclass_of →	MONDO:0002714
		biolink:subclass_of →	MONDO:0004992
		biolink:subclass_of →	MONDO:0005070
		biolink:subclass_of →	MONDO:0005071
		biolink:subclass_of →	MONDO:0005872
		biolink:subclass_of →	MONDO:0021042
		biolink:subclass_of →	MONDO:0002714
		biolink:subclass_of →	MONDO:0002714
		biolink:subclass_of →	MONDO:0004992
		biolink:subclass_of →	MONDO:0005070
		biolink:subclass_of →	MONDO:0005071
		biolink:subclass_of →	MONDO:0005872
		biolink:subclass_of →	MONDO:0021042
		biolink:subclass_of →	MONDO:0002714
		biolink:subclass_of →	MONDO:0004992
		biolink:subclass_of →	MONDO:0005070
		biolink:subclass_of →	MONDO:0005071
		biolink:subclass_of →	MONDO:0005872
		biolink:subclass_of →	MONDO:0021042
		biolink:subclass_of →	MONDO:0002714
		biolink:subclass_of →	MONDO:0004992
		biolink:subclass_of →	MONDO:0005070

Input
Queries
Settings
List A 0
List B 0
Compare Lists
History 3

Output
Summary
Provenance
Knowledge Graph
Results 12
Messages 1204

Tools
Synonyms
Dev Info
System Activity
SmartAPI Info
Translator Testing
Reset All

Late Wildfowl

[illegible]	[illegible]
biolink:subclass_of →	MONDO:0002714
biolink:subclass_of →	MONDO:0004992
biolink:subclass_of →	MONDO:0005070
biolink:subclass_of →	MONDO:0005071
biolink:subclass_of →	MONDO:0005872
biolink:subclass_of →	MONDO:0021042
biolink:has_participant [q] →	NCBIGene:7157
biolink:affects →	MONDO:0004992
biolink:affects →	MONDO:0005070
biolink:affects →	UMLS:C0007621
biolink:causes →	MONDO:0005070
biolink:coexists_with →	MONDO:0004992
biolink:coexists_with →	MONDO:0005070
biolink:coexists_with →	MONDO:0019781
biolink:coexists_with →	MONDO:0021042
biolink:coexists_with →	UMLS:C0007621
biolink:has_part →	NCBIGene:7157
biolink:manifestation_of →	MONDO:0005070
biolink:produces →	NCBIGene:1956
biolink:produces →	NCBIGene:7422
biolink:subclass_of →	MONDO:0002714
biolink:subclass_of →	MONDO:0004992
biolink:subclass_of →	MONDO:0005070
biolink:subclass_of →	MONDO:0005071
biolink:subclass_of →	MONDO:0005872
biolink:subclass_of →	MONDO:0019781
biolink:subclass_of →	MONDO:0019781
biolink:subclass_of →	MONDO:0019781
biolink:subclass_of →	MONDO:0019781
biolink:subclass_of →	MONDO:0021042
biolink:subclass_of →	MONDO:0021042
biolink:subclass_of →	MONDO:0021042
← biolink:genetic_association	NCBIGene:1956
← biolink:genetic_association	NCBIGene:1956
← biolink:genetic_association	NCBIGene:1956
← biolink:genetic_association	NCBIGene:1956
← biolink:genetic_association	NCBIGene:7157
← biolink:genetic_association	NCBIGene:7157
← biolink:genetic_association	NCBIGene:7422
← biolink:genetic_association	NCBIGene:7422
← biolink:genetic_association	NCBIGene:7422
← biolink:genetic_association	NCBIGene:7422
← biolink:contributes_to	NCBIGene:1956

Input

Queries

Settings

List A 0

List B 0

Compare Lists

History 3

Output

Summary

Provenance

Knowledge Graph

Results 12

Messages 1204

Tools

Synonyms

Dev Info

System Activity

SmartAPI Info

Translator Testing

Reset All

Late Wildfowl

←	biolink:genetically_associated_with	NCBIGene:1956
←	biolink:genetically_associated_with	NCBIGene:1956
←	biolink:contributes_to	NCBIGene:7157
←	biolink:genetically_associated_with	NCBIGene:7157
←	biolink:genetically_associated_with	NCBIGene:7157
←	biolink:contributes_to	NCBIGene:7422
←	biolink:genetically_associated_with	NCBIGene:7422
←	biolink:genetically_associated_with	NCBIGene:7422
←	biolink:genetically_associated_with	NCBIGene:7422
←	biolink:genetically_associated_with	NCBIGene:7422
←	biolink:contributes_to	NCBIGene:7852
←	biolink:treats	PUBCHEM.COMPOUND:2749
←	biolink:associated_with [q]	PUBCHEM.COMPOUND:2749
←	biolink:coexists_with	MONDO:0002714
←	biolink:affects	MONDO:0004992
←	biolink:causes	MONDO:0004992
←	biolink:coexists_with	MONDO:0004992
←	biolink:causes	MONDO:0005070
←	biolink:coexists_with	MONDO:0019781
←	biolink:coexists_with	MONDO:0021042
←	biolink:affects	NCBIGene:1956
←	biolink:affects [q]	NCBIGene:1956
←	biolink:gene_associated_with_condition	NCBIGene:1956
←	biolink:gene_associated_with_condition	NCBIGene:1956
←	biolink:gene_associated_with_condition	NCBIGene:1956
←	biolink:related_to	NCBIGene:1956
←	biolink:related_to	NCBIGene:1956
←	biolink:affects	NCBIGene:7157
←	biolink:affects [q]	NCBIGene:7157
←	biolink:causes	NCBIGene:7157
←	biolink:gene_associated_with_condition	NCBIGene:7157
←	biolink:gene_associated_with_condition	NCBIGene:7157
←	biolink:related_to	NCBIGene:7157
←	biolink:affects [q]	NCBIGene:7422
←	biolink:gene_associated_with_condition	NCBIGene:7422
←	biolink:gene_associated_with_condition	NCBIGene:7422
←	biolink:related_to	NCBIGene:7422
←	biolink:affects	NCBIGene:7852
←	biolink:gene_associated_with_condition	NCBIGene:7852
←	biolink:gene_associated_with_condition	NCBIGene:7852
←	biolink:related_to	NCBIGene:7852
←	biolink:causes	UMLS:C0007621
←	biolink:coexists_with	UMLS:C0007621
←	biolink:prevents	UMLS:C0087111

Input
Queries
Settings
List A 0
List B 0
Compare Lists
History 3

Output
Summary
Provenance
Knowledge Graph
Results 12
Messages 1204

Tools
Synonyms
Dev Info
System Activity
SmartAPI Info
Translator Testing
Reset All

Late Wildfowl

		← biolink:treats	PUBCHEM.COMPOUND:[illegible]

**MONDO:0019781**	*astrocytoma (excluding glioblastoma)*		
		biolink:coexists_with →	MONDO:0018177
		← biolink:associated_with [q]	PUBCHEM.COMPOUND:2749
		← biolink:coexists_with	MONDO:0018177
		← biolink:subclass_of	MONDO:0018177
		← biolink:subclass_of	MONDO:0018177
		← biolink:subclass_of	MONDO:0018177
		← biolink:subclass_of	MONDO:0018177

**MONDO:0021042**	*glioma*		
		biolink:coexists_with →	MONDO:0018177
		← biolink:subclass_of	MONDO:0018177
		← biolink:subclass_of	MONDO:0018177
		← biolink:subclass_of	MONDO:0018177
		← biolink:subclass_of	MONDO:0018177
		← biolink:subclass_of	MONDO:0018177
		← biolink:subclass_of	MONDO:0018177
		← biolink:associated_with [q]	PUBCHEM.COMPOUND:2749
		← biolink:coexists_with	MONDO:0018177
		← biolink:subclass_of	MONDO:0018177
		← biolink:subclass_of	MONDO:0018177
		← biolink:subclass_of	MONDO:0018177

**NCBIGene:1956**	*EGFR*		
		biolink:genetic_association →	MONDO:0018177
		biolink:genetic_association →	MONDO:0018177
		biolink:genetic_association →	MONDO:0018177
		biolink:genetic_association →	MONDO:0018177
		biolink:contributes_to →	MONDO:0018177
		biolink:genetically_associated_with →	MONDO:0018177
		biolink:genetically_associated_with →	MONDO:0018177
		biolink:genetically_associated_with →	MONDO:0018177
		biolink:genetically_associated_with →	MONDO:0018177
		biolink:affects →	MONDO:0018177
		biolink:affects [q] →	MONDO:0018177
		biolink:gene_associated_with_condition →	MONDO:0018177
		biolink:gene_associated_with_condition →	MONDO:0018177
		biolink:gene_associated_with_condition →	MONDO:0018177
		biolink:related_to →	MONDO:0018177
		biolink:related_to →	MONDO:0018177
		← biolink:regulates [q]	PUBCHEM.COMPOUND:2749
		← biolink:regulates [q]	PUBCHEM.COMPOUND:2749

Input
Queries
Settings
List A 0
List B 0
Compare Lists
History 3
Output
Summary
Provenance
Knowledge Graph
Results 12
Messages 1204
Tools
Synonyms
Dev Info
System Activity
SmartAPI Info
Translator Testing
Reset All
Late Wildfowl

	biolink:genetic_association →	MONDO:0018177
	biolink:genetic_association →	MONDO:0018177
	biolink:contributes_to →	MONDO:0018177
	biolink:genetically_associated_with →	MONDO:0018177
	biolink:genetically_associated_with →	MONDO:0018177
	biolink:affects →	MONDO:0018177
	biolink:affects [q] →	MONDO:0018177
	biolink:causes →	MONDO:0018177
	biolink:gene_associated_with_condition →	MONDO:0018177
	biolink:gene_associated_with_condition →	MONDO:0018177
	biolink:related_to →	MONDO:0018177
	← biolink:has_participant [q]	MONDO:0018177
	← biolink:affects [q]	PUBCHEM.COMPOUND:2749
	← biolink:has_part	MONDO:0018177

**NCBIGene:7422**	*VEGFA*	
	biolink:genetic_association →	MONDO:0018177
	biolink:genetic_association →	MONDO:0018177
	biolink:genetic_association →	MONDO:0018177
	biolink:genetic_association →	MONDO:0018177
	biolink:contributes_to →	MONDO:0018177
	biolink:genetically_associated_with →	MONDO:0018177
	biolink:genetically_associated_with →	MONDO:0018177
	biolink:genetically_associated_with →	MONDO:0018177
	biolink:genetically_associated_with →	MONDO:0018177
	biolink:affects [q] →	MONDO:0018177
	biolink:gene_associated_with_condition →	MONDO:0018177
	biolink:gene_associated_with_condition →	MONDO:0018177
	biolink:related_to →	MONDO:0018177
	← biolink:affects [q]	PUBCHEM.COMPOUND:2749
	← biolink:produces	MONDO:0018177

**NCBIGene:7852**	*CXCR4*	
	biolink:contributes_to →	MONDO:0018177
	biolink:affects →	MONDO:0018177
	biolink:gene_associated_with_condition →	MONDO:0018177
	biolink:gene_associated_with_condition →	MONDO:0018177
	biolink:related_to →	MONDO:0018177
	← biolink:regulates [q]	PUBCHEM.COMPOUND:2749
	← biolink:regulates [q]	PUBCHEM.COMPOUND:2749

**PUBCHEM.COMPOUND:2749**	*Ciclopirox*	
	biolink:regulates [q] →	NCBIGene:1956

Input
Queries
Settings
List A 0
List B 0
Compare Lists
History 3
Output
Summary
Provenance
Knowledge Graph
Results 12
Messages 1204
Tools
Synonyms
Dev Info
System Activity
SmartAPI Info
Translator Testing
Reset All
Late Wildfowl

[illegible]	[illegible]
biolink:regulates [q] →	NCBIGene:7852
biolink:treats →	MONDO:0004992
biolink:treats →	MONDO:0005070
biolink:treats →	MONDO:0018177
biolink:affects [q] →	NCBIGene:7157
biolink:associated_with [q] →	MONDO:0002714
biolink:associated_with [q] →	MONDO:0005070
biolink:associated_with [q] →	MONDO:0018177
biolink:associated_with [q] →	MONDO:0019781
biolink:associated_with [q] →	MONDO:0021042
biolink:associated_with [q] →	UMLS:C0007621
biolink:subclass_of →	UMLS:C0087111
biolink:has_adverse_event →	MONDO:0018177
biolink:treats →	MONDO:0018177
biolink:occurs_together_in_literature_with →	MONDO:0005070
biolink:occurs_together_in_literature_with →	MONDO:0004992
biolink:occurs_together_in_literature_with →	MONDO:0005070
biolink:occurs_together_in_literature_with →	MONDO:0005071
← biolink:has_input	UMLS:C0087111

UMLS:C0007621 *Neoplastic Cell Transformation*

biolink:causes →	MONDO:0018177
biolink:coexists_with →	MONDO:0018177
← biolink:associated_with [q]	PUBCHEM.COMPOUND:2749
← biolink:affects	MONDO:0018177
← biolink:coexists_with	MONDO:0018177

UMLS:C0087111 *Therapeutic procedure*

biolink:has_input →	PUBCHEM.COMPOUND:2749
	[illegible]

ARAX User Interface

Documentation: Overview TRAPI 1.4.2 Resources

Input

Queries

Settings

List A 0

List B 0

Compare Lists

History 3

Output

Summary

Provenance

Knowledge Graph

Results 12

Messages 1204

Tools

Synonyms

Dev Info

System Activity

SmartAPI Info

Translator Testing

Reset All

Late Wildfowl

ARAX User Interface

Documentation: Overview TRAPI 1.4.2 Resources

Input

Queries

Settings

List A 0

List B 0

Compare Lists

History 3

Output

Summary

Provenance

Knowledge Graph

Results 12

Messages 1204

Tools

Synonyms

Dev Info

System Activity

SmartAPI Info

Translator Testing

Reset All

Late Wildfowl

ARAX User Interface

Documentation: Overview TRAPI 1.4.2 Resources

Input

Queries

Settings

List A 0

List B 0

Compare Lists

History 3

Output

Summary

Provenance

Knowledge Graph

Results 12

Messages 1204

Tools

Synonyms

Dev Info

System Activity

SmartAPI Info

Translator Testing

Reset All

Late Wildfowl

ARAX User Interface

Documentation: Overview TRAPI 1.4.2 Resources

Input

Queries

Settings

List A 0

List B 0

Compare Lists

History 3

Output

Summary

Provenance

Knowledge Graph

Results 12

Messages 1204

Tools

Synonyms

Dev Info

System Activity

SmartAPI Info

Translator Testing

Reset All

Late Wildfowl

ARAX User Interface

Documentation: Overview TRAPI 1.4.2 Resources

Input

Queries

Settings

List A 0

List B 0

Compare Lists

History 3

Output

Summary

Provenance

Knowledge Graph

Results 12

Messages 1204

Tools

Synonyms

Dev Info

System Activity

SmartAPI Info

Translator Testing

Reset All

Late Wildfowl

ARAX User Interface

Documentation: Overview TRAPI 1.4.2 Resources

Input

Queries

Settings

List A 0

List B 0

Compare Lists

History 3

Output

Summary

Provenance

Knowledge Graph

Results 12

Messages 1204

Tools

Synonyms

Dev Info

System Activity

SmartAPI Info

Translator Testing

Reset All

Late Wildfowl

ARAX User Interface

Documentation: Overview TRAPI 1.4.2 Resources

Input

Queries

Settings

List A 0

List B 0

Compare Lists

History 3

Output

Summary

Provenance

Knowledge Graph

Results 12

Messages 1204

Tools

Synonyms

Dev Info

System Activity

SmartAPI Info

Translator Testing

Reset All

Late Wildfowl

ARAX User Interface

Documentation: Overview TRAPI 1.4.2 Resources

Input

Queries

Settings

List A 0

List B 0

Compare Lists

History 3

Output

Summary

Provenance

Knowledge Graph

Results 12

Messages 1204

Tools

Synonyms

Dev Info

System Activity

SmartAPI Info

Translator Testing

Reset All

Late Wildfowl

ARAX User Interface

Documentation: Overview TRAPI 1.4.2 Resources

Input

Queries

Settings

List A 0

List B 0

Compare Lists

History 3

Output

Summary

Provenance

Knowledge Graph

Results 12

Messages 1204

Tools

Synonyms

Dev Info

System Activity

SmartAPI Info

Translator Testing

Reset All

Late Wildfowl
